# Pigmentary mosaicism: a review of original literature and recommendations for future handling

**DOI:** 10.1186/s13023-018-0778-6

**Published:** 2018-03-05

**Authors:** Anna Boye Kromann, Lilian Bomme Ousager, Inas Kamal Mohammad Ali, Nurcan Aydemir, Anette Bygum

**Affiliations:** 1 Department of Dermatology and Allergy Centre, J.B. Winsløws Vej 4 , Entrance 142, 5000 Odense C, Denmark; 2 Department of Clinical Genetics, J.B. Winsløws Vej 4, Entrance 24, 5000 Odense C, Denmark

**Keywords:** Pigmentary mosaicism, Blaschko’s lines, Hypopigmentation, Hyperpigmentation, Linear and whorled nevoid hypermelanosis, Hypomelanosis of Ito

## Abstract

**Background:**

Pigmentary mosaicism is a term that describes varied patterns of pigmentation in the skin caused by genetic heterogeneity of the skin cells. In a substantial number of cases, pigmentary mosaicism is observed alongside extracutaneous abnormalities typically involving the central nervous system and the musculoskeletal system. We have compiled information on previous cases of pigmentary mosaicism aiming to optimize the handling of patients with this condition. Our study is based on a database search in PubMed containing papers written in English, published between January 1985 and April 2017. The search yielded 174 relevant and original articles, detailing a total number of 651 patients.

**Results:**

Forty-three percent of the patients exhibited hyperpigmentation, 50% exhibited hypopigmentation, and 7% exhibited a combination of hyperpigmentation and hypopigmentation. Fifty-six percent exhibited extracutaneous manifestations. The presence of extracutaneous manifestations in each subgroup varied: 32% in patients with hyperpigmentation, 73% in patients with hypopigmentation, and 83% in patients with combined hyperpigmentation and hypopigmentation. Cytogenetic analyses were performed in 40% of the patients: peripheral blood lymphocytes were analysed in 48%, skin fibroblasts in 5%, and both analyses were performed in 40%. In the remaining 7% the analysed cell type was not specified. Forty-two percent of the tested patients exhibited an abnormal karyotype; 84% of those presented a mosaic state and 16% presented a non-mosaic structural or numerical abnormality. In patients with extracutaneous manifestations, 43% of the cytogenetically tested patients exhibited an abnormal karyotype. In patients without extracutaneous manifestations, 32% of the cytogenetically tested patients exhibited an abnormal karyotype.

**Conclusion:**

We recommend a uniform parlance when describing the clinical picture of pigmentary mosaicism. Based on the results found in this review, we recommend that patients with pigmentary mosaicism undergo physical examination, highlighting with Wood’s light, and karyotyping from peripheral blood lymphocytes and skin fibroblasts. It is important that both patients with and without extracutaneous manifestations are tested cytogenetically, as the frequency of abnormal karyotype in the two groups seems comparable. According to the results only a minor part of patients, especially those without extracutaneous manifestations, are tested today reflecting a need for change in clinical practice.

**Electronic supplementary material:**

The online version of this article (10.1186/s13023-018-0778-6) contains supplementary material, which is available to authorized users.

## Background

Segmental pigmentary disorders have been reported since the 1960’s, and the idea that they represent genetic mosaicism has since then gradually gained acceptance within dermatology [1–3]. Mosaicism refers to the occurrence of two or more cell populations with different expression of one or more genes, although derived from a single zygote [4].

Two fundamentally different mechanisms divide mosaic skin disorders into two categories: genomic and epigenetic mosaicism [5]. The Lyon-effect, naturally occurring in females, is an example of epigenetic mosaicism, as the random X-inactivation results in differences in the phenotypic expression, even though the genetic makeup in all cells is identical. By contrast, genomic mosaicism is the presence of at least two populations of cells, which differ in the genome. Mosaicism implies the coexistence of a normal and one or more abnormal components, and opposed to this, a few individuals with segmental dyspigmentation have been shown to be chimeric, i.e. consist of cell lines with different but completely normal genome.

**Fig. 1:**
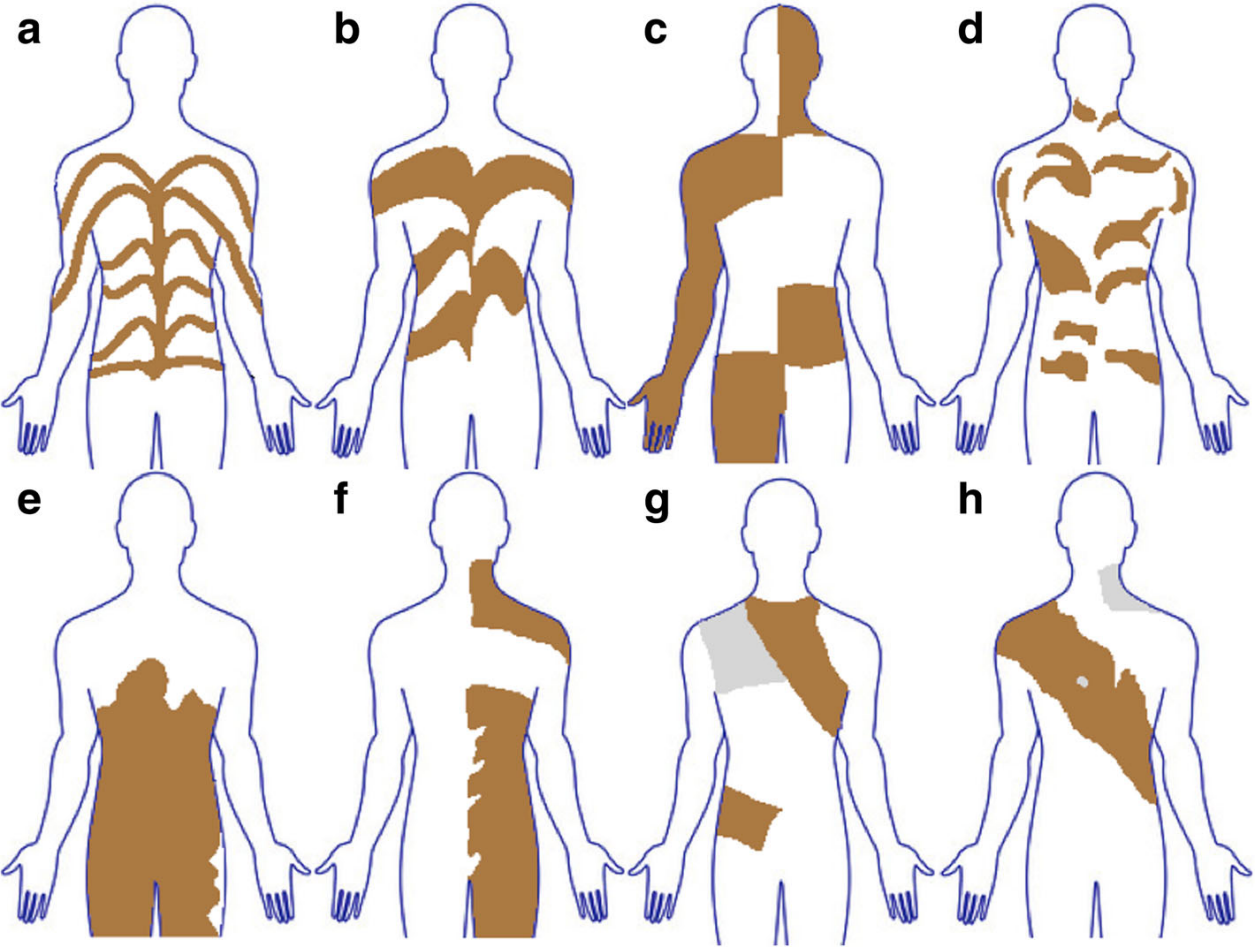
Archetypical patterns of cutaneous mosaicism. (**a**) Type 1a, (**b**) type 1b, (**c**) type 2, (**d**) type 3, (**e**) type 4, (**f**) type 5, (**g**) type 6 seen from the front, (**h**) type 6 seen from the back

**Fig. 2:**
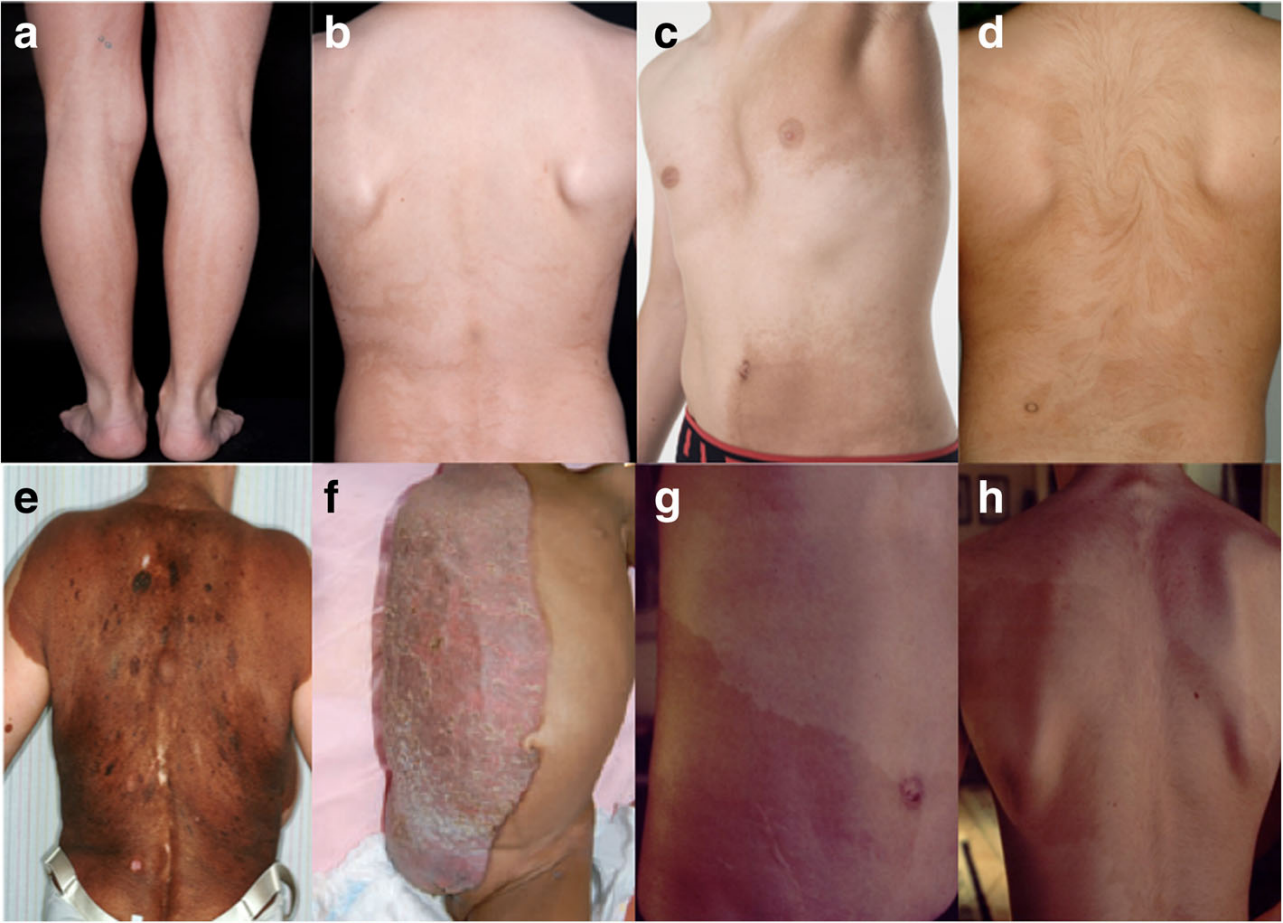
Clinical examples of archetypical patterns of cutaneous mosaicism. (**a**) Hyperpigmentation following Blaschko lines in narrow bands, (**b**) hyperpigmentation following Blaschko lines in broad bands, (**c**) checkerboard pattern, (**d**) phylloid hyperpigmentation, (**e**) giant melanocytic nevus representing patchy pattern, (**f**) CHILD-syndrome representing lateralization pattern [95] (Reprinted with permission from © Wiley Periodicals, Inc.), (**g**) and (**h**) cutis tricolor of the Ruggieri-Happle type (Kindly provided by M. Ruggieri, Catania, Italy)

In 1901 the German dermatologist Alfred Blaschko published an atlas in which he described the lines of various linear skin diseases [6]. The nowadays well-known lines of Blaschko form streaks displaying a V-shape or fountain-like pattern on the back, an S-shape or whorled pattern over the lateral aspects of the trunk, and a linear pattern on the extremities. The lines were not based on anatomic landmarks, but were derived by mapping onto a doll the patterns observed in 83 patients with nevoid abnormalities and 63 patients with acquired linear skin disease. The pattern in the skin is not related to nerve, blood vessel, or lymphatic vessel distribution, but is believed to originate from movement of cells during embryonic life [5, 7]. For heuristic purposes, the Blaschkoid distribution is divided into two subgroups: pigmentation in either narrow or broad bands [5]. Apart from these, five cutaneous patterns in which pigmentary mosaicism may manifest are generally accepted. The seven archetypical patterns are described in the following (Figs. 1 and 2):

- Type 1a: Narrow bands, e.g. observed in hypomelanosis of Ito.
- Type 1b: Broad bands, e.g. observed in McCune Albright syndrome.
- Type 2: Checkerboard pattern (also called flag-like) characterized by alternating squares of hyperpigmentation with a sharp midline separation.
- Type 3: Phylloid pattern with leaf-like or oblong macules showing a dorsal and ventral midline separation.
- Type 4: Patchy pattern without midline separation, e.g. observed in cases of giant melanocytic nevi that do not respect the midline.
- Type 5: Lateralization pattern often observed in CHILD syndrome.
- Type 6: Sash-like pattern.

Streaks of hypopigmentation or hyperpigmentation along the lines of Blaschko have been named hypomelanosis of Ito (HI) and linear and whorled nevoid hypermelanosis (LWNH) [2]. Both HI and LWNH encompass heterogeneous groups of disorders characterized by conditions with hypopigmented or hyperpigmented skin respectively [5]. Unfortunately, multiple terms are used to describe conditions with pigmentary mosaicism, and to avoid confusion, we suggest using the most accurate term, namely, pigmentary mosaicism.

The intent of this literature review was to develop a summary of the current original literature dealing with the subject pigmentary mosaicism for the purpose of optimizing the prospective handling of patients with this condition.

## Methods

The study is a literature review comprising original studies of patients with pigmentary mosaicism. All papers originated from a database search on PubMed with the purpose of identifying all patients with pigmentary mosaicism reported in the literature since 1985. The database was searched using the following keywords, either by themselves or in different combinations: pigmentary mosaicism, mosaicism, hypopigmentation, hyperpigmentation, Blaschko, linear and whorled nevoid hypermelanosis, hypomelanosis of Ito, incontinentia pigmenti achromians, nevus/naevus depigmentosus, achromic nevus/naevus, nevus/naevus achromicus, SegPD, segmental pigmentation disorder, and cutis tricolor. We applied filters to restrict the results to articles with the following criteria: full texts based on case reports or reviews written in English between 01.01.1985 and 01.04.2017. Furthermore, we searched the reference lists of identified articles for additional sources. In total, this led to the identification of 419 articles of which 174 were original papers and included a total number of 651 cases of pigmentary mosaicism.

For each patient the following data were registered: sex, age at publication, age at onset, pigmentation type (hyperpigmentation, hypopigmentation or combination), pigmentation pattern, and family history (Additional file 1).

The age at onset was recorded by group-classification as follows: presentation at birth, within 6 weeks, within the first year, by the age of 2 years, and after the age of 2 years. Furthermore, available information about extracutaneous manifestations, histopathology, and cytogenetic analyses were collected.

*P*-values were calculated using chi-squared test and StataCorp.

## Results

### Cases

Among a total of 651 published patients, 349 (54%) were female and 302 (46%) were male. Two-hundred-and-seventy-eight patients (43%) exhibited hyperpigmentation, 324 (50%) exhibited hypopigmentation, and 48 (7%) exhibited a combination of hyperpigmentation and hypopigmentation. Pigmentation type was not classified in 1 patient.

The abnormal skin pigmentation occurred on all parts of the body. Five-hundred-and-sixteen patients (79%) exhibited a Blaschkoid distribution of the dyspigmentation. Thirty-six patients (6%) exhibited other patterns: 19 patients exhibited a phylloid pattern (53%), 12 patients exhibited a patchy pattern (33%), 3 patients exhibited a checkerboard pattern (8%), and 2 patients exhibited a sash-like pattern (6%). Two patients exhibited a combination of Blaschkoid and phylloid pattern. The distribution of the dyspigmentation was not reported in 101 patients (15%).

Age at onset of the abnormal pigmentation was reported in 378 patients (58%). In 282 patients (75%) the abnormal pigmentation was noted early (Table 1): 174 at birth, 25 within 6 weeks, and 83 within the first year. Later observation occurred in 95 patients (25%): Fifty-seven by the age of 2 years and 38 after the age of 2 years.

**Table 1:** Age at onset for the different pigmentation types in 650 patients with a classified phenotype

	Hyperpigmentation	Hypopigmentation	Hyper- and hypopigmentation
At birth	51	106	17
Within 6 weeks	12	12	1
6 weeks – 1 year	34	42	7
1 year – 2 years	9	46	2
After the age of 2 years	15	18	5
NA	157	100	16
Total	278	324	48

A family history of pigmentary mosaicism occurred in 25 patients (4%), while 342 patients (52%) were characterized as sporadic cases. No data on family history were given in 284 cases (44%).

### Extracutaneous manifestations

Extracutaneous manifestations were described in 367 patients (56%). The distribution of extracutaneous manifestations on each subgroup was 89 patients (32%) with hyperpigmentation, 238 patients (73%) with hypopigmentation and 40 patients (83%) with both hyperpigmentation and hypopigmentation (Table 2). In 1 patient without extracutaneous manifestations, the pigmentation type was not reported. Omitting the patient with unreported/unknown pigmentation type, the difference in the distribution of extracutaneous manifestations in the three groups was found to be statistically significant (*p* < 0.001).

**Table 2:** Distribution of extracutaneous manifestations in patients with hyperpigmentation, hypopigmentation, or both

	With extracutaneous manifestations	Without extracutaneous manifestations	Total
Hyperpigmentation	89	189	278
Hypopigmentation	238	86	324
Hyper- and hypopigmentation	40	8	48
NA	0	1	1
Total	367	284	651

The most frequently reported abnormalities were developmental delay, skeletal deformities, seizures, dysmorphic facial features, and psychomotor retardation (Additional file 2).

Developmental delay was described in 198 patients (54% of patients with extracutaneous manifestations). Skeletal deformities including scoliosis, clinodactyly, delayed bone age, overlapping toes, and split hand malformation were described in 140 patients (38%). Seizures, epilepsy or abnormal EEG were described in 137 patients (37%), and dysmorphic facial features such as depressed nasal bridge, hypertelorism, epicanthus, and low set ears were described in 114 patients (31%). Psychomotor retardation was described in 57 patients (16%) (Table 3). In addition to the abovementioned, a wide range of other abnormalities was described, including behavioural disturbances, hearing loss, and defects in heart, kidney, and brain.

**Table 3:** Distribution of the most frequently reported extracutaneous manifestations in 367 patients (56%)

Developmental delay	198
Skeletal deformities	140
Seizures, epilepsy and/or abnormal EEG	137
Dysmorphic facial features	114
Psychomotor retardation	57

### Cytogenetic analyses

Cytogenetic analyses were performed in 263 patients (40%). Peripheral blood lymphocytes were analysed in 127 patients (48%), skin fibroblasts in 13 patients (5%), both analyses were performed in 106 patients (40%), and in 17 patients (7%) the analysed cell type was not specified (Table 4). An abnormal result of the cytogenetic analysis was found in 111 patients (42% of tested patients): 93 presented as mosaics, while 18 had non-mosaic chromosomal abnormalities detected.

**Table 4:** Results of cytogenetic analyses (performed in 263 of 651 patients, 40%)

	Normal	Abnormal	Total
Peripheral blood lymphocytes	103	24	127
Skin fibroblasts	5	8	13
Blood lymphocytes and skin fibroblasts	33	73	106
Unknown cell type analysed	11	6	17
Total	152	111	263

**Fig. 3:**
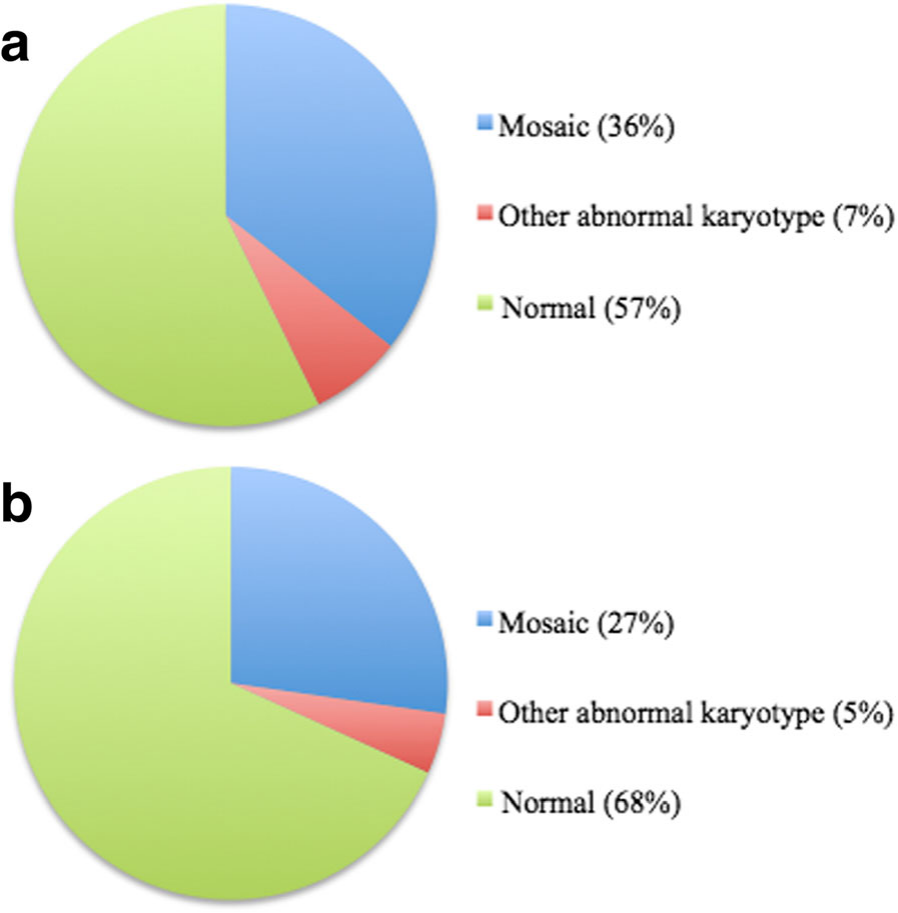
Outcome of cytogenetic analysis. (**a**) The outcome of cytogenetic analysis performed in 241 patients with extracutaneous manifestations and (**b**) the outcome of cytogenetic analysis performed in 22 patients without extracutaneous manifestations

In 367 patients with extracutaneous manifestations, karyotyping was performed in 241 (66%), and an abnormal karyotype (mosaic state, structural abnormality or numerical abnormality) was found in 104 (43%) of those. In 284 patients without extracutaneous manifestations, karyotyping was performed in 22 (8%), and an abnormal karyotype was found in 7 (32%) of those (Table 5, Fig. 3). No significant difference in the distribution of chromosomal abnormalities was detected in patients with and without extracutaneous manifestations (*p* = 0.320).

**Table 5:** Chromosomal findings in 367 patients with extracutaneous manifestations and 284 patients without extracutaneous manifestations

	With extracutaneous manifestations	Without extracutaneous manifestations	Total
Genetic mosaic	87	6	93
Non-mosaic	17	1	18
Normal	137	15	152
Analysis not performed	126	262	388
Total	367	284	651

Of the 263 patients who had a cytogenetic analysis performed, an abnormal result was found in 111 patients (42%). Ninety-three patients (84%) exhibited a mosaic state (1 monogenic), and the remaining 18 patients (16%) exhibited non-mosaic chromosomal abnormalities; 13 had structural anomalies [8–17], 4 had numerical anomalies [18–20], and 1 abnormal karyotype was not described further [21].

The chromosomes involved in pigmentary mosaicism were the following: 2 [22, 23], 3 [24–26], 4 [27–30], 5 [31, 32], 7 [33–42], 8 [43], 9 [29, 44, 45], 10 [46], 12 [16, 47, 48], 13 [4, 16, 17, 49–57], 14 [30, 54, 58], 15 [59–61], 16 [62], 18 [16, 30, 43, 63–66], 20 [67–71], 22 [43, 72], and the sex chromosomes [15, 16, 30, 73–80]. Furthermore, mosaic states with translocations between chromosomes 1, 6, 12, 13, 14 and 21 were found [81–83].

### Histopathology

Skin biopsies for histopathologic examination were performed in 238 patients (37%). The biopsies showed an increase of melanin in the hyperpigmented areas and a decrease of melanin in the hypopigmented areas, compared to normal skin. In addition, routine transmission electron microscopy (TEM) on the skin biopsies was performed in 18 patients (Additional file 3).

## Discussion

To define the spectrum of pigmentary mosaicism more accurately and to map the correlation with extracutaneous manifestations, we retrospectively reviewed literature detailing 651 patients with pigmentary mosaicism. Importantly, we found that the clinical picture of pigmentary mosaicism and the medical terms used to describe it are variable. In the literature, including recent articles, a wide range of terms, e.g. LWNH, HI, incontinentia pigmenti achromians, nevus depigmentosus, achromic nevus, and segmental pigmentation disorder are used, and these multiple terms foster unnecessary confusion and ambiguity, as they in fact all describe the same clinically heterogeneous condition. Therefore, as Happle et al. states, these expressions should be avoided since they erroneously indicate nosological entities [5]. Adding to further confusion, the definitions of these terms seem to vary depending on the author. Patients with coexisting hyper- and hypopigmented skin may be referred to as cutis tricolor, a term introduced by Happle et al. [84]. Lipsker et al. suggested the term cutis bicolor in patients with two shades of colour [73], but as this term does not indicate the type of pigmentation anomaly, we recommend using the more specific terms hyperpigmentation and hypopigmentation.

It is often difficult or even impossible to determine, whether the light or the dark skin component is the pathological part, especially in children [5, 67]. Based on successful applications in the literature, we recommend using Wood’s light to highlight the pigmentation anomalies [67, 85].

### Cases

The gender composition in our study was almost equal: 46% were male and 54% were female. Similar results have been observed in other studies [86].

Forty-three percent exhibited hyperpigmentation, 50% exhibited hypopigmentation, and 7% exhibited a combination of hyper- and hypopigmentation. The low percentage of patients exhibiting both hyper- and hypopigmentation was expected, as this requires three pigmentarily different cell lines, as opposed to only two in patients exhibiting either hyper- or hypopigmentation.

The distribution of the dyspigmentation was primarily Blaschkoid (79%) and occurred on all parts of the body. In practice, the patterns can be difficult to distinguish, as reported by Hansen et al. [32]. We must keep in mind that different authors might classify a given pigmentation pattern differently, and based on this, biased results are possible. In most cases however, the pigmentation pattern is clear and the classification is straightforward. Furthermore, in doubtful cases it was possible to verify the classification by the clinical photos provided in most articles.

Our study showed that the abnormal pigmentation was noted within the first year of life in 282 (75%) of the patients. However, this percentage might not be accurate, as it can be difficult to determine the exact time of presentation of the dyspigmentation, e.g. in very light skinned Caucasians where a hypopigmented anomaly can go unnoticed in the first years of life. Additionally, in many studies the dermatologists saw the patients later in life, where parents reported the age at onset based on their memory.

Most frequently, pigmentary mosaicism appears sporadically, but a family history of the condition was described in 4% of the reported cases. A rare case of two paternal half-brothers with pigmentary mosaicism of the hyperpigmented type was reported [26]. A chromosomal mosaicism with a partial duplication of chromosome 3p was demonstrated in two different tissues from one patient, whereas the lymphocytes of the other patient did not show the chromosomal anomaly and therefore no common cause for the pigmentary mosaicism was found. A family with LWNH in three successive generations has also been reported: a 12-year-old girl, the 45-year-old mother, and the 65-year-old grandmother [19]. Only chromosome analysis was performed and here again, no possible common genetic cause was identified.

### Extracutaneous manifestations

In this review a strikingly high frequency of extracutaneous manifestations (55%) was seen in comparison with other individual reviews, where extracutaneous manifestations were reported in 0, 8 and 30% of the patients [87–89]. Our review revealed a presence of extracutaneous manifestations in 32% of patients with hyperpigmentation, 73% of patients with hypopigmentation, and 83% of patients with combined hyperpigmentation and hypopigmentation. These results differ from findings in previous reviews, where hypopigmentation and hyperpigmentation are found to be associated with extracutaneous manifestations to an equal extent [87, 90].

The most frequently reported extracutaneous anomalies were skeletal deformities, seizures, mental retardation, dysmorphic facial features, and developmental delay. Regarding these features it is important to bear in mind that the groups of different anomalies should be considered heterogeneous collections of disorders indicating numerous different underlying mosaic states, and not distinct groups of pheno- or genotypes [5].

### Cytogenetic analyses

In this review, cytogenetic analysis was performed in 66% (241 patients) of patients with extracutaneous manifestations and by comparison in only 8% (22 patients) of patients without extracutaneous manifestations. Thirty-two percent of patients without extracutaneous manifestations and 43% of patients with extracutaneous manifestations were found to have abnormal karyotypes. On the basis of the available data, it is not possible to detect significant differences in the presence of chromosomal abnormalities in the two groups (*p* = 0.320). The remarkable difference in proportion of cytogenetically tested patients depicts a need to change daily practice when handling patients with suspected pigmentary mosaicism. It is also important to test patients without extracutaneous manifestations, as the frequency of abnormal karyotype seems comparable to patients with extracutaneous manifestations. The data represent procedures performed over a period of more than 30 years, and the relevance of the results to the recent dermatologic community can therefore be questioned. However, the tendency to test primarily when extracutaneous manifestations are observed is also seen in more recent studies, which confirms the need for standardization of future handling.

Sixteen percent of the cytogenetically tested patients exhibited non-mosaic structural or numerical abnormalities, and in most of these cases the detected aberration does not in itself explain the pigmentary mosaic. However, in six cases a chromosomal translocation involving chromosome X was seen, and differences in gene expression after X-inactivation of either the normal or the translocated X may explain the pigmentary mosaicism.

The chromosomes involved in the investigated cases of pigmentary mosaicism were: 2, 3, 4, 5, 7, 9, 10, 12, 13, 14, 15, 16, 18, 20, 21, 22, and the sex chromosomes. The wide range of this result confirms the earlier mentioned and important point, that the etiology of pigmentary mosaicism is heterogeneous and complex and should not be considered distinct syndromes despite similarities in the clinical picture.

Based on the literature review, we recommend that fibroblasts obtained from light and dark skin are analysed as well as peripheral blood lymphocytes. Despite following this procedure, failure to detect a chromosomal mosaicism may persist, if the mosaic state is present in neither lymphocytes nor fibroblasts. Taibjee et al. studied 10 patients with pigmentary mosaicism in whom previous karyotyping was negative, and were only able to show cytogenetic abnormalities in keratinocytes in 1 of them [67]. Apart from the abovementioned reason, another explanation is that the genetic change responsible for the pigmentary mosaicism cannot be visualized at the chromosomal level, but may be anything from a point mutation to a copy number variation too small to detect by standard chromosome analysis [91]. Finally, the differences in pigmentation may be caused by epigenetic mechanisms.

For therapeutic reasons, it is important to differentiate pigmentary mosaicism from other diagnoses with abnormal pigmentary findings such as incontinentia pigmenti, McCune-Albright syndrome, vitiligo, neurofibromatosis Recklinghausen, piebaldism, and tuberous sclerosis. This, in addition to earlier mentioned reasons, reinforces our recommendation to perform cytogenetic evaluation of peripheral blood lymphocytes and skin fibroblasts in all patients with suspected pigmentary mosaicism. When performing chromosome analysis, a significant number of metaphases from both normal and affected skin should be investigated to ensure detection of even small percentages of abnormal cells and to detect possible cytogenetic variations between the two differently coloured skin types. Though more expensive, a contemporary approach is to perform chromosome micro array analysis on uncultured cells [4]. When cell culturing is avoided, the selection bias, which is usually in favour of the normal clone, is minimized [92]. Another strength of this method is the capacity to detect even small deletions or duplications, which cannot be detected by standard chromosome analysis [93]. On the other hand only abnormal clones of a certain size (usually above 5–10%) can be detected by chromosome micro array. The two approaches have different advantages and may be combined to improve the detection rate of cytogenetic abnormalities in cases of pigmentary mosaicism. Finally, next-generation sequencing (NGS) of the exome or even the genome may be added as the method of choice in the near future [94], as it gives the ability to detect monogenic causes even in a mosaic state. When further improved, the NGS techniques may even replace chromosome micro array and banding techniques for detection of copy number variations and chromosomal rearrangements.

## Conclusion

The intent of this literature review was to compile information on previous cases of pigmentary mosaicism for the purpose of optimizing the prospective handling of patients with this condition. We have examined 174 papers presenting 651 patients with pigmentary mosaicism, and found that the terms used when describing the clinical picture of pigmentary mosaicism are diverse. The many names give a fallacious impression of different subgroups and to avoid confusion, we therefore strongly recommend that future accounts use the terms hyper- and hypopigmentation and as an umbrella term: pigmentary mosaicism.

We confirmed that extracutaneous manifestations typically involve the central nervous system and musculoskeletal systems, and found that the frequency of these manifestations was higher than reported in the smaller series of cases included in the review. Furthermore, we found a statistically significant difference in the distribution of extracutaneous manifestations in the three pigmentation types. Finally and essentially, we can conclude that it is important to test both patients with and without extracutaneous manifestations cytogenetically, since no statistically significant difference in frequency of karyotype anomalies in the two groups was found.

An apparent difference in the proportion of cytogenetic tests performed in patients with and without extracutaneous manifestations poses a need for consensus and hence changes in the practical handling of patients with pigmentary mosaicism. Therefore, based on the results of the literature review, we propose the following guideline for classification and handling of all patients with suspected pigmentary mosaicism:

1. Physical examination of the skin.
2. Highlight the pigmentation with Wood’s light.
3. Systematic clinical examination.
4. Standard chromosome analysis of a large number of metaphases or preferable chromosome micro array of uncultured cells. Exome sequencing may be an increasingly available alternative.
5. Exclude differential diagnoses.

## Additional files

Additional file 1: Case overview. Sex, ethnicity, age at publication, age at onset, pigmentation type, Blaschkoid distribution or other patterns, distribution of pigmentary lesions and family history. (DOC 499 kb)

Additional file 2: Extracutaneous manifestations. (DOCX 170 kb)

Additional file 3: Diagnostic methods and karyotype. Karyotype, results of skin biopsies and cytogenetic analyses of skin fibroblasts and peripheral blood lymphocytes. (DOCX 114 kb)

Additional file 4: Supplemental bibliography. (DOCX 52 kb)

## Abbreviations

CGH: Comparative genomic hybridization
CHILD syndrome: Congenital hemidysplasia with ichthyosiform nevus and limb defects
HI: Hypomelanosis of Ito
LWNH: Linear and whorled nevoid hypermelanosis
NGS: Next-generation sequencing
SegPD: Segmental pigmentation disorder
TEM: Transmission electron microscopy
