# Supplementary material for: Pigmentary mosaicism: a review of original literature and recommendations for future handling
Source: Orphanet J Rare Dis. 2018 Mar 5;13:39. doi: 10.1186/s13023-018-0778-6 (PMC5839061; doi:10.1186/s13023-018-0778-6)
Supplement: Supplementary file 1 — Case overview. Sex, ethnicity, age at publication, age at onset, pigmentation type, Blaschkoid distribution or other patterns, distribution of pigmentary lesions and family history. (DOC 499 kb) [file 13023_2018_778_MOESM1_ESM.doc]

Additional file 1: Case overview

| **References** | **Case number** | **Sex**  **Female (F) Male (M)** | **Age at publication /**  **Age at onset** | **Blaschkoid distribution** | **Other patterns** | **Distribution of pigmentary lesions** | **Family history**  **Yes (Y) No (N)** |
| --- | --- | --- | --- | --- | --- | --- | --- |
| Afsar et al. 2007 | 1 | F | 12 years/At birth |  |  | Hyperpigmented macule with hypertrichosis and hypopigmented macules on right thigh and gluteal area. | N |
| Aguayo-Leiva et al. 2011 | 2 | F | 2 months/At birth |  | Checkerboard pattern | Erythema and hyperpigmentation on trunk and extremities. | N |
| Akahoshi et al. 2004 | 3 | M | 10 years/- | + |  | Hyperpigmentation on trunk and lower extremities. | N |
| Akiyama et al. 1994 | 4 | F | 3 months/3 weeks after birth | + |  | Hyperpigmentation on trunk and extremities. | Y (mother) |
|  | 5 | F | 33 years/Several weeks after birth | + |  | Hyperpigmentation on trunk and extremities. | N |
| Al Aboud et al. 2005 | 6 | F | 17 years/At birth | + |  | Hyperpigmentation on trunk and extremities. | Y (sister) |
|  | 7 | F | 15 years/At birth | + |  | Hyperpigmentation on trunk and extremities. | Y (sister) |
| Alrobaee et al. 2004 | 8 | M | 2 years/Few months after birth | + |  | Hyperpigmentation on trunk and extremities. | N |
| Alvarez et al. 1993 | 9 | M | 13 years/1 year | + |  | Hyperpigmentation on trunk and proximal extremities. | N |
| Baba et al. 2002 | 10 | F | 18 years/At birth | + |  | Hyper- and hypopigmentation on neck and left upper extremity. | NA |
| Baba et al. 2003 | 11 | F | 15 years/At birth | + |  | Hyper- and hypopigmentation on neck and trunk. | Y (sister) |
|  | 12 | F | 12 years/At birth | + |  | Hyper- and hypopigmentation on right side of the back and adjacent abdomen with a sharp demarcation at the anterior midline. | Y (sister) |
| Ballmer-Weber et al. 1996 | 13 | F | 16 months/11 months | + |  | Hypopigmentation on trunk and extremities. | N |
| Bartholomew et al. 1987 | 14 | F | 16 months/Within the first week |  |  | Hypopigmentation on the trunk and lower extremities. | NA |
| Baty et al. 2001 | 15 | F | 9 years/- | + |  | Hypopigmentation on extremities, predominant on the left. | N |
|  | 16 | F | 8 years/- | + |  | Hypopigmentation on trunk and extremities. | N |
| Bocian et al. 1993 | 17 | F | 8 months/At birth | + |  | Hyper- and hypopigmentation on trunk and buttocks. | N |
| Boente et al. 2011 | 18 | F | 3 years/- |  |  | Hyper- and hypopigmented macules on trunk and external genital area. | N |
|  | 19 | F | 15 months/- |  |  | Hyper- and hypopigmented macules on trunk and extremities. | N |
| Boon et al. 1996 | 20 | F | 6 months/At birth | + |  | Hypopigmentation on extremities and midline of abdomen. | NA |
| Brar et al. 2008 | 21 | F | 4.5 years/6 weeks | + |  | Hyperpigmentation overall. | NA |
| Brock et al. 2012 | 22 | M | 11 years/3 years |  |  | Hyperpigmented streaks on right chest, right arm, and left leg. | N |
| Bygum et al. 2011 | 23 | F | 17 years/4 months | + |  | Hyperpigmentation with papillomatosis on scalp, face, neck and trunk. Sharp demarcation at the anterior midline. | NA |
| Bygum et al. 2012 | 24 | M | 3 years/At birth |  | Phylloid pattern | Hyperpigmentation on trunk and extremities. | NA |
| Capaldi et al. 2005 | 25 | F | 6 years/2 years | + |  | Hypopigmentation on trunk and proximal extremities. | NA |
|  | 26 | F | 15 years/Infancy | + |  | Hypopigmentation on extremities. | NA |
| Cappanera et al. 2011 | 27 | F | 12 years/- | + |  | Hypopigmentation on left side of the trunk and extremities. | NA |
| Castori et al. 2012 | 28 | M | 8 years/- | + |  | Hyperpigmentation on trunk and right upper extremity. Midline demarcation at neck. | NA |
| Cellini et al. 1998 | 29 | M | 7 years/2-3 years | + |  | Hypopigmentation on back and extremities. | N |
| Chitayat et al. 1990 | 30 | M | 14 months/- | + |  | Hypopigmentation on trunk and lower extremities. | N |
| Cho et al. 2011 | 31 | M | 9 years/At birth |  | Patchy pattern | Hyperpigmented patch on upper back, hypopigmented macules within. | N |
| Cho et al. 2012 | 32-61 | 14 F  16 M | 30 years (median age, 4 - 56 years)/  14.27 years (mean, birth – 54 years) | + 30/30 |  | 30/30 hyperpigmentation.  19/30 trunk, 13/30 upper extremity, 11/30 back, 10/30 chest, 9/30 lower extremity, 8/30 abdomen, 5/30 face and neck.  15/30 right side, 11/30 left side, 4/30 both sides. | Y (1/30 mother)  N (29/30) |
| Choi et al. 2005 | 62 | M | 16 years/15 years |  |  | Hyperpigmentation on left abdomen and back. | N |
| Cohen et al. 2014 | 63 | M | 26 months/At birth | + |  | Hypopigmentation on back, buttock, and posterior thigh. | NA |
|  | 64 | M | 4 years/At birth | + |  | Hypopigmentation on right abdomen and low back. | NA |
|  | 65 | F | 11 months/At birth | + |  | Diffuse hypopigmentation. | NA |
|  | 66 | F | 5 months/At birth | + |  | Hypopigmentation on left abdomen and groin. | NA |
|  | 67 | M | 4 months/At birth | + |  | Hyperpigmentation on right flank, groin, thigh, and lower extremity. | NA |
|  | 68 | M | 19 months/At birth | + |  | Hyperpigmentation on bilateral lower extremities. | NA |
|  | 69 | F | 4 months/At birth | + |  | Hyperpigmentation on left upper extremity. | NA |
|  | 70 | F | 2 years/At birth | + |  | Diffuse hyperpigmentation. | NA |
|  | 71 | F | 3 years/At birth | + |  | Hyperpigmentation on posterior thighs and lower extremities. | NA |
|  | 72 | F | 8 years/At birth | + |  | Hypopigmentation on left lateral neck, shoulder, and upper extremity. | NA |
|  | 73 | F | 8 months/At birth | + |  | Diffuse hyperpigmentation. | NA |
|  | 74 | F | 1 year/At birth | + |  | Hyperpigmentation on the trunk. | NA |
|  | 75 | M | 6 months/At birth | + |  | Hypopigmentation on right upper chest, abdomen, and suprapubic area. | NA |
|  | 76 | M | 5 years/At birth | + |  | Diffuse hyperpigmentation. | NA |
|  | 77 | M | 15 years/At birth | + |  | Diffuse hyperpigmentation. | NA |
|  | 78 | M | 9 months/At birth | + |  | Hypopigmentation on left lower chest, abdomen, and back. | NA |
|  | 79 | F | 3 months/At birth | + |  | Hyperpigmentation on left jawline and preauricular region. | NA |
|  | 80 | F | 22 months/At birth | + |  | Hypopigmentation on right lower extremity. | NA |
|  | 81 | M | 11 months/2 weeks | + |  | Hypopigmentation on right abdomen and face. | NA |
|  | 82 | F | 3.5 years/1 month | + |  | Hypopigmentation on right abdomen and back. | NA |
|  | 83 | F | 7 months/1 month | + |  | Hypopigmentation on anterior trunk and left breast. | NA |
|  | 84 | M | 13 months/2 months | + |  | Hyperpigmentation on left upper back, abdomen, and groin. | NA |
|  | 85 | F | 5 months/2 months | + |  | Hypopigmentation on right abdomen and flank. | NA |
|  | 86 | F | 22 months/4 months | + |  | Hyperpigmentation on left upper chest, shoulder, and upper extremities. | NA |
|  | 87 | F | 7 months/5 months | + |  | Hyperpigmentation on trunk and extremities. | NA |
|  | 88 | F | 2 years/5 months | + |  | Hypopigmentation on bilateral flexure surfaces of upper and lower extremities. | NA |
|  | 89 | F | 18 months/6 months | + |  | Hypopigmentation on left lower extremity. | NA |
|  | 90 | F | 13 months/9 months | + |  | Hypopigmentation on left back. | NA |
|  | 91 | F | 2 years/10 months | + |  | Hyperpigmentation on bilateral forearms and left lower extremity. | NA |
|  | 92 | M | 14 months/11 months | + |  | Diffuse hypopigmentation. | NA |
|  | 93 | F | 16 months/12 months | + |  | Hyperpigmentation on bilateral thighs and knees. | NA |
|  | 94 | F | 3.5 years/24 months | + |  | Hypopigmentation on right abdomen and back. | NA |
|  | 95 | F | 3 years/24 months | + |  | Hypopigmentation on right abdomen and flank. | NA |
|  | 96 | M | 7 years/5 years | + |  | Hypopigmentation on right lower extremity. | NA |
|  | 97 | F | 9 years/6 years | + |  | Hypopigmentation on left chest and upper extremity. | NA |
|  | 98 | M | 13 years/12 years | + |  | Hypopigmentation on right upper extremity. | NA |
| Correa-Cerro et al. 1997 | 99 | F | -/At birth | + |  | Hypopigmentation on trunk and extremities. | N |
| Delaporte et al. 1996 | 100 | M | 3 years/6 weeks | + |  | Hyperpigmentation on trunk and thighs. | N |
| Desai et al. 1988 | 101 | M | 3 years/At birth | **+** |  | Hypopigmentation on right side of the trunk, right genital region, right extremities, left chest, and left upper extremity. | N |
| Devillers et al. 2011 | 102 | M | 12 months/2 months | + |  | Hypopigmentation on abdomen, back, and extremities. | NA |
| Dhar et al. 2009 | 103 | F | 3 years/- |  | Phylloid pattern | Hypopigmentation on trunk and extremities. | NA |
| Di Lernia 2007 | 104 | F | 7 years/At birth | **+** |  | Hyperpigmentation on right side of the trunk. | N |
|  | 105 | F | 1 year/1 month | + |  | Hyperpigmentation on right buttock and thigh. | N |
|  | 106 | M | 2 years/1 year | + |  | Hyperpigmentation on trunk. | N |
|  | 107 | M | 2 years/At birth | + |  | Diffuse hyperpigmentation. | N |
|  | 108 | F | 1 year/1 year | + |  | Diffuse hyperpigmentation. | N |
|  | 109 | M | 5 years/4 years | + |  | Hyperpigmentation on left face and neck. | N |
|  | 110 | M | 6 years/9 months | + |  | Hyperpigmentation on trunk and upper extremities. | N |
|  | 111 | M | 10 years/4 years | + |  | Hyperpigmentation on trunk and right arm. | N |
|  | 112 | M | 3 years/1 year | + |  | Diffuse hyperpigmentation. | N |
|  | 113 | M | 4 years/3 months | + |  | Diffuse hyperpigmentation. | N |
|  | 114 | M | 10 years/5 years | + |  | Hyperpigmentation on right trunk. | N |
|  | 115 | F | 5 years/At birth | + |  | Hyperpigmentation on thighs and genitalia. | N |
|  | 116 | F | 1 year/3 months | + |  | Hyperpigmentation on right buttock and flexor sides of the thighs. | N |
|  | 117 | M | 2 years/1 year | + |  | Hyperpigmentation on trunk. | N |
|  | 118 | F | 2 years/2 years | + |  | Hyperpigmentation on trunk. | N |
|  | 119 | M | 3 years/3 years | + |  | Hyperpigmentation on left arm. | N |
| Di Lernia 2015 | 120 | M | 18 years/At birth | + |  | Hypopigmentation on back and extremities. | N |
| Donnai et al. 1988 | 121 | F | 12 years/2 years | + |  | Hypopigmentation on trunk, upper extremities and thighs. Anterior midline demarcation. | N |
|  | 122 | F | 1 year/5 months | + |  | Hypopigmentation on back and lower extremities. | N |
|  | 123 | M | 4 years/17 months | + |  | Hypopigmentation on trunk and lower extremities. | N |
| Dúran-McKinster et al. 2002 | 124 | F | 5 years/ First months of life | + |  | Hypopigmentation on trunk, upper extremities, flexor sides of lower extremities. Sharp midline demarcation. | NA |
| Eid et al. 2013 | 125 | F | 4 years/- |  |  | Hyperpigmented macules on abdomen and right upper extremity. | NA |
| El-Sawy et al. 2011 | 126 | F | 16 months/6 months | + |  | Hypopigmentation on back, upper extremities, and lower extremities. | NA |
| Errichetti et al. 2016 | 127 | F | 17 years/2 months | + |  | Hyperpigmentation on trunk and extremities. | N |
| Ertam et al. 2009 | 128 | F | 11 years/At birth | + |  | Hyperpigmentation on the entire body. | N |
| Faletra et al. 2012 | 129 | M | 3.5 years/At birth |  | Phylloid pattern | Hypopigmentation on trunk and extremities. | NA |
|  | 130 | M | 3 years/- |  | Phylloid pattern | Hypopigmentation on trunk and extremities. | NA |
| Fan et al. 1994 | 131 | F | 4 months/1 week | + |  | Hypopigmented macules on right chest, right upper extremity and right aspect of the scalp. Sharp midline demarcation. | N |
| Finkelstein et al. 1992 | 132 | F | 5 years/1 year | + |  | Hypopigmented macules on the entire body. | N |
| Fleury et al. 1986 | 133 | M | 2 years/At birth |  |  | Hypopigmentation on trunk and extremities. | N |
|  | 134 | M | 1 year/At birth |  |  | Hypopigmentation on posterior part of trunk and on flexor sides of the lower extremities. | Y (mother) |
|  | 135 | F | 13 years/- |  |  | Hypopigmentation on chest. | N |
|  | 136 | F | 2 years/- |  |  | Hypopigmentation on trunk, left upper extremity and lower extremities. | NA |
| Fogu et al. 2008 | 137 | F | 12 years/1 year | + | Phylloid pattern | Hypopigmentation on back and lower extremities. | N |
| Fritz et al. 1998 | 138 | F | -/1 year | + |  | Hyper- and hypopigmentation on trunk and upper extremities. | NA |
| Fujimoto et al. 1985 | 139 | F | 2 years and 3 months/1 month |  |  | Hyperpigmentation on trunk and extremities. | NA |
| Fujino et al. 1995 | 140 | F | - /At birth |  |  | Hypopigmentation on left trunk and left lower extremity. | NA |
| Garcia Muret et al. 2002 | 141 | M | - /8 months | + |  | Hypopigmentation on left trunk, left upper extremity, and left lower extremity. | NA |
| George et al. 1992 | 142 | F | 15 years/3 months |  |  | Hypopigmentation on trunk and extremities. | N |
| Gerdes et al. 2006 | 143 | F | 14 years/12 years | + |  | Hypopigmentation on right shoulder, right upper extremity, midline of back, and left lower extremity. | NA |
| Gonzalez-del Angel et al. 2014 | 144 | F | 12 years/17 months | + |  | Hyperpigmented macules with predominance on the left. | NA |
| Gonzalez-Ensenat et al. 2009 | 145 | F | 8 years/First year of life |  | Phylloid pattern | Hypopigmentation on trunk and extremities. | NA |
|  | 146 | F | 15 years/At birth |  | Phylloid pattern | Hypopigmentation on trunk and extremities. | NA |
| Grazia et al. 1993 | 147 | F | 11 years/At birth | + |  | Hypopigmentation on trunk. | N |
| Griebel et al. 1989 | 148 | F | 15 years/6 years | + |  | Hypopigmentation on one leg, trunk, and arms. | NA |
|  | 149 | F | 4 years/5 months | + |  | Hypopigmentation on trunk, right upper extremity and lower extremities. | NA |
|  | 150 | M | 4 years/3 years | + |  | Hypopigmentation on trunk and lower extremities. | N |
|  | 151 | F | 6 years/3 years | + |  | Hypopigmentation on left extremities. | N |
| Gupta et al. 2007 | 152 | F | 1 day old/At birth | + |  | Hypopigmentation on the entire body. | NA |
| Gutte 2014 | 153 | M | 28 years/20 years | + |  | Hyperpigmentation on trunk and right pubic area. | N |
| Hansen et al. 2003 | 154 | F | 5 years/- | + | Phylloid pattern | Hyperpigmentation on trunk and lower extremities with predominance on the left. Sharp midline demarcation. | N |
| Hansen et al. 2010 | 155 | M | 3 years/2 years | + |  | Hypopigmentation on right side of the back and right lower extremity. | NA |
| Happle et al. 1997 | 156 | M | 17 years/- |  |  | Café-au-lait spots on right mandibular area and the right upper extremity. Hypopigmentation on right side of the neck and shoulder. | NA |
| Happle 2009 | 157 | F | 24 years/At birth | + |  | Hyperpigmentation on the entire body. | NA |
|  | 158 | F | -/At birth | + |  | Hyperpigmentation on left side of the body. | Y (siblings and mother) |
|  | 159 | F | -/At birth | + |  | NA | Y (siblings and mother) |
|  | 160 | M | 48 hours/At birth | + |  | Hyperpigmentation on the entire body. | Y (siblings and mother) |
| Happle et al. 2012 | 161 | M | 32 months/Few weeks after birth |  | Phylloid pattern | Hyperpigmented macules on trunk and extremities. Dorsal and ventral midline separation. | N |
|  | 162 | F | 11 years/- |  | Phylloid pattern | Hyperpigmentation on back. | NA |
|  | 163 | F | 12 years/- |  | Phylloid pattern | NA | NA |
|  | 164 | M | 20 years/- |  | Phylloid pattern | NA | NA |
| Hartmann et al. 2004 | 165 | M | 7 years/3 years | + |  | Hyperpigmentation on the entire body. | N |
| Hassab-El-Naby et al. 1996 | 166 | M | 3.5 years/At birth | + |  | Hyperpigmentation on right side of the trunk and right thigh. | N |
| Hernandez-Martin et al. 2014 | 167-191 | 12 F  13 M | 2–24 months (mean 12.2 months)/- |  |  | 25/25 hyperpigmented macules on forehead and temple.  6/25 café-au-lait spots on trunk and extremities. | N |
| Hogeling et al. 2010 | 192-230 | 20 F  19 M | -/3.4 months (mean) | + 3/39 |  | 28/39 chest/abdomen, 20/39 back, 9/39 extremities, 9/39 face, 6/39 neck.  32/39 ventral delineation, 7/39 dorsal delineation. | Y (2/39)  N (37/39) |
| Hong et al. 2008 | 231 | M | 30 years/Infancy | + |  | Hyperpigmentation on anterior trunk and flexor sides of the thighs. | N |
| Horn et al. 1997 | 232 | F | 13 years/- |  | Phylloid pattern | Hypopigmentation on trunk and extremities. | N |
| Horn et al. 2002 | 233 | M | 15 years/After first year of life | + |  | Hyperpigmentation on trunk and extremities. | Y (half-brother) |
|  | 234 | M | 9 years/During first month of life | + |  | Hyperpigmentation on trunk and extremities. Sharp midline demarcation. | Y (half-brother) |
| Ishikawa et al. 1985 | 235 | M | 14 years/3 months |  |  | Hypopigmentation on back and extremities. | NA |
| Jagia et al. 2004 | 236 | F | 9 years/2 years | + |  | Hyper- and hypopigmentation on right chest, upper back, and axillary regions. Sharp midline demarcation. | NA |
| Jain et al. 2012 | 237 | M | 2 years/At birth | + |  | Hyperpigmented macules on trunk and extremities. | N |
| Jenkins et al. 1993 | 238 | M | 8 years/- | + |  | Hypopigmentation on back and extremities. | NA |
| Kalter et al. 1988 | 239 | F | 1 year/At birth | + |  | Hyperpigmentated macules on the entire body, especially on flanks. | NA |
|  | 240 | M | 6 months/2-3 weeks after birth | + |  | Café-au-lait spots on most of the body. Midline demarcation. | N |
| Kang et al. 1996 | 241 | M | 45 years/At birth |  |  | Hypopigmented macules on lower back and forehead. | NA |
| Kanwar et al. 1993 | 242 | M | 23 years/At birth | + |  | Hyperpigmentation on the entire body and right half of the face. | N |
| Kayser et al. 2000 | 243 | M | 6 years/At birth | + |  | Hyper- and hypopigmentation on the entire body. | NA |
| Keng et al. 2006 | 244 | M | 6 years/- | + |  | Hypopigmentation on back, buttock, and left flank. | N |
| Khandpur et al. 2006 | 245 | M | 5 years/At birth | + |  | Hypopigmentation on trunk and extremities. | NA |
| Kiritsi et al. 2015 | 246 | M | 12 years/1 year | + |  | Hypopigmentation on trunk and upper extremities. | N |
| Koifmann et al. 1993 | 247 | F | 7 years/- |  |  | Hypopigmented regions on extremities. | N |
| Kosaki et al. 2008 | 248 | M | 44 years/- | + |  | Hyperpigmentation on upper extremities and midfacial area. | NA |
| Kroisel et al. 2000 | 249 | M | 5 years/At birth | + |  | Hyperpigmentation on back. | NA |
| Kubota et al. 1992 | 250 | F | 15 years/1 year | + |  | Hyperpigmentation on trunk and flanks. | N |
| Kuwahara et al. 2001 | 251 | F | 2 months/At birth | + |  | Hypopigmentation on trunk and extremities. | N |
| Lal et al. 2015 | 252 | M | 3 years/- | + |  | Hyperpigmentation on trunk and extremities. | N |
| Larralde et al. 2005 | 253 | F | 11 years/- |  |  | Hyper- and hypopigmentation on neck, trunk and legs. | N |
| Leonard et al. 2002 | 254 | F | 17 years/At birth | + |  | Hypopigmentation on trunk and extremities, predominance on the right. | NA |
| Lipsker et al. 2008 | 255 | M | 6 years/- | + |  | Hypo-/hyperpigmentation trunk and lower extremities. Sharp midline demarcation. | NA |
| Llamas-Velasco et al. 2010 | 256 | F | 35 years/5 years | + |  | Hyperpigmentation on trunk and extremities. | N |
| Lu et al. 2007 | 257 | F | 17 years/2 years | + |  | Hyperpigmentation on abdomen and back. | N |
| Lungarotti et al. 1991 | 258 | F | 3 years/- |  | Patchy aspect | Hypopigmentation on back and left side of abdomen. Hyperpigmentation on right side of abdomen. Patchy aspect on lower extremities. | N |
| Magenis et al. 1999 | 259 | M | 18 years/19 months | + |  | Hypopigmentation on back and lower extremities. | N |
| Maruani et al. 2012 | 260 | M | 3 months/10 days | + |  | Hyperpigmentation on trunk and extremities. | N |
| Mégarbané et al. 2002 | 261 | F | 24 years/4 months | + |  | Hyperpigmentation on trunk and posterior parts of the upper and lower extremities. | N |
| Mendiratta et al. 2001 | 262 | F | 15 years/Soon after birth | + |  | Hyperpigmentation on trunk and extremities. | N |
| Metta et al. 2011 | 263 | F | 12 years/6 months | + |  | Hyperpigmentation trunk and extremities. | Y (mother, grandmother) |
|  | 264 | F | 45 years/Early life | + |  | Hyperpigmentation on right buttocks, right upper extremity, and flexor side of the lower extremity. | Y (mother, daughter) |
|  | 265 | F | 65 years/Early life | + |  | Hyperpigmentation on buttocks and right thigh. | Y (daughter, granddaughter) |
| Meyer et al. 2004 | 266 | M | 32 years/First months of life | + |  | Hyperpigmentation on left shoulder and upper extremity. | NA |
| Morava et al. 2003 | 267 | F | 2.5 years/- |  |  | Café-au-lait spots and hyperpigmented region on trunk, right axillary area, abdomen, and thighs. Hypopigmentation in the hyperpigmented regions. | N |
| Morigaki et al. 2012 | 268 | F | 6 years/9 months | + |  | Hypopigmentation on neck and right upper extremity. | N |
| Muhammad et al. 2007 | 269 | M | 4 years/- | + |  | Hypopigmentation on trunk and extremities. | NA |
| Murano et al. 1991 | 270 | F | 19 years/- | + |  | Hypopigmentation on right side of the trunk and upper extremities. | NA |
|  | 271 | M | 19 years/- | + |  | Hypopigmentation on flexor surfaces of right upper and lower extremities. | NA |
| Myers et al. 2015 | 272 | F | 6 years/- |  | Phylloid pattern | Hypopigmented macules on trunk and extremities. | N |
| Naveen et al. 2014 | 273 | M | 33 years/2 years | + |  | Hyperpigmentation on trunk and thighs. | N |
| Nehal et al. 1996 | 274-311 | 22 F  16 M | Age at onset:  18/38 At birth  22/38 by the age of 2 years  14/38 after the age of 2 years | + 38/38 |  |  | NA |
|  | 312 | F | 13 years/At birth | + |  | Hypopigmentation on right neck and ear. | NA |
|  | 313 | M | 5.5 years/ Childhood | + |  | Hypopigmentation on right and left side of abdomen. | NA |
|  | 314 | M | 5 years/Infancy | + |  | Hypopigmentation on left side of the chest. | NA |
|  | 315 | F | 3.5 years/ Childhood | + |  | Hypopigmentation on right and left side of the trunk. | NA |
|  | 316 | M | 9.5 years/ Childhood | + |  | Hypopigmentation on right and left side of the trunk. | NA |
|  | 317 | F | 1.5 years/Infancy | + |  | Hypopigmentation on left side of the chest and upper extremity and lower extremities. | NA |
|  | 318 | F | 6 years/Childhood | + |  | Hypopigmentation on right side of the chest and left shoulder. | NA |
|  | 319 | M | 1.5 years/Infancy | + |  | Hypopigmentation on left side of the trunk. | NA |
|  | 320 | F | 11 years/Childhood | + |  | Hypopigmentation on right and left thigh. | NA |
|  | 321 | F | 2 years/Infancy | + |  | Hypopigmentation on trunk and extremities. | NA |
|  | 322 | F | 26 months/Infancy | + |  | Hyperpigmentation on left lower extremity. | NA |
|  | 323 | M | 3 months/Infancy | + |  | Diffuse hyperpigmentation. | NA |
|  | 324 | M | 2 years/Infancy | + |  | Hyperpigmentation on right side of the trunk and thigh. | NA |
|  | 325 | M | 11 years/Infancy | + |  | Hyperpigmentation on right side of the trunk. | NA |
|  | 326 | F | 4 years/Childhood | + |  | Diffuse hypo- and hyperpigmentation. | NA |
|  | 327 | F | 10 years/Infancy | + |  | Hypo- and hyperpigmentation on right and left side of the head and lower extremities. | NA |
| Nicita et al. 2012 | 328 | F | 10 years/Few months after birth |  |  | Hyperpigmented macules on spine and right lower extremity. Hypopigmented macules on left lower extremity. | NA |
| Niessen et al. 2005 | 329 | F | 6 years/One month after birth | + |  | Hyper- and hypopigmentation on trunk and lower extremities. | NA |
| Nishimura et al. 1998 | 330 | F | -/- |  |  | Hypopigmentation (linear) on lower extremities. | NA |
| Ogunbiyi et al. 1998 | 331 | F | 18 years/At birth | + |  | Hypopigmentation on trunk, upper extremities and thighs. | NA |
| Ohashi et al. 1992 | 332 | M | 6 months/- | + |  | Hyperpigmentation on lower abdomen. Hypopigmentation on thighs. | NA |
|  | 333 | F | 21 years/ - | + |  | Hyperpigmentation on right side of the body. Hypopigmentation on left chest and left upper extremity. | NA |
|  | 334 | F | 3 years/ - | + |  | Hypopigmentation on the back. | NA |
|  | 335 | F | 3 years/- | + |  | Hyperpigmentation on the entire body. | NA |
|  | 336 | M | 5 years/- | + |  | Hyperpigmentation on buttocks and flexor sides of the lower extremities. | NA |
|  | 337 | F | 20 months/- | + |  | Hypopigmentation on trunk and extremities. | NA |
|  | 338 | F | 8 years/ - | + |  | Hypopigmentation on trunk and extremities. | NA |
| Oiso et al. 2009 | 339 | F | 12 years/ - | + |  | Hyperpigmentation on right sole and palm. Hypopigmentation on right side of the trunk and extremities. | N |
| Oiso et al. 2010 | 340 | M | 29 years/- |  | Phylloid pattern | Hyperpigmentation on trunk and extremities. | N |
| Oiso et al. 2014 | 341 | M | 10 years/- | + |  | Hypopigmentation on neck and upper chest. | N |
| Ong et al. 1985 | 342 | F | 22 years/19 years |  |  | Hypopigmentation on trunk and extremities. | N |
| Ousager et al. 2006 | 343 | M | 48 years/20 years |  |  | Hyperpigmentation on left side of the trunk. Sharp midline demarcation. | NA |
| Ousager et al. 2012 | 344 | F | 40 years/At birth | + |  | Hyperpigmentation on left side of the trunk, extremities, neck, face, scalp, and external genital area. | NA |
| Palungwachira et al. 2006 | 345 | M | 5 years/Within the first week | + |  | Hypopigmentation on trunk and extremities. | N |
| Pascual-Castroviejo et al. 1998 | 346-421 | 41 F  35 M | Birth–10 years/  49/76 At birth  27/76 First years of life | + 76/76 |  | 76/76 hypopigmentation anywhere on the body. | Y (1/76 mother, 1/76 grand-mother)  N (74/76) |
| Patil et al. 2012 | 422 | F | 1 month/At birth | + |  |  | NA |
| Pellegrino et al. 1995 | 423 | M | 7 years/1 year | + |  | Hyper- and hypopigmentation on trunk and extremities, predominance on the right. | N |
| Petit et al. 2012 | 424 | F | 4.5 years/5.5 months | + |  | Hypopigmentation on trunk and flexor sides of the upper extremities. | N |
| Pillay et al. 1998 | 425 | F | 8 months/At birth | + |  | Hypopigmentation on face, trunk, and extremities. | NA |
| Pinheiro et al. 2007 | 426 | F | 5 years/Infancy | + |  | Diffuse hyperpigmentation. | NA |
|  | 427 | F | 2 years/At birth | + |  | Hyperpigmentation on left lower extremity. | NA |
|  | 428 | M | 3 years/At birth | + |  | Hyperpigmentation on right lower extremity. | NA |
|  | 429 | M | 1.5 years/At birth | + |  | Hyperpigmentation on lower extremities and sacral area. | NA |
|  | 430 | M | 3.5 years/At birth | + |  | Hyperpigmentation on left lower extremity. Hypopigmentation on left side of the trunk and left upper extremity. | NA |
|  | 431 | F | 3.5 years/At birth | + |  | Hyperpigmentation on left upper and lower extremities. Hypopigmentation on left upper extremity. | NA |
|  | 432 | M | 12 years/At birth | + |  | Hyperpigmentation on left gluteal region and thigh. | NA |
|  | 433 | F | 2.5 years/At birth | + |  | Hyperpigmentation on trunk, thighs and perineum. | NA |
|  | 434 | M | 9 months/At birth | + |  | Hyperpigmentation on lower extremities and gluteal region. | NA |
|  | 435 | F | 3 years/At birth | + |  | Hyperpigmentation on left lower extremity. | NA |
|  | 436 | M | 5 years/At birth | + |  | Hyperpigmentation on left side of the trunk. | NA |
|  | 437 | M | 3 years/At birth | + |  | Hyperpigmentation on right lower extremity. | NA |
|  | 438 | M | 5 years/At birth | + |  | Hyperpigmentation on left side of the trunk. | NA |
|  | 439 | M | 6 years/At birth | + |  | Hyperpigmentation on right side of the face. | NA |
|  | 440 | M | 1.5 years/At birth | + |  | Hyperpigmentation on left side of the trunk and right groin. | NA |
|  | 441 | F | 11 years/Childhood | + |  | Diffuse hyperpigmentation. | NA |
|  | 442 | F | 5 years/At birth | + |  | Diffuse hyperpigmentation. | NA |
|  | 443 | F | 2.5 years/At birth | + |  | Diffuse hypopigmentation. | NA |
|  | 444 | M | 12 years/At birth | + |  | Diffuse hypopigmentation. | NA |
|  | 445 | F | 3 years/At birth | + |  | Diffuse hypopigmentation. | NA |
|  | 446 | M | 5 years/3 years | + |  | Hypopigmentation on left side of the trunk and left upper extremity. | NA |
|  | 447 | F | 3 years/Infancy | + |  | Diffuse hyperpigmentation. | NA |
|  | 448 | F | 4 years/At birth | + |  | Hyperpigmentation on right groin. | NA |
|  | 449 | M | 1.5 years/At birth | + |  | Hyperpigmentation on right side of the neck. | NA |
|  | 450 | F | 2.5 years/At birth | + |  | Diffuse hyperpigmentation. | NA |
|  | 451 | M | 10 months/At birth | + |  | Hyperpigmentation on left thigh. | NA |
| Pini et al. 1995 | 452 | M | 13 years/3 years |  |  | Hypopigmentation on trunk and left lower extremity. Midline demarcation. | N |
| Pinto de Gouveia et al. 2016 | 453 | M | 9 years/At birth | + |  | Hyper- and hypopigmentation on trunk. | N |
| Ponti et al. 2014 | 454 | F | 6 months/3 months | + |  | Hypopigmentation on the trunk and right lower extremity. Midline demarcation. | Y (father) |
|  | 455 | M | -/At birth | + |  | Hypopigmentation on upper chest. | Y (daughter) |
| Portnoï et al. 1999 | 456 | M | 22 years/10-12 years | + |  | Hyperpigmentation of the entire body, except mucous membranes, soles and palms. | N |
| Pulimood et al. 1997 | 457 | M | 3 years/At birth | + |  | Hypopigmentation on trunk and extremities. | N |
|  | 458 | M | 4 years/At birth | + |  | Hypopigmentation on trunk and extremities | N |
| Quecedo et al. 1997 | 459 | M | 2 years/Several weeks after birth | + |  | Hyperpigmentation on trunk and extremities. | N |
| Quigg et al. 2006 | 460 | M | 15 years/- | + |  | Hypopigmentation on lumbar region and right upper and lower extremities. | NA |
| Ravel et al. 2001 | 461 | M | At birth/At birth |  |  | Diffuse hyper- and hypopigmentation on thighs. | NA |
| Resende et al. 2013 | 462 | F | 24 years/23 years |  |  | Hyperpigmented macules on right side of the upper back and shoulder. | N |
| Ribeiro Noce et al. 2001 | 463 | F | 3 years/At birth |  | Phylloid pattern | Hyperpigmentation on trunk. Hypopigmentation on the entire body. | N |
| Ritter et al. 1990 | 464 | M | 6 years/At birth | + |  | Hypopigmentation on trunk and extremities. | N |
| Rittinger et al. 2008 | 465 | F | 14 years/10 years |  |  | Hyperpigmented regions, predominant on buttocks and distal extensor sides of the upper extremities. | N |
| Romano et al. 1999 | 466 | F | 17 years/Infancy | + |  | Hyperpigmentation on extremities, predominance on the right. | N |
| Rott et al. 1990 | 467 | M | 10 years/At birth | + |  | Hypopigmentation on the entire body. | NA |
| Ruggieri 2000 | 468 | M | 11 years/At birth |  | Sash-like pattern | Hyperpigmentation on trunk and right mandible. Hypopigmentation on right supraclavicular area. | NA |
|  | 469 | M | 6 years/3 months |  | Sash-line pattern | Hyper- and hypopigmentation on trunk, extremities and left side of the face. | NA |
| Ruggieri et al. 2003 | 470 | F | 11 years/First few months |  |  | Hyper- and hypopigmentation on trunk and upper extremities. | N |
| Ruggieri et al. 2009 | 471 | M | 10 years/- | + |  | Hypopigmentation on trunk, buttocks, and extremities. | Y (mother) |
| Ruiz-Maldonado et al. 1992 | 472 | F | 16 years 9 months/2 years | + |  | Hypopigmentation on trunk and extremities. | N |
|  | 473 | M | 11 years/At birth |  | Patchy pattern | Hypopigmentation on thorax and upper right extremity. | N |
|  | 474 | F | 2 years 8 months/2 months | + |  | Hypopigmentation on right side of the body. | N |
|  | 475 | F | 2 years 3 months/3 months | + |  | Hypopigmentation on trunk and extremities. | N |
|  | 476 | F | 2 years 1 month/At birth | + |  | Hypopigmentation on left side of the body. | N |
|  | 477 | M | 9 years 10 months/- |  |  | Hypopigmentation on right side of the body. | N |
|  | 478 | M | 4 years/2 months | + |  | Hypopigmentation on trunk and right upper extremity. | N |
|  | 479 | F | 1 year 2 months/At birth |  | Patchy pattern | Hypopigmentation on trunk and extremities. | N |
|  | 480 | F | 1 year 10 months/At birth |  |  | Hypopigmentation on trunk and extremities. | N |
|  | 481 | M | 9 months/3 months | + |  | Hypopigmentation on trunk and extremities. | N |
|  | 482 | F | 1 year/At birth |  |  | Hypopigmentation on trunk and extremities. | N |
|  | 483 | M | 5 years 2 months/At birth | + |  | Hypopigmentation on right side of the body. | N |
|  | 484 | F | 1 year 2 months/3 months | + |  | Hypopigmentation on trunk and extremities. | N |
|  | 485 | M | 11 months/5 months |  | Patchy pattern | Hypopigmentation on left side of the trunk. | N |
|  | 486 | M | 10 years 6 months/At birth | + |  | Hypopigmentation on trunk and extremities. | N |
|  | 487 | M | 1 year 5 months/At birth | + |  | Hypopigmentation on trunk and extremities. | N |
|  | 488 | F | 3 years 5 months/At birth | + |  | Hypopigmentation on upper extremities, neck, thorax, and mandible. | N |
|  | 489 | M | 9.5 years/At birth | + |  | Hypopigmentation on left shoulder and upper extremity. | N |
|  | 480 | M | 6 months/2 months | + |  | Hypopigmentation on left side of the body. | N |
|  | 491 | F | 2 years 11 months/8 months | + |  | Hypopigmentation on left side of the body and right lower extremity. | N |
|  | 492 | F | 3.5 years/1 month | + |  | Hypopigmentation on extremities. | N |
|  | 493 | F | 3 years 7 months/At birth | + |  | Hypopigmentation on right side of the neck and upper right extremity. | N |
|  | 494 | M | 5 months/1 month |  | Patchy pattern | Hypopigmentation on right side of the body. | N |
|  | 495 | M | 1 year 9 months/At birth |  | Patchy pattern | Hypopigmentation on left side of the body. | N |
|  | 496 | M | 4 years/8 months | + |  | Hypopigmentation on left side of the neck, thorax, and upper extremity. | N |
|  | 497 | F | 1 year 2 months/At birth | + |  | Hypopigmentation on trunk and extremities. | N |
|  | 498 | F | 6 years 7 months/At birth | + |  | Hypopigmentation on thorax and extremities. | N |
|  | 499 | F | 5 years 3 months/At birth |  | Patchy pattern | Hypopigmentation on right side of the body. | N |
|  | 500 | M | 2 years 1 month/15 days | + |  | Hypopigmentation on trunk and extremities. | N |
|  | 501 | M | 8 years 4 months/At birth | + |  | Hypopigmentation on right side of the body. | N |
|  | 502 | F | 7 years 4 months/At birth |  | Patchy pattern | Hypopigmentation on trunk and extremities. | N |
|  | 503 | M | 1 year 5 months/6 months | + |  | Hypopigmentation on trunk and extremities. | N |
|  | 504 | F | 6 years 2 months/2 months | + |  | Hypopigmentation on trunk and extremities. | N |
|  | 505 | F | 1 year 2 months/At birth |  | Patchy pattern | Hypopigmentation on left side of the body. | N |
|  | 506 | F | 3 years 8 months/At birth | + |  | Hypopigmentation on left side of the back and extremities. | N |
|  | 507 | F | 1 year 4 months/At birth |  | Patchy pattern | Hypopigmentation on trunk and right side of the body. | N |
|  | 508 | M | 4 years/6 months |  |  | Hypopigmentation on trunk and extremities. | N |
|  | 509 | M | 1 year/At birth | + |  | Hypopigmentation on trunk, neck, and extremities. | N |
|  | 510 | M | 3 years/- | + |  | Hypopigmentation on left thorax and upper extremity. | N |
|  | 511 | F | 5 months/3 months |  |  | Hypopigmentation on left side of the body. | N |
|  | 512 | M | 6 years/At birth | + |  | Hypopigmentation on trunk and extremities. | N |
| Sarma 2012 | 513-580 | 29 F  39 M | 13 months to 29 years/Mostly within few months after birth | + 68/68 |  | Hyper- and hypopigmentation anywhere on the body, predominant on periorbital areas. | NA |
| Saxena et al. 1989 | 581 | F | 3 months/2 weeks after birth | + |  | Hypopigmentation on the entire body. | NA |
| Schepis et al. 1996 | 582 | M | 12 years/At birth | + |  | Hyperpigmentation on the back, flexor sides of the upper extremities, and lower extremities, predominant on the left. | NA |
| Schepis et al. 1999 | 583 | M | 15 years/12 years | + |  | Hyperpigmentation on trunk, ankles, thighs, hips, predominant on the left. | NA |
| Schepis et al. 2001 | 584 | F | 6 years/At birth |  | Phylloid pattern | Hyper- and hypopigmentation on trunk. Hypopigmentation on lower extremities. | NA |
| Scott et al. 2008 | 585 | M | 4 months/- | + |  | NA | NA |
| Shah et al. 2012 | 586 | M | 2 years/- | + |  | Hyper- and hypopigmentation on groin and lower extremities. | NA |
| Sharma et al. 2008 | 587 | M | 25 years/Childhood |  |  | Hypopigmented macule on right cheek, right lower eyelid and right iris. | NA |
| Shimizu et al. 2013 | 588 | F | 9 years/3 months | + |  | Hypopigmentation on trunk and extremities. | N |
|  | 589 | M | 6 years/6 years | + |  | Hypopigmentation on the back and left upper extremity. | N |
| Sigurdardottir et al. 1999 | 590 | F | 15 months/- | + |  | Hyper- and hypopigmentation on forehead and chest. | N |
|  | 591 | M | 16 months/- |  | Patchy pattern | Hyperpigmentation on trunk, especially on abdomen. Café-au-lait spots on extremities. | N |
| Singh et al. 2014 | 592 | M | 28 years/- |  | Phylloid pattern | Hypopigmentation on left side of the trunk. | NA |
| Steijlen et al. 2000 | 593 | F | 2 years/- | + |  | NA | NA |
|  | 594 | F | 3 years/- |  | Phylloid pattern | Hypopigmentation on trunk. | NA |
|  | 595 | F | 1.5 years/- | + |  | NA | NA |
|  | 596 | M | 9 years/- | + |  | NA | NA |
|  | 597 | F | 4 years/- | + |  | NA | NA |
| Stoll et al. 2002 | 598 | M | 3.5 years/At birth |  | Checkerboard pattern | Hyperpigmentation on trunk. Sharp midline demarcation. | N |
| Strømme et al. 2005 | 599 | F | 16 years/5 years | + |  | NA | NA |
| Sybert et al. 1990 | 600 | F | -/6 years 10 months | + |  | NA | N |
|  | 601 | F | -/2 years 5 months | + |  | Hypopigmentation on right lower extremity and abdomen. | N |
|  | 602 | F | -/9 years | + |  | Hypopigmentation on trunk and extremities. | N |
|  | 603 | F | -/3.5 years | + |  | Hypopigmentation on body and lower extremities. | N |
|  | 604 | M | -/9 years |  |  | Hypopigmentation on trunk. | N |
|  | 605 | F | -/3 years | + |  | Hypopigmentation on trunk and extremities. | N |
|  | 606 | F | -/13 months |  |  | Hypopigmentation on trunk. | N |
|  | 607 | F | -/1 years 9 months |  |  | NA | N |
|  | 608 | F | -/At Birth | + |  | NA | N |
|  | 609 | M | -/18 years | + |  | Hyper- and hypopigmentation on trunk and lower extremities. | N |
|  | 610 | F | -/3 years | + |  | Hyper- and hypopigmentation on body and extremities. | N |
|  | 611 | M | -/11 years | + |  | Hyper- and hypopigmentation on the trunk. | N |
|  | 612 | M | -/15 years |  |  | Hyperpigmentation on proximal extremities. Hypopigmentation on distal lower extremities. | N |
| Taibjee et al. 2009 | 613 | (5F 5M) | 12 years/- | + |  | Hyperpigmentation on chest. | N |
|  | 614 |  | 14 years/- | + |  | NA | N |
|  | 615 |  | 3 years/- | + |  | Hypopigmentation on left hip and chest. | N |
|  | 616 |  | 5 years/- | + |  | Hypopigmentation on back. | N |
|  | 617 |  | 6 years/- | + |  | Hypopigmentation on right lower extremity. | N |
|  | 618 | F | 18 years/6 months | + |  | Hypopigmentation on left side of the neck, right extremities. | N |
|  | 619 |  | 12 years/- | + |  | Hyperpigmentation on lower extremities. | N |
|  | 620 |  | 18 years/- | + |  | Hyperpigmentation on left chest, upper extremity and thigh. | N |
|  | 621 |  | 6 years/- | + |  | Hypopigmentation on upper extremities. | N |
|  | 622 |  | 8 years/- | + |  | Hyperpigmentation on chest and shoulders. | N |
| Thapa et al. 2007 | 623 | M | 2 years/At birth | + |  | Hyper- and hypopigmentation on the entire body. | NA |
| Thomas et al. 1989 | 624 | F | 7 years/At birth | + |  | Hyperpigmentation on the entire body. | NA |
|  | 625 | F | 2 years/- | + |  | Hyperpigmentation on trunk and extremities. | NA |
|  | 626 | M | 11 months/- | + |  | Hypopigmentation on posterior surfaces of extremities. | NA |
|  | 627 | F | 8 years/During the first few years of life | + |  | Hypopigmentation on lower extremities, including soles of the feet. | NA |
|  | 628 | F | 4.5 years/- | + |  | Hyperpigmentation on lower thorax and abdomen, midline demarcation. Hypopigmentation on abdomen. | NA |
|  | 629 | F | 8 years/- | + |  | Hypopigmentation on trunk and extremities. | NA |
|  | 630 | F | 18 years/- | + |  | Hyperpigmentation on trunk and extremities. | NA |
|  | 631 | M | 6 years/Infancy | + |  | Hypopigmentation on flexor sides of the lower extremities. | NA |
| Toelle et al. 2006 | 632 | F | 10 years/Infancy |  | Checkerboard pattern | Hyperpigmentation on trunk, groin, and face. | N |
| Toll et al. 2007 | 633 | F | 22 years/- | + |  | Hypopigmentation streaks on the back. | NA |
| Trägårdh et al. 2014 | 634 | M | 2.5 years/2.5 years | + |  | Hypopigmentation on the back. | NA |
| Tsutsumi et al. 1991 | 635 | F | 6 years/At birth | + |  | Hyper- and hypopigmentation on trunk and extremities. | N |
| Tunca et al. 2000 | 636 | F | 1 day old/At birth | + |  | Hyperpigmentation on buttocks, groin, axillae, and proximal lateral thighs. | N |
| Turleau et al. 1986 | 637 | F | 1 year/- |  |  | Hypopigmentation on trunk and extremities. | NA |
| Verghese et al. 1999 | 638 | M | 4.5 years/10 months | + |  | Hyperpigmentation on trunk and extremities. | N |
| Vormittag et al. 1992 | 639 | F | 33 years/Since childhood | + |  | Hypopigmentation on trunk and extremities. | N |
|  | 640 | F | 4 years/After first year of life | + |  | Hypopigmentation of the skin, clearly following the lines of Blaschko. | Y (mother) |
| Weaver et al. 1991 | 641 | F | 10 years/- | + |  | Hypopigmentation on trunk and extremities. | N |
| Woods et al. 1994 | 642 | M | 3 years/Infancy | + |  | Hyperpigmented region from mid-thoracic back to left axilla. Hypopigmentation on the entire body. | NA |
|  | 643 | F | 14 years/- | + |  | Hyperpigmentation on spine, right shoulder and right upper extremity. Hypopigmentation on flexor sides of the lower extremities. | N |
|  | 644 | F | 13 years/- | + |  | Hypopigmentation on trunk and extremities. | N |
|  | 645 | F | 3 years/ - | + |  | Hyperpigmentation on flanks, lower abdomen | N |
|  | 646 | F | 19 years/ - | + |  | Hyperpigmentation on right upper extremity. | N |
| Wulfsberg et al. 1991 | 647 | F | 9 years/- | + |  | Hyperpigmentation on upper extremities, left groin and upper thighs. | Y (twin sister) |
|  | 648 | F | 9 years/- | + |  | Hyperpigmentation on left antecubital fossa and upper thigh. | Y (twin sister) |
| Yakinci et al. 2002 | 649 | M | 6 months/1 week | + |  | Hypopigmentation on trunk and extremities. | NA |
| Yim et al. 1996 | 650 | F | 45 months/2.5 months | + |  | Hyperpigmentation on the entire body, except on face, palms, soles, eyes, and mucous membranes. | N |
| Yuksek et al. 2007 | 651 | M | 20 years/6 months | + |  | Hyperpigmentation on trunk, neck, perioral area, genitalia, and upper extremities. | NA |
| Total |  | 349F  302M |  | 516 | 19 Phylloid  12 Patchy  3 Checkerboard  2 Sash-like |  | 25 Y  342 N  284 NA |
