# Supplementary material for: Pigmentary mosaicism: a review of original literature and recommendations for future handling
Source: Orphanet J Rare Dis. 2018 Mar 5;13:39. doi: 10.1186/s13023-018-0778-6 (PMC5839061; doi:10.1186/s13023-018-0778-6)
Supplement: Supplementary file 2 — Extracutaneous manifestations. (DOCX 170 kb) [file 13023_2018_778_MOESM2_ESM.docx]

Additional file 2: Extracutaneous manifestations

| **References** | **Case number** | **Extracutaneous manifestations**  **No Yes** | |  |
| --- | --- | --- | --- | --- |
| Afsar et al. 2007 | 1 | X |  | |
| Aguayo-Leiva et al. 2011 | 2 |  | Skeletal deformities. | |
| Akahoshi et al. 2004 | 3 |  | Skeletal deformities, seizures, dysmorphic facial features, psychomotor retardation, hypotonia, hernia, hearing loss. | |
| Akiyama et al. 1994 | 4 | X |  | |
|  | 5 | X |  | |
| Al Aboud et al. 2005 | 6 | X |  | |
|  | 7 |  | Genital ulcers. | |
| Alrobaee et al. 2004 | 8 |  | Developmental delay, seizures, dysmorphic facial features, psychomotor retardation, heart abnormalities. | |
| Alvarez et al. 1993 | 9 | X |  | |
| Baba et al. 2002 | 10 | X |  | |
| Baba et al. 2003 | 11 |  | Breast hypoplasia. | |
|  | 12 | X |  | |
| Ballmer-Weber et al. 1996 | 13 |  | Skeletal deformities, dysmorphic facial features, dental abnormalities. | |
| Bartholomew et al. 1987 | 14 |  | Developmental delay, dysmorphic facial features, psychomotor retardation, hypertonia, microcephaly, ocular abnormality, dental abnormalities. | |
| Baty et al. 2001 | 15 |  | Skeletal deformities, oral and auditory abnormalities, hypertrichosis on lower spine. | |
|  | 16 |  | Skeletal deformities, ocular, auditory, oral and dental abnormalities. | |
| Bocian et al. 1993 | 17 |  | Skeletal deformities, dysmorphic facial and cranial features, psychomotor retardation, microcephaly, hypotonia, short neck, simian creases. | |
| Boente et al. 2011 | 18 |  | Skeletal deformities, dysmorphic facial and cranial features, microcephaly. | |
|  | 19 |  | Skeletal deformities, dysmorphic facial features, microcephaly. | |
| Boon et al. 1996 | 20 |  | Skeletal deformities, dysmorphic facial and cranial features, ocular and oral abnormalities, sacral dimple. | |
| Brar et al. 2008 | 21 | X |  | |
| Brock et al. 2012 | 22 |  | Skeletal deformities, seizures, psychomotor retardation, hypotonia, macrocephaly, ventriculomegaly, polythelia. | |
| Bygum et al. 2011 | 23 |  | Skeletal deformity. | |
| Bygum et al. 2012 | 24 |  | Dysmorphic facial features, psychomotor retardation, hypotonia, hair abnormalities, hearing loss, polythelia. | |
| Capaldi et al. 2005 | 25 | X |  | |
|  | 26 |  | Pterygium colli, lymphedema. | |
| Cappanera et al. 2011 | 27 |  | Skeletal deformities, seizures, psychomotor retardation, hypotonia, hypermobility, dental abnormalities. | |
| Castori et al. 2012 | 28 |  | Skeletal deformities, psychomotor retardation, kidney hypoplasia. | |
| Cellini et al. 1998 | 29 |  | Skeletal deformities, obesity. | |
| Chitayat et al. 1990 | 30 |  | Skeletal deformities, dysmorphic facial and cranial features, psychomotor retardation, hypotonia, hypermobility, hernia. | |
| Cho et al. 2011 | 31 | X |  | |
| Cho et al. 2012 | 32-61 |  | 1 patient: skeletal deformities, neurologic abnormalities. | |
| Choi et al. 2005 | 62 | X |  | |
| Cohen et al. 2014 | 63 |  | Speech delay. | |
|  | 64 | X |  | |
|  | 65 | X |  | |
|  | 66 | X |  | |
|  | 67 | X |  | |
|  | 68 | X |  | |
|  | 69 | X |  | |
|  | 70 | X |  | |
|  | 71 | X |  | |
|  | 72 | X |  | |
|  | 73 |  | Bilateral vesico-ureteral reflux. | |
|  | 74 |  | Skeletal deformity. | |
|  | 75 | X |  | |
|  | 76 | X |  | |
|  | 77 | X |  | |
|  | 78 | X |  | |
|  | 79 | X |  | |
|  | 80 | X |  | |
|  | 81 | X |  | |
|  | 82 |  | Hip dysplasia. | |
|  | 83 | X |  | |
|  | 84 | X |  | |
|  | 85 | X |  | |
|  | 86 | X |  | |
|  | 87 | X |  | |
|  | 88 | X |  | |
|  | 89 | X |  | |
|  | 90 | X |  | |
|  | 91 | X |  | |
|  | 92 | X |  | |
|  | 93 | X |  | |
|  | 94 | X |  | |
|  | 95 | X |  | |
|  | 96 | X |  | |
|  | 97 |  | Bilateral vesico-ureteral reflux, ADHD. | |
|  | 98 | X |  | |
| Correa-Cerro et al. 1997 | 99 |  | Skeletal deformities, seizures, dysmorphic facial and cranial features, hypotonia, ocular, dental and nipple abnormalities. | |
| Delaporte et al. 1996 | 100 | X |  | |
| Desai et al. 1988 | 101 |  | Skeletal deformities, psychomotor retardation, ocular and nipple abnormalities. | |
| Devillers et al. 2011 | 102 |  | Seizures, macrocephaly. | |
| Dhar et al. 2009 | 103 |  | Developmental delay, dysmorphic facial features, gynecomastia, genital abnormalities. | |
| Di Lernia 2007 | 104 |  | Atrophic gastritis, dizziness, anorexia nervosa. | |
|  | 105 | X |  | |
|  | 106 | X |  | |
|  | 107 | X |  | |
|  | 108 |  | Psychomotor delay, autism. | |
|  | 109 | X |  | |
|  | 110 | X |  | |
|  | 111 | X |  | |
|  | 112 | X |  | |
|  | 113 | X |  | |
|  | 114 | X |  | |
|  | 115 | X |  | |
|  | 116 | X |  | |
|  | 117 | X |  | |
|  | 118 | X |  | |
|  | 119 | X |  | |
| Di Lernia 2015 | 120 |  | Developmental delay, small for gestational age, hypertension, kidney abnormalities. | |
| Donnai et al. 1988 | 121 |  | Developmental delay, skeletal deformities, dysmorphic facial features, truncal obesity. | |
|  | 122 |  | Skeletal deformities, dysmorphic facial features, ocular abnormalities, truncal obesity. | |
|  | 123 |  | Developmental delay, skeletal deformities, macrocephaly. | |
| Dúran-McKinster et al. 2002 | 124 |  | Developmental delay, skeletal deformities, macrocephaly, cerebral abnormalities, ocular abnormalities. | |
| Eid et al. 2013 | 125 |  | Developmental delay, skeletal deformities, dysmorphic facial features, cerebral abnormalities, heart abnormalities. | |
| El-Sawy et al. 2011 | 126 |  | Ocular abnormalities. | |
| Errichetti et al. 2016 | 127 | X |  | |
| Ertam et al. 2009 | 128 | X |  | |
| Faletra et al. 2012 | 129 |  | Skeletal deformities, dysmorphic facial features, psychomotor delay, hypoplastic stomach. | |
|  | 130 |  | Development delay, skeletal deformities, dysmorphic facial features, cerebral abnormalities, genital abnormalities. | |
| Fan et al. 1994 | 131 |  | Seizures, cerebral and heart abnormalities. | |
| Finkelstein et al. 1992 | 132 | X |  | |
| Fleury et al. 1986 | 133 |  | Developmental delay, skeletal deformities, seizures, dysmorphic facial features, hypotonia, cerebral abnormalities, genital abnormalities, ocular and dental abnormalities. | |
|  | 134 |  | Developmental delay, hypertonia, ocular abnormalities. | |
|  | 135 |  | Developmental delay, seizures, cerebral abnormalities. | |
|  | 136 |  | Kidney abnormalities, macroglossia, diastasis recti. | |
| Fogu et al. 2008 | 137 |  | Skeletal deformities, seizures, dysmorphic facial features, hypertonia. | |
| Fritz et al. 1998 | 138 |  | Developmental delay, seizures, dysmorphic facial features, hypotonia, lymphedema. | |
| Fujimoto et al. 1985 | 139 |  | Developmental delay, skeletal deformities, dysmorphic facial and cranial features, psychomotor retardation, heart abnormalities, dental abnormalities. | |
| Fujino et al. 1995 | 140 |  | Seizures, hypotonia. | |
| Garcia Muret et al. 2002 | 141 |  | Seizures/abnormal EEG, cerebral and cranial abnormalities, hemiparesis of left hand. | |
| George et al. 1992 | 142 | X |  | |
| Gerdes et al. 2006 | 143 |  | Developmental delay, skeletal deformities, seizures, dysmorphic facial features, macrocephaly, umbilical hernia, auditory and dental abnormalities. | |
| Gonzalez-del Angel et al. 2014 | 144 |  | Developmental delay, skeletal deformities, dysmorphic facial and cranial features, oral and auditory abnormalities, metabolic syndrome. | |
| González-Ensenat et al. 2009 | 145 |  | Developmental delay, skeletal deformities, hair abnormalities. | |
|  | 146 |  | Developmental delay, skeletal deformities, seizures, neurologic abnormalities, auditory and dental abnormalities. | |
| Grazia et al. 1993 | 147 |  | Developmental delay, skeletal deformities, facial asymmetry, hypotonia, cerebral and neurologic abnormalities, dental abnormalities | |
| Griebel et al. 1989 | 148 |  | Skeletal deformities, seizures/abnormal EEG, dysmorphic facial features, psychomotor retardation, hypotonia, cerebral and neurologic abnormalities, varicosis. | |
|  | 149 |  | Skeletal deformities, seizures/abnormal EEG, dysmorphic facial features, psychomotor retardation, hypotonia, neurologic abnormalities. | |
|  | 150 |  | Seizures/abnormal EEG, psychomotor retardation, hypotonia, cerebral and neurologic abnormalities, macrocephaly, hearing loss. | |
|  | 151 |  | Skeletal deformities, dysmorphic facial features, psychomotor retardation, hypotonia, microcephaly, ocular and auditory abnormalities. | |
| Gupta et al. 2007 | 152 | X |  | |
| Gutte 2014 | 153 | X |  | |
| Hansen et al. 2003 | 154 |  | Developmental delay, skeletal deformities, seizures, dysmorphic facial features, psychomotor retardation, neurologic abnormalities. | |
| Hansen et al. 2010 | 155 |  | Developmental delay, seizures, psychomotor delay. | |
| Happle et al. 1997 | 156 |  | Developmental delay, skeletal deformities, seizures, dysmorphic facial features, psychomotor retardation, ocular and heart abnormalities. | |
| Happle 2009 | 157 | X |  | |
|  | 158 | X |  | |
|  | 159 | X |  | |
|  | 160 | X |  | |
| Happle et al. 2012 | 161 |  | Dysmorphic facial and cranial features, psychomotor delay, cerebral and neurologic abnormalities, macrocephaly, auditory abnormalities, hearing loss. | |
|  | 162 |  | Developmental delay, abnormal EEG, dysmorphic facial features, auditory, ocular, and dental abnormalities, alopecia, umbilicated nipples. | |
|  | 163 |  | Dysplasia of the large vessels. | |
|  | 164 |  | Skeletal deformities, muscular hypertrophy, spina bifida. | |
| Hartmann et al. 2004 | 165 |  | Psychomotor retardation, heart abnormality. | |
| Hassab-El-Naby et al. 1996 | 166 |  | Developmental delay, skeletal deformities, abnormal EEG, hypertonia, cerebral and neurologic abnormalities. | |
| Hernandez-Martin et al. 2014 | 167-191 | X |  | |
| Hogeling et al. 2010 | 192-230 |  | 1/39 with segmental hypopigmentation: atrial septal defect.  1/39 with segmental hyperpigmentation: strabismus with retinal hypopigmentation.  1/39 with hyperpigmentation: bronchogenic cyst. | |
| Hong et al. 2008 | 231 |  | Skeletal deformities. | |
| Horn et al. 1997 | 232 |  | Developmental delay, skeletal deformities, dysmorphic facial features, psychomotor retardation, neurologic abnormalities, auditory, oral and dental abnormalities. | |
| Horn et al. 2002 | 233 |  | Developmental delay, skeletal deformities, facial asymmetry, short stature. | |
|  | 234 |  | Skeletal deformities, macrocephaly, facial asymmetry. | |
| Ishikawa et al. 1985 | 235 |  | Skeletal deformities, seizures, psychomotor retardation, heart abnormalities. | |
| Jagia et al. 2004 | 236 |  | Hypoplasia of right breast. | |
| Jain et al. 2012 | 237 |  | Hemiatrophy. | |
| Jenkins et al. 1993 | 238 |  | Developmental delay, seizures, dysmorphic facial and cranial features, cerebral abnormalities, oral and dental abnormalities. | |
| Kalter et al. 1988 | 239 | X |  | |
|  | 240 | X |  | |
| Kang et al. 1996 | 241 | X |  | |
| Kanwar et al. 1993 | 242 | X |  | |
| Kayser et al. 2000 | 243 |  | Development delay, psychomotor retardation, hearing loss. | |
| Keng et al. 2006 | 244 |  | Developmental delay, seizures. | |
| Khandpur et al. 2006 | 245 |  | Skeletal abnormalities, dysmorphic facial features, inguinal hernia. | |
| Kiritsi et al. 2015 | 246 |  | Developmental delay, behavioural disturbance. | |
| Koifmann et al. 1993 | 247 |  | Skeletal deformities, seizures, dysmorphic facial and cranial features, oral and dental abnormalities, widely spaced nipples, nail abnormalities. | |
| Kosaki et al. 2008 | 248 |  | Skeletal deformities, dental abnormalities, nail abnormalities. | |
| Kroisel et al. 2000 | 249 |  | Development delay, dysmorphic facial features, dental abnormalities, cerebral abnormalities, simian creases. | |
| Kubota et al. 1992 | 250 |  | Genital abnormalities. | |
| Kuwahara et al. 2001 | 251 |  | Cerebral abnormalities, heart abnormalities, optic nerve hypoplasia. | |
| Lal et al. 2015 | 252 |  | Developmental delay, hyperkinesia, asymmetric nipples. | |
| Larralde et al. 2005 | 253 |  | Skeletal deformities, seizures, oligodendroglioma, dental abnormalities. | |
| Leonard et al. 2002 | 254 |  | Developmental delay, skeletal deformities, dysmorphic facial features, omphalocele, heart and bladder abnormalities, duodenal obstruction, genital abnormalities, auditory, ocular, oral and dental abnormalities. | |
| Lipsker et al. 2008 | 255 | X |  | |
| Llamas-Velasco et al. 2010 | 256 |  | Skeletal deformities, hypermobility, amblyopia, hearing loss. | |
| Lu et al. 2007 | 257 | X |  | |
| Lungarotti et al. 1991 | 258 |  | Developmental delay, abnormal walking. | |
| Magenis et al. 1999 | 259 |  | Development delay, skeletal deformities, abnormal EEG, dysmorphic facial and cranial features, hypotonia, acidosis, cerebral abnormalities, heart abnormalities, dental abnormalities, short stature, hearing loss. | |
| Maruani et al. 2012 | 260 | X |  | |
| Mégarbané et al. 2002 | 261 |  | Dysmorphic facial and cranial features, cerebral and neurologic abnormalities, hepato-spleno megaly, ocular abnormalities, hearing loss. | |
| Mendiratta et al. 2001 | 262 | X |  | |
| Metta et al. 2011 | 263 | X |  | |
|  | 264 | X |  | |
|  | 265 | X |  | |
| Meyer et al. 2004 | 266 |  | Ocular abnormality. | |
| Morava et al. 2003 | 267 |  | Skeletal deformities, dysmorphic facial features, truncal obesity, hypopigmented hair. | |
| Morigaki et al. 2012 | 268 |  | Developmental delay, seizures, hypotonia, ocular abnormalities. | |
| Muhammad et al. 2007 | 269 |  | Developmental delay, seizures, tuberous sclerosis complex. | |
| Murano et al. 1991 | 270 |  | Developmental delay, skeletal deformities, dysmorphic facial features, joint contractures of lower extremities, oral abnormality, irregular menstrual cycle. | |
|  | 271 |  | Developmental delay, skeletal deformities, dysmorphic facial features, oral abnormalities, heart abnormalities, left knee joint contracture, displacement of anus. | |
| Myers et al. 2015 | 272 |  | Skeletal deformities, dysmorphic facial features, dental and ocular abnormality, premature adrenarche and pubic hair. | |
| Naveen et al. 2014 | 273 |  | Rheumatic heart disease. | |
| Nehal et al. 1996 | 274-311 | X |  | |
|  | 312 |  | Epilepsy. | |
|  | 313 |  | Down syndrome. | |
|  | 314 |  | Developmental delay, behavioural disturbances. | |
|  | 315 |  | Mosaic Turner syndrome. | |
|  | 316 |  | Skeletal deformities. | |
|  | 317 |  | Seizures, dental abnormalities. | |
|  | 318 |  | Developmental delay, migraines. | |
|  | 319 |  | Developmental delay, microcephaly, congenital syphilis, behavioural disturbances. | |
|  | 320 |  | Developmental delay, seizures, mental retardation, autism. | |
|  | 321 |  | Developmental delay, skeletal deformities, microcephaly, patent ductus arteriosus, hearing loss. | |
|  | 322 |  | Developmental delay, seizures, dysmorphic facial features, ventricular septal defect. | |
|  | 323 |  | Seizures, hydrocephalus, hypotonia, hemiparesis. | |
|  | 324 |  | Skeletal deformities, dysmorphic facial features, psychomotor delay, increased height and weight. | |
|  | 325 |  | Hydrocephalus and hypospadias. | |
|  | 326 |  | Developmental delay. | |
|  | 327 |  | Developmental delay, skeletal deformities, seizures. | |
| Nicita et al. 2012 | 328 |  | Skeletal deformities, seizures, dysmorphic facial features, neurological and behavioural disturbances. | |
| Niessen et al. 2003 | 329 |  | Developmental delay, growth retardation, mental retardation, buphthalmos of left eye. | |
| Nishimura et al. 1998 | 330 |  | Skeletal deformities, dysmorphic facial and cranial features, short stature, heart abnormalities, coccygeal skin folds. | |
| Ogunbiyi et al. 1998 | 331 |  | Linear verrocous lesions. | |
| Ohashi et al. 1992 | 332 |  | Developmental delay, seizures, hypotonia. | |
|  | 333 |  | Developmental delay, skeletal deformities, seizures, mental retardation, strabismus. | |
|  | 334 |  | Developmental delay, skeletal deformities, seizures, dysmorphic facial features, sparse hair. | |
|  | 335 |  | Developmental delay, mental retardation, auditory and dental abnormality. | |
|  | 336 |  | Developmental delay, seizures, hemiparesis, mental retardation, head asymmetry, strabismus. | |
|  | 337 |  | Skeletal deformities, seizures, dysmorphic facial features, heart abnormalities. | |
|  | 338 |  | Developmental delay, skeletal deformities, mental retardation, ocular, auditory and oral abnormalities, polythelia. | |
| Oiso et al. 2009 | 339 |  | Atrial septal defect. | |
| Oiso et al. 2010 | 340 |  | Developmental delay, mental retardation. | |
| Oiso et al. 2014 | 341 |  | Hearing loss. | |
| Ong et al. 1985 | 342 |  | Diabetes mellitus, goitre, behavioural disturbances, microaneurysm in retina, hair abnormalities. | |
| Ousager et al. 2006 | 343 |  | Skeletal deformities, dysmorphic facial and cranial features, inguinal hernia, auditory abnormalities, genital abnormalities, scaling on hands, alopecia. | |
| Ousager et al. 2012 | 344 |  | Epilepsy. | |
| Palungwachira et al. 2006 | 345 | X |  | |
| Pascual-Castroviejo et al. 1998 | 346-421 |  | 43/76 Developmental delay, 11/76 skeletal deformities, 37/76 seizures, 12/76 dysmorphic facial features, 14/76 hypotonia, 12/76 facial, trunk or member asymmetry, 12/76 macrocephaly, 6/76 microcephaly, 4/76 genital abnormalities, 4/76 dental abnormalities, 3/76 heart abnormalities, 3/76 ocular abnormalities, 3/76 death, 2/76 hypotrophy, 2/76 hair abnormalities, 1/76 gynecomastia, 1/76 asymmetrical breast, 18/76 other minor abnormalities. | |
| Patil et al. 2012 | 422 |  | Dysmorphic facial features, heart abnormalities, simian creases. | |
| Pellegrino et al. 1995 | 423 |  | Developmental delay, dysmorphic facial features, mental retardation, ADHD, stubby hands, simian crease. | |
| Petit et al. 2012 | 424 |  | Developmental delay, skeletal deformities, growth retardation, auditory abnormalities, gastroeosophageal reflux, feeding difficulties. | |
| Pillay et al. 1998 | 425 |  | Skeletal deformities, dysmorphic facial and cranial features, microcephaly, cerebral abnormalities, inguinal and umbilical herniae, oral abnormalities, polythelia. | |
| Pinheiro et al. 2007 | 426 |  | Developmental delay, seizures. | |
|  | 427 |  | Developmental delay, seizures, cerebral atrophy, spastic quadriparesis. | |
|  | 428 |  | Down syndrome. | |
|  | 429 |  | Developmental delay, paresis. | |
|  | 430 |  | Developmental delay, paresis, cerebellar abnormalities. | |
|  | 431 |  | Developmental delay, seizures, spastic paresis. | |
|  | 432 |  | Developmental delay, seizures. | |
|  | 433 |  | Developmental delay, seizures, hypertonia, paresis. | |
|  | 434 |  | Down syndrome. | |
|  | 435 |  | Developmental delay, seizures, paresis. | |
|  | 436 |  | Seizures, learning difficulty. | |
|  | 437 |  | Developmental delay, hypertonia, cerebral atrophy. | |
|  | 438 |  | Developmental delay, hyperkinesia, cerebral abnormalities, autism. | |
|  | 439 |  | Developmental delay, seizures, cerebral atrophy. | |
|  | 440 |  | Seizures, hyperkinesia. | |
|  | 441 |  | Developmental delay. | |
|  | 442 |  | Developmental delay, seizures. | |
|  | 443 |  | Seizures. | |
|  | 444 |  | Developmental delay, cerebral abnormalities. | |
|  | 445 |  | Developmental delay, seizures, cerebral abnormalities. | |
|  | 446 |  | Developmental delay, hyperactivity. | |
|  | 447 |  | Developmental delay, hyperactivity. | |
|  | 448 |  | Developmental delay, seizures, hyperactivity, ataxia. | |
|  | 449 |  | Developmental delay, quadriparesis. | |
|  | 450 |  | Developmental delay, hemiplegia, cerebral abnormalities. | |
|  | 451 |  | Developmental delay, hypertonia. | |
| Pinto de Gouveia et al. 2016 | 452 |  | Developmental delay, skeletal deformities, cranial deformities, hypertrichosis, dysorthography. | |
| Pini et al. 1995 | 453 |  | Developmental delay, skeletal deformities, dysmorphic facial features, macrocephaly, neurologic abnormalities. | |
| Ponti et al. 2014 | 454 | X |  | |
|  | 455 | X |  | |
| Portnoï et al. 1999 | 456 | X |  | |
| Pulimood et al. 1997 | 457 |  | Developmental delay, seizures, mental retardation, cerebral and neurologic abnormalities, ocular abnormalities, faecal and urinary incontinence. | |
|  | 458 |  | Developmental delay, dysmorphic facial features, cerebral abnormalities, ocular abnormalities, hypospadias, ankyloglossia. | |
| Quecedo et al. 1997 | 459 | X |  | |
| Quigg et al. 2006 | 460 |  | Seizures. | |
| Ravel et al. 2001 | 461 |  | Skeletal deformities, dysmorphic facial features, hypotonia, cryptorchidism. | |
| Resende et al. 2013 | 462 | X |  | |
| Ribeiro Noce et al. 2001 | 463 |  | Developmental delay, skeletal deformities, seizures, dysmorphic facial features, mental retardation, microcephaly, auditory and oral abnormalities. | |
| Ritter et al. 1990 | 464 |  | Developmental delay, dysmorphic facial and cranial features, hypotonia, mental retardation. | |
| Rittinger et al. 2008 | 465 |  | Developmental delay, skeletal deformities, seizures, dysmorphic facial features, psychomotor retardation, truncal obesity, simian creases. | |
| Romano et al. 1999 | 466 |  | Development delay, skeletal deformities, dysmorphic facial features, mental retardation, short stature, hypotrophic leg muscles, heart and ocular abnormalities, hearing loss, anteriorly placed anus. | |
| Rott et al. 1990 | 467 |  | Seizures, dysmorphic facial features, cerebral abnormalities. | |
| Ruggieri 2000 | 468 | X |  | |
|  | 469 |  | Skeletal deformities, dysmorphic facial features, psychomotor retardation. | |
| Ruggieri et al. 2003 | 470 |  | Developmental delay, skeletal deformities, seizures, dysmorphic facial and cranial features, psychomotor retardation, hypotonia, neurologic abnormalities, dental abnormalities, absent language, sphincter incontinence. | |
| Ruggieri et al. 2009 | 471 |  | Developmental delay, skeletal deformities, seizures, dysmorphic facial and cranial features, cerebral abnormalities, hypotonia, hyperactivity, cataract. | |
| Ruiz-Maldonado et al. 1992 | 472 |  | Skeletal deformities, microcephaly, dental abnormality. | |
|  | 473 |  | Developmental delay, seizures, mental retardation, microcephaly. | |
|  | 474 |  | Developmental delay, skeletal deformities, abnormal EEG, mental retardation, hydrocephalus, obesity, dacryostenosis. | |
|  | 475 |  | Developmental delay, seizures, psychomotor retardation. | |
|  | 476 |  | Developmental delay, seizures, psychomotor retardation, hydrocephalus, amaurosis. | |
|  | 477 |  | Developmental delay, skeletal deformities, abnormal EEG, psychomotor retardation, short stature, hypothyroidism, swallowing alterations. | |
|  | 478 |  | Developmental delay, seizures, psychomotor retardation, hyperkinesia, ataxia, scleral melanosis. | |
|  | 479 |  | Developmental delay, seizures, mental retardation, microcephaly, hypotonia. | |
|  | 480 |  | Developmental delay, skeletal deformities, dysmorphic facial features, psychomotor retardation, microcephaly, heart abnormalities. | |
|  | 481 |  | Developmental delay, psychomotor retardation, fibroma, inguinal hernias. | |
|  | 482 |  | Developmental delay, skeletal deformities, psychomotor retardation, microcephaly. | |
|  | 483 |  | Developmental delay, psychomotor retardation. | |
|  | 484 |  | Developmental delay, skeletal deformities, mental retardation, muscular hypotonia. | |
|  | 485 |  | Developmental delay, skeletal deformities, psychomotor retardation. | |
|  | 486 |  | Skeletal deformities, dysmorphic facial and cranial features, hypotonia, heart abnormalities, hypospadias, nystagmus, simian creases. | |
|  | 487 |  | Developmental delay, skeletal deformities, psychomotor retardation, microcephaly. | |
|  | 488 |  | Developmental delay, seizures, psychomotor retardation, ataxia. | |
|  | 489 |  | Seizures/abnormal EEG, low hairline. | |
|  | 480 |  | Skeletal deformities. | |
|  | 491 |  | Skeletal deformities, abnormal EEG, heart abnormality. | |
|  | 492 |  | Skeletal deformities, psychomotor retardation, ocular abnormalities, aplasia cutis of scalp, symblepharon. | |
|  | 493 |  | Developmental delay, seizures, psychomotor retardation. | |
|  | 494 |  | Developmental delay, abnormal EEG, psychomotor retardation. | |
|  | 495 |  | Developmental delay, seizures/abnormal EEG, mental retardation. | |
|  | 496 |  | Skeletal deformities, psychomotor retardation, short stature, hearing loss. | |
|  | 497 |  | Skeletal deformities, ureteral duplication. | |
|  | 498 |  | Developmental delay, skeletal deformities, abnormal EEG, psychomotor retardation, dental abnormalities. | |
|  | 499 |  | Skeletal deformities, seizures/abnormal EEG, hyperkinesia, atopic dermatitis. | |
|  | 500 |  | Developmental delay, skeletal deformities, mental retardation, hypotonia, hypertrophy, microcephaly, renal tubular acidosis, short stature. | |
|  | 501 |  | Seizures/abnormal EEG. | |
|  | 502 |  | Skeletal deformities, motor retardation, myelomeningocele, paraplegia, sphincter incontinence, cataract. | |
|  | 503 |  | Developmental delay, skeletal deformities, seizures/abnormal EEG, dysmorphic facial and cranial features, psychomotor retardation, microcephaly, hypotonia. | |
|  | 504 |  | Skeletal deformities, abnormal EEG, dysmorphic facial and cranial features, hyperkinesia, short stature. | |
|  | 505 |  | Developmental delay, seizures/abnormal EEG, dysmorphic facial and cranial features, psychomotor retardation, cerebral abnormality, hypotonia, nystagmus, gastroesophageal reflux. | |
|  | 506 |  | Seizures/abnormal EEG, psychomotor retardation, hypertonia, cerebral asymmetry, high palate. | |
|  | 507 |  | Developmental delay, skeletal deformities, seizures, dysmorphic facial features, psychomotor retardation, microcephaly, cerebral abnormalities, heart abnormality, dental abnormalities, strabismus, short neck, umbilical hernia, apnoea, simian creases. | |
|  | 508 |  | Developmental delay, skeletal deformities, psychomotor retardation, auditory abnormalities, low hairline. | |
|  | 509 |  | Skeletal deformities, dysmorphic facial features, psychomotor retardation. | |
|  | 510 |  | Developmental delay, mental retardation. | |
|  | 511 |  | Developmental delay, psychomotor retardation, apnoea, high palate. | |
|  | 512 |  | Skeletal deformities, auditory abnormality, high palate. | |
| Sarma 2012 | 513-580 | X |  | |
| Saxena et al. 1989 | 581 |  | Dental abnormalities. | |
| Schepis et al. 1996 | 582 |  | Developmental delay, skeletal deformities, dysmorphic facial features, mental retardation, cerebral abnormality, macrocephaly, ocular abnormalities, hearing loss. | |
| Schepis et al. 1999 | 583 |  | Developmental delay, skeletal deformities, mental retardation, hypotrophy, behavioural disturbances, polythelia. | |
| Schepis et al. 2001 | 584 |  | Developmental delay, dysmorphic facial and cranial features, mental retardation, short neck. | |
| Scott et al. 2008 | 585 |  | Seizures, dysmorphic facial features, cortical dysplasia. | |
| Shah et al. 2012 | 586 |  | Developmental delay, dysmorphic facial and cranial features, sparse hair, high arched palate with median furrow, hearing loss. | |
| Sharma et al. 2008 | 587 | X |  | |
| Shimizu et al. 2013 | 588 |  | Seizures, large left ventricle, dental abnormalities, strabismus, hearing loss, hypohidrosis. | |
|  | 589 |  | Hypohidrosis. | |
| Sigurdardottir et al. 1999 | 590 |  | Developmental delay, skeletal deformities, dysmorphic facial features, short stature, growth retardation, simian crease. | |
|  | 591 |  | Dysmorphic facial and cranial features, microcephaly, short stature, growth retardation, cherry-red spots. | |
| Singh et al. 2014 | 592 |  | Seizures, tremors, fatty liver. | |
| Steijlen et al. 2000 | 593 |  | Dysmorphic facial features, hypotonia, bilateral hip dysplasia. | |
|  | 594 |  | Dysmorphic facial features, dental abnormality, bilateral hip dysplasia. | |
|  | 595 |  | Dysmorphic facial features, hypoplasia of gluteal musculature. | |
|  | 596 |  | Mental retardation. | |
|  | 597 |  | Skeletal deformities, hypertonia, hypoplasia of depressor anguli oris, Raynaud’s phenomenon, polythelia. | |
| Stoll et al. 2002 | 598 |  | Dysmorphic facial and cranial features, hirsutism. | |
| Strømme et al. 2005 | 599 |  | Developmental delay, skeletal deformities, cerebral abnormalities. | |
| Sybert et al. 1990 | 600 |  | Developmental delay, skeletal deformities, seizures, dysmorphic facial features, heart abnormality. | |
|  | 601 |  | Developmental delay, skeletal deformities, dysmorphic facial features, psychomotor retardation, hypotonia. | |
|  | 602 |  | Developmental delay, seizures, macrocephaly. | |
|  | 603 |  | Developmental delay, seizures, macrocephaly. | |
|  | 604 |  | Skeletal deformities, hemiatrophy, hydrocephalus, retinal dysplasia. | |
|  | 605 |  | Developmental delay. | |
|  | 606 |  | Developmental delay, microcephaly, hypotonia, short stature. | |
|  | 607 |  | Developmental delay, dysmorphic facial features, microcephaly, heart abnormalities. | |
|  | 608 |  | Developmental delay, seizures, short stature, cutaneous syndactyly, lymphedema, aortic stenosis. | |
|  | 609 |  | Developmental delay, dysmorphic facial features. | |
|  | 610 |  | Developmental delay, dysmorphic facial features, microcephaly, short stature. | |
|  | 611 |  | Developmental delay, skeletal deformities, dysmorphic facial features, hypotonia, short stature, variegation of hair colour. | |
|  | 612 |  | Developmental delay, dysmorphic facial features, psychomotor retardation, short stature. | |
| Taibjee et al. 2009 | 613 |  | Developmental delay. | |
|  | 614 |  | Developmental delay, seizures. | |
|  | 615 |  | Developmental delay. | |
|  | 616 |  | Developmental delay, skeletal deformities, dysmorphic facial features, short stature, ear pit, polythelia. | |
|  | 617 |  | Developmental delay, hydrocephalus, cleft palate. | |
|  | 618 |  | Seizures. | |
|  | 619 |  | Developmental delay, autism. | |
|  | 620 |  | Developmental delay, dysmorphic facial features, choreoathetosis. | |
|  | 621 |  | Developmental delay, skeletal deformities, seizures, truncal obesity, ocular abnormalities. | |
|  | 622 |  | Developmental delay, skeletal deformities, dysmorphic facial features, dental abnormalities, sparse hair. | |
| Thapa et al. 2007 | 623 |  | Developmental delay, seizures, hypotonia, macrocephaly, dilated lateral cerebral ventricles, dental and hair abnormalities, hearing loss. | |
| Thomas et al. 1989 | 624 |  | Developmental delay, skeletal deformities, seizures, mental retardation, microcephaly, choanal stenosis. | |
|  | 625 |  | Psychomotor delay, microcephaly, short stature. | |
|  | 626 |  | Developmental delay, skeletal deformities, dysmorphic facial features, hypertonia, hyperreflexia, renal agenesis. | |
|  | 627 |  | Developmental delay, mental retardation, hypertonia, hyperreflexia, limited extension and flexion of joints. | |
|  | 628 |  | Developmental delay, hypotonia, hyporeflexia, carious teeth. | |
|  | 629 |  | Developmental delay, dysmorphic facial features, hyperactivity, horseshoe kidney. | |
|  | 630 |  | Developmental delay, seizures, mental retardation, meningomyelocele, obesity, short stature, death caused by seizure. | |
|  | 631 |  | Hypospadias. | |
| Toelle et al. 2006 | 632 |  | Cataract. | |
| Toll et al. 2007 | 633 |  | Hair and dental abnormalities. | |
| Trägårdh et al. 2014 | 634 |  | Developmental delay, skeletal deformities, mental retardation, hernia, glomerulonephritis, cryptorchidism, ocular abnormalities, hearing loss. | |
| Tsutsumi et al. 1991 | 635 |  | Labial talon cusp. | |
| Tunca et al. 2000 | 636 |  | Dysmorphic facial features, short neck, absent corpus callosum, growth retardation, heart abnormalities, hair abnormalities, contracted elbows. | |
| Turleau et al. 1986 | 637 |  | Skeletal deformities, seizures, hemiatropy, sacrococcygeal tumour, crural area of hypertrichosis. | |
| Verghese et al. 1999 | 638 |  | Developmental delay, skeletal deformities, dysmorphic facial and cranial features, psychomotor delay, cerebral abnormalities, hypotonia, oral, ocular and genital abnormalities, simian creases. | |
| Vormittag et al. 1992 | 639 |  | Skeletal deformities, seizures, ocular abnormalities. | |
|  | 640 | X |  | |
| Weaver et al. 1991 | 641 |  | Skeletal deformities, cerebral abnormalities, hypermobility, ocular and dental abnormalities, cleft palate, imperforate anus. | |
| Woods et al. 1994 | 642 |  | Developmental delay, skeletal deformities. | |
|  | 643 |  | Developmental delay, skeletal deformities, dysmorphic facial features, mental retardation, heart abnormalities, short stature, multiple exostoses. | |
|  | 644 |  | Developmental delay, skeletal deformities, seizures/abnormal EEG, dysmorphic facial and cranial features, mental retardation, cerebral abnormalities, precocious puberty, hyperthyroidism. | |
|  | 645 |  | Developmental delay, skeletal deformities, dysmorphic facial and cranial features, mental retardation, heart abnormalities, ocular abnormalities, hearing loss, simian creases. | |
|  | 646 |  | Skeletal deformities, dysmorphic facial features, mental retardation, heart abnormalities, short stature, dysplastic toenails, primary amenorrhoea, hearing loss. | |
| Wulfsberg et al. 1991 | 647 |  | Dysmorphic facial features, truncal obesity, cutaneous syndactyly, simian creases. | |
|  | 648 |  | Skeletal deformities, dysmorphic facial features, truncal obesity. | |
| Yakinci et al. 2002 | 649 |  | Skeletal deformities, seizures, dysmorphic facial features, psychomotor retardation, microcephaly, inguinal and umbilical hernia, cryptorchidism. | |
| Yim et al. 1996 | 650 |  | Developmental delay, dysmorphic facial features, hyperactivity of lower extremities, abnormal plantar reflexes. | |
| Yuksek et al. 2007 | 651 |  | Skeletal deformity. | |
