# Supplementary material for: Pigmentary mosaicism: a review of original literature and recommendations for future handling
Source: Orphanet J Rare Dis. 2018 Mar 5;13:39. doi: 10.1186/s13023-018-0778-6 (PMC5839061; doi:10.1186/s13023-018-0778-6)
Supplement: Supplementary file 3 — Diagnostic methods and karyotype. Karyotype, results of skin biopsies and cytogenetic analyses of skin fibroblasts and peripheral blood lymphocytes. (DOCX 114 kb) [file 13023_2018_778_MOESM3_ESM.docx]

Additional file 3: Diagnostic methods and karyotype

|  | **Case number** | **Sex** (F/M) | **Histopathology of skin biopsies**  TEM: Transmission electron microscopy | **Cytogenetic analysis of skin fibroblasts (F) and peripheral blood lymphocytes (L)**  Hyperpigmented skin (A), hypopigmented skin (B), normopigmented skin (N)  CGH: Comparative genomic hybridization | **Karyotype as noted in the papers** |
| --- | --- | --- | --- | --- | --- |
| Afsar et al. 2007 | 1 | F | Hyperpigmentation: Increased basal layer pigment but no naevomelanocytic proliferation. Epidermis with acanthosis and hyperkeratosis.  Hypopigmentation: Reduced basal layer pigment, normal thickness of epidermis.  Immunohistochemical staining with Melan-A and HMB-45 showed a slight increase in the number of melanocytes in hyperpigmented skin and a decrease in hypopigmented skin. |  |  |
| Aguayo-Leiva et al. 2011 | 2 | F |  |  |  |
| Akahoshi et al. 2004 | 3 | F |  | Analysis (L) showed mos 47,XY,+idic(15)(pter🡪q14::q14🡪pter)/46,XY.ish idic(15). | mos 47,XY,+idic(15)(pter🡪q14::q14🡪pter)/  46,XY.ish idic(15) |
| Akiyama et al. 1994 | 4 | F | Hyperpigmentation: Diffuse moderate hyperpigmentation in the basal layer and a slight increase in the number of melanocytes in the epidermis. Mild elongation of rete ridges was observed in some areas.  No pigmentary incontinence.  TEM: Increase in melanosomes in the cytoplasm of basal keratinocytes.  No abnormality seen in the melanocytes and no giant melanosomes were seen. | Analyses (F+L) showed normal karyotype. | 46,XX |
|  | 5 | F | Hyperpigmentation: Slight increase in melanocytes in the epidermis and irregular basal melanosis. No pigmentary incontinence or melanophages were seen.  TEM: Increase in fully pigmented melanosomes in the cytoplasm of the keratinocytes. The melanocytes were normal, and no giant melanosomes were seen. | Analyses (F+L) showed normal karyotype. | 46,XX |
| Al Aboud et al. 2005 | 6 | F | Hyperpigmentation: Basal cell hyperpigmentation with pigmentary incontinence. |  |  |
|  | 7 | F | Hyperpigmentation: Basal cell hyperpigmentation with pigmentary incontinence. |  |  |
| Alrobaee et al. 2004 | 8 | M | Hyperpigmentation: Increase in melanin content of the basal layer without pigmentary incontinence or melanophages in the dermis. |  |  |
| Alvarez et al. 1993 | 9 | M | Hyperpigmentation: Pigment in the basal epidermal layer. No pigmentary incontinence or melanophages in the dermis.  TEM: No macromelanosomes. | Analysis (L) showed normal karyotype. | 46,XY |
| Baba et al. 2002 | 10 | F | Hyperpigmentation: Increased melanocyte density with elongation of the rete ridges. No nests of melanocytes.  Hypopigmentation: No pathological features. |  |  |
| Baba et al. 2003 | 11 | F | Hyperpigmentation: Pigment incontinence and melanophages.  Hypopigmentation: Decreased number of epidermal melanocytes.  Intermediate pigmented skin: No pathological findings. |  |  |
|  | 12 | F |  |  |  |
| Ballmer-Weber et al. 1996 | 13 | F |  | Analysis (L) showed normal karyotype. | 46,XX |
| Bartholomew et al. 1987 | 14 | F |  | Analysis showed normal karyotype. | 46,XX |
| Baty et al. 2001 | 15 | F |  | Analysis (L) showed 15% trisomy 20 (4/26 cells) in chorionic villi after birth and 25% trisomy 20 (3/12 cells) in urinary cells.  Analysis (F) in 50 cells showed normal karyotype.  Analysis in 30 cord blood cells showed normal karyotype. | mos 47,XX,+20/46,XX |
|  | 16 | F |  | Analysis (L) showed 4/40 (10%) cells in cord, 5/10 (50%) cells in amniotic membrane, 10/10 (100%) cells in placenta, and 50/50 (100%) cells in urine to be trisomy 20. All 100 cells examined from cord blood were normal.  Analysis (F) showed trisomy 20 in 30%. | mos 47,XX,+20/46,XX |
| Bocian et al. 1993 | 17 | F |  | Analyses (F+L) showed a mosaic karyotype with three different structural abnormalities of chromosome 18 in fibroblasts of both tissues, F(A+B) and (L). The predominant karyotype was the deletion of 18p. | mos  46,XX,del(18)(p11.23🡪pter)/  46,XX,idic(18)(p11.23)/  46,XX,r(18)?(p11.23q23) |
| Boente et al. 2011 | 18 | F |  | Analysis (L) showed 46,XY,r(15)(p11.2q26). | 46,XY,r(15)(p11.2q26) |
|  | 19 | F |  | Analysis (L) showed 46,XX,r(15)(p12-q26.3). | 46,XX,r(15)(p12q26.3) |
| Boon et al. 1996 | 20 | F |  | Analysis (L) showed normal karyotype.  Analyses (F) of two cell lines showed that in 6/50 metaphase spreads, a marker chromosome was also present. The marker was the equivalent size of chromosome 16, but banding patterns more alike chromosome 10. | mos 46,XX/47,XX,+del(10)(q11.2q23.2) |
| Brar et al. 2008 | 21 | F | Diffuse basal cell hyperpigmentation with increase in the number of basal melanocytes. No incontinence of pigment, melanophages in dermis or giant melanosomes. |  |  |
| Brock et al. 2012 | 22 | M |  | Prenatal analysis of amniocytes showed normal karyotype.  Analysis (L) showed normal karyotype.  Analysis (F) showed mosaicism for a marker chromosome i(5)(p10).  Array-CGH on peripheral blood confirmed no abnormalities. | mos 47,XY,+i(5)(p10)/46,XY |
| Bygum et al. 2011 | 23 | F | Slightly papillomatous surface with non-specific laminated hyperkeratosis and acanthosis. | Analysis (F)(A+N): Snapshot assays identified FGFR3 hotspot mutation R248C in EN tissue, but not in normal skin.  Analysis (L) showed mosaicism for R248C FGFR3 mutation (mesoderm).  Analysis of buccal cells showed same mosaic (ectoderm). | mos of the R248C FGFR3 mutation |
| Bygum et al. 2012 | 24 | M |  | Analyses (F+L) showed normal karyotype.  Fibroblasts as well as keratinocytes from hyperpigmented skin showed normal karyotype. Confirmed by FISH and CGH performed twice.  Culturing of melanocytes was unsuccessful. | 46,XY |
| Capaldi et al. 2005 | 25 | F |  | Chromosomal analysis showed mosaic Turner syndrome, with 56% of cells showing 45,X and 44% of cells showing normal karyotype. | mos 45,X/46,XY |
|  | 26 | F |  | Chromosomal analysis showed mosaic Turner syndrome, with 95% of cells showing 45,X and 5% of cells showing normal karyotype. | mos 45,X/46,XY |
| Cappanera et al. 2011 | 27 | F |  | Analysis (L) showed that 34% carried ring chromosome 20.  FISH with telomeric probes specifically designed for chromosome 20 showed that both telomeric regions were preserved.  Analysis (F) showed 46, XX, r(20)(p13q13.3) in 8%. | mos 46,XX,r(20)(p13q13.3)/46,XX |
| Castori et al. 2012 | 28 | M |  |  |  |
| Cellini et al. 1998 | 29 | M | Hypopigmentation: Decreased functional activity in the melanocytes, which contained few rudimentary cytoplasmic projections and poorly developed organelles. Reduction in the number of mature melanosomes. |  |  |
| Chitayat et al. 1990 | 30 | M |  | Analyses (F+L) showed mosaic 46,XY/47,XY+18 in 2/3 biopsies. | mos 46,XY/47,XY,+18 |
| Cho et al. 2011 | 31 | M | Hyperpigmentation: Increased melanin in the basal layer.  Hypopigmentation: Decreased melanin content.  Immunohistochemistry with melanocytic markers S-100 protein and HMB-45 showed slightly decreased melanocytes in the hypopigmented patch. |  |  |
| Cho et al. 2012 | 32-61 | 14 F/  16 M | Hyperpigmentation: Increased level of melanin pigment in the basal cell layer compared with adjacent normal skin, although no significant difference existed in the number of melanocytes.  Pigmentary incontinence in 13/30 cases. |  |  |
| Choi et al. 2005 | 62 | M | Increased pigmentation within the basal keratinocytes. A few dermal melanophages. |  |  |
| Cohen et al. 2014 | 63-98 | 23 F/  13 M |  |  |  |
| Correa-Cerro et al. 1997 | 99 | F |  | Analysis (L) showed a balanced translocation, 46,X,t(X;13)(Xp13q;Xq13p). | 46,X,t(X;13)(Xp13q;Xq13p) |
| Delaporte et al. 1996 | 100 | M | Biopsy from hyperpigmented skin was divided into three fragments:   - First fragment: abnormally dense hyperpigmentation in the entire basal layer. Melanin pigments also seen. No dyskeratotic cell in the epidermis and no vacuolar alteration of the epidermal basal layer. - Second fragment: melanocytes were isolated with few small cytoplasmic extensions. - TEM: Normal maturation pattern in the melanocytes and a distribution to all basal keratinocytes in small aggregates containing 2-3 melanosomes. No giant melanosome. In the perivascular macrophages, most lysosomes contained melanosomes. | Analysis (L) showed normal karyotype. | 46,XY |
| Desai et al. 1988 | 101 | M |  |  |  |
| Devillers et al. 2011 | 102 | M | Hypopigmentation: Fontana–Masson staining for melanin was globally negative with rare foci of discrete staining.  Normopigmentation: Melanin staining more evenly positive in the basal layer. Tyrosinase immunoreactivity was weak to absent, restricted to a few cells focally clustered in tiny portions of the basal layer.  TEM of hypopigmented skin: Melanocytes contained fewer single or compound melanosomes looking immature and poorly melanized. Some melanosomes contained striated or zigzag pleated coiled inclusions, others were distorted. Immature or aberrant melanosomes present in keratinocytes of the basal layer of the epidermis were engulfed in prominent phagosomes. | Analysis showed normal karyotype. | 46,XY |
| Dhar et al. 2009 | 103 | F |  | First analysis (L) was normal. Second analysis (L) showed an additional inverted chromosome 13q2 in 1/100 cells.  Analysis (F)(B) showed an inverted duplicated chromosome 13q21 in 16/20 (85%), the remaining 4/20 showed normal karyotype  FISH studies with whole chromosome 13 paint confirmed the duplication to be chromosome 13 in origin.  High resolution array-CGH using the Agilent 244K oligo array on DNA (F) showed a gain in copy number of chromosome region 13q21-q33 of approximately 48.52 Mb. The 244K array was also performed on DNA sample from a buccal swab, which was reported as normal. | mos 46,XX,inv.dup(13q21)/46,XX |
| Di Lernia 2007 | 104-119 | 6 F/  10 M | Mild increase of melanin in the basal layer and mild elongation of the rete ridges. |  |  |
| Di Lernia 2015 | 120 | M | Hypopigmentation: Nonspecific histologic changes. | Analysis (F)(B) showed a trisomy 7 cell line in 20% of the cells. | mos 46,XY/47,XY,+7 |
| Donnai et al. 1988 | 121 | F |  | Analysis (L) showed normal karyotype.  Analysis (F) showed mixed diploid and triploid cells (84% triploid). | mos 69,XXX/46,XX or  mos 69,XXY/46,XX |
|  | 122 | F |  | Analysis (L) showed normal karyotype.  Analysis (F) showed mixed diploid and triploid cells (41% triploid). | mos 69,XXX/46,XX or  mos 69,XXY/46,XX |
|  | 123 | M |  | Analyses (F+L) showed normal karyotype. | 46,XY |
| Dúran-McKinster et al. 2002 | 124 | F |  | Analysis (L) showed normal karyotype.  Analysis (F): NA. | 46,XX |
| Eid et al. 2013 | 125 | F |  | Analysis (L) showed ring chromosome 15.  Confirmed by FISH, which showed deletion of subtelomere at the ring chromosome 15. | 46,XX,r(15) |
| El-Sawy et al. 2011 | 126 | F |  |  |  |
| Errichetti et al. 2016 | 127 | F | Hyperpigmentation: Increased basal layer pigmentation without melanocytosis. No pigment incontinence in the dermis. |  |  |
| Ertam et al. 2009 | 128 | F | Hyperpigmentation: Increased melanin pigmentation in the basal layer. Melanocytes were normal in size and number with Masson Fontana stain. No pigment incontinence in dermis. | Genetic analysis for the evaluation of a possible chimerism or mosaicism showed no abnormal findings. | 46,XY |
| Faletra et al.  2012 | 129 | M |  | Analysis of amniocytes showed 47,XY+mar in 13/64 metaphases and 51/64 metaphases showed normal karyotype.  Analysis (L) showed 2% of cells with the supernumerary marker.  SNP array analysis (F) showed a duplication involving the region q21.33-q34 of the chromosome 13. | mos 47,XY,+mar(13q21.33-q34)/46,XY |
|  | 130 | M |  | SNP array analysis (F) showed a high percentage of mosaicism (80%) characterized by a deletion of around 76 Mb of the long arm of chromosome 13. | mos 47,XY,+del(13)(q13.3q34)/46,XY |
| Fan et al. 1994 | 131 | F | Hypopigmentation: Decreased melanin pigment in the basal layer. | Analyses (F+L) showed normal karyotype. | 46,XX |
| Finkelstein et al. 1992 | 132 | F | Normopigmentation: No abnormalities on histological examination.  Hypopigmentation: Uniform decrease in the amount of melanin granules in the basal layer of the epidermis in comparison to normal skin. |  |  |
| Fleury et al. 1986 | 133-136 | 2 F/  2 M | Normopigmentation: Interruption of the basement membrane.  Hypopigmentation: Small giant cells were seen in the epidermis. |  |  |
| Fogu et al. 2008 | 137 | F |  |  |  |
| Fritz et al. 1998 | 138 | F |  | Analyses (F+L) showed 46,XX/47,XX,+r(X)(p21.3q13).  (F) showed an additional r(X) in one cell line with no evidence of X-chromosome loss and Turner syndrome.  The proportion of mosaicism was different in the two tissues examined. The normal cell line was predominant (80–93% in (L), and 70–90% in (F)).  FISH analysis with chromosome-X-specific DNA probes showed that the ring was derived from an X chromosome. The breakpoints could be localized in the long arm proximal to the X-inactivation centre in Xq13 and in the short arm distal to the DMD gene in Xp21.2–21.3. | mos 47,XX,+r(X)(p21.3q13)/46,XX |
| Fujimoto et al. 1985 | 139 | F |  | Analyses (F+L) showed mos 46,XX/46,XX,-1.5,+t(14;15)(q11;p11).  The trisomic cell line was present in 20% and 32% of the cells analysed in two blood specimens.  The trisomic cell line was found only in the specimen obtained from the right side of the body in 2%. | mos 46,XX/46,XX,-1.5,+t(14;15)(q11;p11) |
| Fujino et al. 1995 | 140 | F |  | Analyses of amino acids in urine, serum, and chromosomes were normal. | 46,XX |
| Garcia Muret et al. 2002 | 141 | M |  |  |  |
| George et al. 1992 | 142 | F | Hypopigmentation: Reduction in melanin content. |  |  |
| Gerdes et al. 2006 | 143 | F |  | Analysis (L) showed normal karyotype.  Analysis (F) showed a marker chromosome in 29/30 analysed metaphases from the hyperpigmented skin and 48/50 analysed metaphases from normal skin.  MBAND and MFISH analyses confirmed that the marker chromosome is an isochromosome 12p. | mos 47,XX,+i(12)(q10)/46,XX |
| Gonzalez-del Angel et al. 2014 | 144 | F |  | Two analyses (L) showed mosaic 47,XX,+13/46,XX.  Analysis (F) showed mosaic 47,XX,+13[7]/ 46,XX[78] in light skin samples and mosaic 47,XX,+13[23]/46,XX[2] in dark skin samples. | mos 47,XX,+13/46,XX |
| Gonzalez-Ensenat et al. 2009 | 145 | F |  | Analysis (L) showed normal karyotype.  Analysis (F) showed mos 47,XX,+der(13)i(13)(q21-qter)/46,XX.  FISH: 13 ish tel13q. | mos 47,XX,+der(13)i(13)(q21-qter)/ 46,XX |
|  | 146 | F |  | Analysis (L) showed normal karyotype.  Analysis (F) showed 47,XX+mar. rev.ish enh(13)(q22qter). Confirmed by FISH. | 47,XX+mar. rev.ish enh(13)(q22qter) |
| Grazia et al. 1993 | 147 | F | Hypopigmentation: Reduced amount of melanin granules in the basal layer of the dermis.  TEM: Reduced number of melanosomes in the basal melanocytes, which were also smaller in size. | Analysis (L) showed trisomy 18 mosaicism with the trisomic cell line in 73%.  Analysis (F)(B) showed trisomy 18 mosaicism with the trisomic cell line in 12%.  Analysis (F)(N) showed trisomy 18 mosaicism with the trisomic cell line in 18%. | mos 46,XX/47,XX,+18 |
| Griebel et al. 1989 | 148 | F |  |  | 46,XX |
|  | 149 | F |  |  | 46,XX |
|  | 150 | M |  |  |  |
|  | 151 | F |  |  |  |
| Gupta et al. 2007 | 152 | F |  | Analysis (L) showed normal karyotype.  Analysis (F) showed 47,XX,+2[9]/46,XX[11] | mos 47,XX,+2/46,XX |
| Gutte 2014 | 153 | M | Hyperpigmentation: Uniformly increased pigmentation within the basal keratinocytes. Few dermal melanophages with mild pigment incontinence without inflammatory infiltrate in the dermis. Nevus cells were absent. |  |  |
| Hansen et al. 2003 | 154 | F |  | Analysis (L) showed normal karyotype.  Analysis (F) from two independent cell lines showed one cell line with 47 chromosomes and one cell line with normal karyotype.  FISH identified the supernumerary marker chromosome as isochromosome 5p. | mos 47,XX,+i(5)(q10)/46,XX |
| Hansen et al. 2010 | 155 | M |  | Analysis (L) of two independent tests showed normal karyotype in 15 and 100 metaphases.  Analysis (F) showed trisomy 7 mosaicism in of 23%(N) and 4% (B). | mos 47,XY,+7/46,XY |
| Happle et al. 1997 | 156 | M |  | Analysis (L) and (F)(A+B) showed normal karyotype. | 46,XY |
| Happle 2009 | 157 | F |  |  |  |
|  | 158 | F |  |  |  |
|  | 159 | F |  |  |  |
|  | 160 | M |  | Analysis (L) and two buccal smears showed normal karyotype. | 46,XY |
| Happle et al. 2012 | 161 | M |  | Analysis (L) showed normal karyotype. | 46,XY |
|  | 162 | F |  |  |  |
|  | 163 | F |  | Analysis (L) showed normal karyotype. | 46,XX |
|  | 164 | M |  |  |  |
| Hartmann et al. 2004 | 165 | M | Hyperpigmentation: Mild elongation of the rete ridges, hyperpigmentation of the basal layer, prominent melanocytes, scattered melanophages in the upper dermis. | Prenatal analysis of two cultures of amniocytes showed trisomy 20 mosaicism in 50% and 75%.  Analysis (L) showed normal karyptype.  Analysis (F) showed trisomy 20 in 17% of metaphases and normal karyotype in 83%. | mos 46,XY,+20/46,XY |
| Hassab-El-Naby et al. 1996 | 166 | M | Hyperpigmentation: Increased melanin content in the basal cell layer with a normal number of melanocytes. | Analysis (L) showed normal karyotype. | 46,XY |
| Hernandez-Martin et al. 2014 | 167-191 | 12 F/  13 M | In 3 patients: Normal epidermis, mild perivascular dermal inflammatory infiltrate, scattered melanophages in the papillary dermis and no evidence of fungal organisms. |  |  |
| Hogeling et al. 2010 | 192-230 | 20 F/  19 M |  |  |  |
| Hong et al. 2008 | 231 | M | Hyperpigmentation: Increased melanin content at the basal layer without any increase in melanocyte and pigmentary incontinence. | Analysis (L) showed 46,XY with pericentric inversion of chromosome 9 between p11 and q12 loci in 100%, which is a normal variation. | 46,XY |
| Horn et al. 1997 | 232 | F |  | Analysis (L) showed mosaicism of 46,XX/47,XX,+13 in 4/100 metaphases.  Analysis (F) showed 47,XX,+13 in 7%. Confirmed by FISH. | mos 47,XX,+13/46,XX |
| Horn et al. 2002 | 233 | M |  | Analyses (F+L) showed mos 46,XY,dup(3)(p21.3;pter)/46,XY with 12 % aberrant cells in lymphocytes and 2% in skin fibroblast. | mos 46,XY,dup(3)(p21.3;pter)/46,XY |
|  | 234 | M |  |  | 46,XY |
| Ishikawa et al. 1985 | 235 | M | Hypopigmentation: No specific histologic changes. | Chromosomal analysis showed mosaicism of 45,XY,-14,-21,+t(14q 21q) and 46,XY,-14,-21,+t(14q 21q)+mar with the ratio of 1:2. | mos 45,XY,rob(14;21)(q10;q10)/46,XY,rob(13;14)(q10;q10),+mar |
| Jagia et al. 2004 | 236 | F | Hyperpigmentation: Increase in the melanin content within the keratinocytes of the basal layer, with focal areas of increase in the number of melanocytes.  Hypopigmentation: Normal number of melanocytes on S-100 staining. |  |  |
| Jain et al. 2012 | 237 | M | Increased pigmentation of the basal layer without incontinence of the pigment. |  | 46,XY |
| Jenkins et al. 1993 | 238 | M |  | Analysis (F) showed trisomy 7 in 26/36 cells. | mos 46,XY/47,XY,+7 |
| Kalter et al. 1988 | 239 | F | Hyperpigmentation: Diffuse basal layer hyperpigmentation, mild lentiginous elongation of some rete pegs.  No giant melanosomes or incontinence of pigment.  TEM: Normal melanocytes and melanosomes. Difference between dark and light skin was a relative increase in melanosomes in the former. | Analyses (F+L) showed normal karyotype | 46,XX |
|  | 240 | M | Hyperpigmentation: Irregularly increased pigmentation of basal cell layer with prominent melanocytes. No pigmentary incontinence or dermal melanophages.  TEM: Normal melanocytes and melanosomes. Difference between dark and light skin is a relative increase in melanosomes in the former. | Analyses (F+L) showed normal karyotype. | 46,XY |
| Kang et al. 1996 | 241 | M | Hypopigmentation: No melanocytes, neither with Fontana-Masson stain.  TEM: Number of melanosomes was decreased in melanocytes, but no change in size of melanocytes. Melanosomes were reduced in number in keratinocytes compared to normal pigmented skin. |  |  |
| Kanwar et al. 1993 | 242 | M | Hyperpigmentation: Diffuse basal cell hyperpigmentation with increase in number of basal melanocytes. No basal cell degeneration, incontinence of pigment, melanophages in dermis, tissue eosinophilia, or giant melanosomes. |  |  |
| Kayser et al. 2000 | 243 | M |  | Analysis (L) showed normal karyotype.  Analysis (F) showed 43% with trisomy 7 and 57% with normal karyotype. Second test showed 3% with trisomy 7 and 97% with normal karyotype. | mos 47,XY,+7/46,XY |
| Keng et al. 2006 | 244 | M |  | Analysis (L) showed normal karyotype.  Analysis (F)(N) showed 8% of cells with balanced translocation mos 46,XY,t(1;9)(q21 or q23;q22)/46,XY.  FISH showed the translocation in 5% of 200 buccal cells. | mos 46,XY,t(1;9)(q21 or q23;q22)/46,XY |
| Khandpur et al. 2006 | 245 | M |  |  |  |
| Kiritsi et al. 2015 | 246 | M |  |  |  |
| Koiffmann et al. 1993 | 247 | F |  | Cytogenetic studies (50 cells) showed 46,X,t(X;10)(pll;qll)mat. The pattern of X inactivation after BrdU incorporation for 6 hours was studied in 50 metaphases from patient and mother. In all the studied cells, the normal X was late labelling. | 46,X,t(X;10)(pll;qll)mat |
| Kosaki et al. 2008 | 248 | M |  |  |  |
| Kroisel et al. 2000 | 249 | M |  | Analysis (L) showed mosaic 46,XY,trp(3)(q27.1-qter)/46,XY.  FISH using YAC-clones showed that the terminal triplication on the derivative chromosome 3 occurred because of an insertional inverted duplication. | mos 46,XY,trp(3)(q27.1-qter)/46,XY |
| Kubota et al. 1992 | 250 | F | Hyperpigmentation: Diffuse basal layer hyperpigmentation with a slight increase in the number of basal melanocytes. No pigmentary incontinence or dermal melanophages. | Analysis (L) showed three types of cell lines of sex chromosomal mosaicism, namely 45X/46X, mar-1/47X, mar-2, mar-2.  Q-banding of the Y-chromosome did not show fluorescence, which was considered caused by a deletion of the distal Yq. G-banding was compared to the father’s Y-chromosome, which showed that marker chromosome-1 was a ring of the Y-chromosome and marker chromosome-2 was composed of double Yp-terminals and double parts of Yq. | mos 45X/46X,mar-1/47X,mar-2,mar-2 |
| Kuwahara et al. 2001 | 251 | F |  |  | Trisomy 14^[[1]](#footnote-1)^ |
| Lal et al. 2015 | 252 | M | Mild increase of melanin in the basal layer of the epidermis and mild elongation of the rete ridges without dermal pigmentary incontinence. | Analysis (L) showed normal karyotype.  Analysis (F)(B) showed trisomy 4.  Analysis (F)(N) showed normal karyotype. | mos 47,XY,+4/46,XY |
| Larralde et al. 2005 | 253 | F |  |  |  |
| Leonard et al. 2002 | 254 | F |  | Analysis (L) in the newborn period showed normal karyotype (30 cells).  Analysis (F)(B) at age 17 showed 16% tetraploidy (3 cells), 11% translocation 46,XX, t(1;6)(p32;q13) (2 cells), 73% showed normal karyotype (14 cells).  (F)(A) showed normal karyotype (20 cells). | mos 46,XX,t(1;6)(p32;q13)/46,XX |
| Lipsker et al. 2008 | 255 | M |  | Analysis (L) showed that 50% were 46,XY and 50% were 46,XX.  Analysis (F)(B) showed 46,XY.  Blood group determination showed a double population.  HLA typing of blood lymphocytes showed the inheritance of two HLA haplotypes from the mother and one from the father. | mos 46,XX/46,XY |
| Llamas-Velasco et al. 2010 | 256 | F | Melanocytic lentiginous pattern without pigmentary incontinence. | Analysis (F)(A+N) showed normal karyotype. Confirmed by FISH. | 46,XX |
| Lu et al. 2007 | 257 | F | Hyperpigmentation: Increased pigmentation within the basal keratinocytes with focal incontinentia pigmenti. Notable lymphangiectasis in the mid part of dermis. | Analysis (L) and (F)(N+A) showed normal karyotype. | 46,XX |
| Lungarotti et al. 1991 | 258 | F |  | Analyses (F+L) showed translocation between X and 18 with breakpoint at Xp11. | 46,X,t(X;18)(p11;q23) |
| Magenis et al. 1999 | 259 | M |  | Analysis (L) showed normal karyptype.  Analysis (F) of 411 fibroblasts: 71 % were normal, 14 % had an extra chromosome 7, 15 % had an extra ring 7, and two cells had both the extra 7 and extra ring.  Abnormal fibroblasts were detected in both pigmented and nonpigmented areas from both sides of the body.  FISH confirmed trisomy 7 and the chromosome 7 origin of the ring. | mos 47,XY,+7/47,XY,+r(7)/48,XY,+7,+r(7)/  46,XY |
| Maruani et al. 2012 | 260 | M | Hyperpigmentation: Increased melanin content in basal and suprabasal layers, no pigment incontinence in the dermis. |  |  |
| Mégarbané et al. 2002 | 261 | F |  | Analyses (F+L) showed normal karyotype. | 46,XX |
| Mendiratta et al. 2001 | 262 | F | Hyperpigmentation: Increased epidermal pigmentation without basal cell degeneration, pigmentary incontinence or dermal melanophages. |  |  |
| Metta et al. 2011 | 263 | F |  | Analyses (F+L) showed normal karyotype. | 46,XX |
|  | 264 | F | Hyperpigmentation: Increased pigmentation of the basal cell layer with melanocytes present up to the mid epidermis. Focal areas of pigmentary incontinence in the dermis. | Analyses (F+L) showed trisomy on chromosome 20. | 47,XX,+20 |
|  | 265 | F |  | Analyses (F+L) showed normal karyotype. | 46,XX |
| Meyer et al. 2004 | 266 | M |  |  |  |
| Morava et al. 2003 | 267 | F |  | Analyses (F+L) showed three different cell lines: r(15) in >90% of cells, monosomy 15 in 1-5%, and double ring in 3%. | mos 45,XX,-15/46,XX,r(15)/  47,XX,r(15),+r(15)  UPD15 |
| Morigaki et al. 2012 | 268 | F |  | Analysis (L) showed normal karyotype. | 46,XX |
| Muhammed et al. 2007 | 269 | M |  |  |  |
| Murano et al. 1991 | 270 | F |  | Analysis (L) at 15 years showed 3 cells with normal karyotype and 92 cells with 47,XX,+18.  Analysis (L) at 19 years showed all 96 cells with trisomy 18.  Analysis (F) showed 3 cells with normal karyotype and 43 cells with trisomy 18. | mos 47,XX,+18/46,XX |
|  | 271 | M |  | Analysis (L) showed normal/trisomy 18 mosaicism with 40% trisomic cells.  Analysis (F)(A) showed 27% trisomic cells from pigmented skin, but no abnormalities in (F) from normopigmented skin. | mos 47,XY,+18/46,XY |
| Myers et al. 2015 | 272 | F | Hypopigmentation: Fewer melanocytes in the stratum basale compared to biopsy from normal skin. | Analysis (F)(B) showed 39/50 fibroblasts with trisomy 13 (derivative chromosome 13 with an inverted duplication with breakpoints at 13q21.1 and 13q34.).  FISH confirmed duplication of the 13q34 region with deletion of 13q14 in the derivative chromosome 13.  Array-CGH confirmed the duplication and deletion in the derivative chromosome 13.  11/50 showed normal female karyotype.  Analysis (F)(N) showed similar distribution of abnormal (35) and normal (15) cells. | mos 47,XX,+inv dup(13)(qter->q21.1::q21.1->qter)/46,XX |
| Naveen et al. 2014 | 273 | M |  |  |  |
| Nehal et al. 1996 | 274-327 | 31 F/  23 M |  |  |  |
| Nicita et al. 2012 | 328 | F |  |  |  |
| Niessen et al. 2005 | 329 | F | Hyperpigmentation: Normal melanin pattern in the basal layer. | Analysis (L) showed 45,X karyotype in 29 examined metaphases.  Analysis (F) showed mosaic 47,XX,+7/45,X. FISH confirmed the mosaic pattern. | mos 47,XX,+7/45,X |
| Nishimura et al. 1998 | 330 | F |  | Analysis (L) showed normal karyotype. | 46,XX |
| Ogunbiyi et al. 1998 | 331 | F | Hypopigmentation: Essentially normal looking epidermis and dermis with no melanin incontinence. |  |  |
| Ohashi et al. 1992 | 332 | M |  | Analysis (L) and (F)(A+B) showed normal karyotype. | 46,XY |
|  | 333 | F |  | Analysis (L) and (F)(A+B) showed normal karyotype. | 46,XX |
|  | 334 | F |  | Analysis (L) and (F)(A+B) showed normal karyotype. | 46,XX |
|  | 335 | F |  | Analysis (L) and (F)(A+B) showed normal karyotype. | 46,XX |
|  | 336 | M |  | Analysis (L) and (F)(A+B) showed 46,XY/47,XY,+13. | mos 47,XY,+13/46,XY |
|  | 337 | F |  | Analysis (L) and (F)(A+B) showed 46,XX/47,XX,+14. | mos 47,XX,+14/46,XX |
|  | 338 | F |  | Analysis (L) and (F)(A+B) showed 46,XX/47,XX, +mar. | mos 47,XX,+mar/46,XX |
| Oiso et al. 2009 | 339 | F |  | Analysis (L) showed balanced X; autosome translocations 46,X,t(X; 9)(p11.21; q34.1). | 46,X,t(X;9)(p11.21;q34.1) |
| Oiso et al. 2010 | 340 | M | Hyperpigmentation: Elongated rete ridges with basal hyperpigmentation. Fontana-Masson staining showed excess melanin granules in the basal layer and melanin granules persistent in the epidermis. Immunohistochemical staining with HMB45 showed a positive reaction to the basal keratinocytes.  TEM: Presence of stage IV melanosomes with a scattered distribution in the keratinocytes. | Analysis (L) showed 46,XY,r(13)(p11.2q34) in 21 cells, 45,XY,–13 in 7 cells and 46,XY,dicr(13)(p11.2q34) in 2 cells. | mos 45,XY,-13/46,XY,r(13)(p11.2q34)/  46,XY,dic r(13)(p11.2q34) |
| Oiso et al. 2014 | 341 | M |  |  |  |
| Ong et al. 1985 | 342 | F |  |  |  |
| Ousager et al. 2006 | 343 | M |  | Analysis (L) and (F)(N) showed normal karyotype.  (F)(A) in two independent cell cultures showed clones with trisomy 16 (11% and 8% of the analysed cells, respectively). Confirmed by FISH. | mos 46,XY,+16/46,XY |
| Ousager et al. 2012 | 344 | F | Papillomatous, hyperkeratosis and epidermal acanthosis compatible with the diagnosis of acanthotic keratinocytic EN and very similar to what seen in seborrhoiec keratosis. |  |  |
| Palungwachira et al. 2006 | 345 | M | Hypopigmentation: Slightly reduced number of melanocytes. Decrease of melanin granules within basal and malpighian keratinocytes and a lack of pigmented cells in the upper dermis.  TEM: Epidermal keratinocytes contained melanosomes of stage IV, which grouped to make melanosome complexes or existed as a single body. These melanosomes were rather small in size and decreased in numbers. The number of melanocytes was not decreased, but reduced pigment formation evidenced by the presence of stage II, III melanosomes was seen. |  |  |
| Pascual-Castroviejo et al. 1998 | 346-421 | 41 F/  35 M |  | Analysis (L) performed in 68/76 cases: 67 karyotypes were normal, 1 was abnormal.  Analysis (F)(B+N) performed in 8/76 failed to grow. | 67/76 Normal karyotype  1/76 Abnormal karyotype  8/76 NA |
| Patil et al. 2012 | 422 | F |  | Analysis (L) showed low level of mosaicism for trisomy 9. | mos 47,XX,+9/46, XX  UPD9 |
| Pellegrino et al. 1995 | 423 | M | Border between the hypo- and hyperpigmented areas showed no specific changes. | Cytogenetic analysis demonstrated two populations of cells: a normal male cell line and a line containing 45 chromosomes with an unbalanced rearrangement between chromosome 7 and 15, 45,XY,-7,-15,+der(7)t(7;15)(q34;ql 3)/46,XY.  Analysis (F) of 100 metaphases: 80 showed normal karyotype, 20 had the unbalanced t(7;15).  Analysis (L): 20% had the unbalanced t(7;15). | mos 45,XY,-7,-15,+der(7)(7;15)t(q34;q13) /46,XY |
| Petit et al. 2012 | 424 | F |  | Foetal analysis showed normal karyotype.  Analysis (L) showed normal karyotype.  Analysis (F)(N+B) showed mosaicism for trisomy 7. | mos 47,XY,+7/46,XY  UPD7 |
| Pillay et al. 2013 | 425 | F | Hypopigmentation (compared to normopigmentation): Adequate number of melanocytes. Decreased amount of melanin pigment within the melanocytes and keratinocytes. No evidence of pigmentary incontinence.  TEM: Melanocytes contained sparse membrane-bound melanosome complexes. Few single melanosomes were present. Melanosomes present in the keratinocytes were sparse compared to normal skin. The melanocytes profiles appeared rounded in shape and closely related to the basal lamina. | Analysis (L) showed trisomy 13 with translocation of chromosome 13 on 13: 46,XX,t(13:13) in 17 metaphases.  Analysis (F)(A+B) showed the same translocation, isochromosome 13 in 20 metaphases. | 46,XX,t(13:13) |
| Pinheiro et al. 2007 | 426-451 | 12 F/  14 M | Performed on hyperpigmented skin in 9/26 patients:   - 2/9: Pigment incontinence. - 1/9: Minimal pigment incontinence. - 6/9: Increase in pigmentation of the basal layer but no evidence of pigment incontinence. | Analysis (L) performed in 6/26 with hyperpigmentation showed normal karyotype in 4/6. 2/6 showed trisomy 21. | 4/6 Normal karyotype  2/6 Trisomy 21 |
| Pini et al. 1995 | 452 | M |  |  |  |
| Pinto de Gouveia et al. 2016 | 453 | M |  | Analyses (F)(A+B) showed trisomy 7 in 5% of the analysed fibroblasts. | mos 47,XY,+7/46,XY |
| Ponti et al. 2014 | 454 | F |  | Analysis (L) showed normal karyotype. | 46,XX |
|  | 455 | M |  | Analysis (L) showed normal karyotype.  Analysis (F)(B) showed trisomy 2 in 16%. | mos 47,XY,+2/46,XY |
| Portnoï et al. 1999 | 456 | M | Hyperpigmentation: Diffuse basal layer hyperpigmentation. | Analyses showed two populations of cells: a normal male cell line and a line containing a supernumerary marker chromosome.  In (L) the marker chromosome was present in 30% of the metaphases.  In (F)(A) the marker chromosome was present in 6%.  FISH with whole chromosome painting probes and YAC specific clones found the marker to consist of duplicated chromosome material from the distal part of chromosome 3q and was interpreted as inv.dup(3). | mos 47,XY,+mar/46,XY ish inv.dup(3)(qter🡪q27.1::q27.1🡪qter) |
| Pulimood et al. 1997 | 457 | M |  |  |  |
|  | 458 | M |  |  |  |
| Quecedo et al. 1997 | 459 | M | Hyperpigmentaion: Increase in the melanin content of the basal layer, but no dermal melanin deposits or melanophages. | Analyses (F+L) showed normal karyotype. | 46,XY |
| Quigg et al. 2006 | 460 | M |  |  |  |
| Ravel et al. 2001 | 461 | M |  | Analysis (L) showed trisomy 9 in 20% of metaphases.  Analysis (F) showed trisomy 9 in 5% of metaphases. | mos 47,XY,+9/46,XY |
| Resende et al. 2013 | 462 | F |  |  |  |
| Ribeiro Noce et al. 2001 | 463 | F |  | Analysis (L) showed translocation involving chromosome 13 with 46,XX,−13,+t(13q;13q) in all 50 cells.  Analysis (F) showed 3 different abnormal cell lines with 45,XX,-13,+frag present in 74% of cells, 46,XX,-13,+t(13q;13q) present in 14% of cells, and 45,XX,-13 present in 12% of cells. | mos 45,XX,-13+frag/46,XX,-13,+t(13q;13q)/  45,XX,-13 |
| Ritter et al. 1990 | 464 | M | Hypopigmentation: Decreased melanin. | Analysis (L) and (F)(N) showed mos 46,XY,r(22)/46,XY.  Analysis (F)(B) showed normal karyotype. | mos 46,XY,r(22)/46,XY |
| Rittinger et al. 2008 | 465 | F |  | Analysis (L) showed normal karyotype.  Analysis (F) showed a triploid karyotype (69,XXX) in 15/18 metaphases and a normal karyotype in the remaining three metaphases. | mos (69,XXX)/46,XX |
| Romano et al. 1999 | 466 | F | Hyperpigmentation: Normal architecture without inflammatory changes, widespread hyperpigmentation of the basal layer of the epidermis and mild hyperplasia of the melanocytes. No incontinence of pigment observed.  TEM: Basal keratinocytes contained an increased number of clustered melanosomes. Melanocytic dendrites containing melanosomes in the periphery were observed between keratinocytes. No melanophages in the dermis. |  |  |
| Rott et al. 1990 | 467 | M | Hypopigmentation: Reduced pigmentation in the basal cell layer compared to normal skin. | Analysis (L) and (F)(B) showed normal karyotype.  Analysis (F)(N) showed 46,XY in 122 metaphases, but small ring chromosome (47,XY,+r) in 2 metaphases. | mos 47,XY,+r/46,XY |
| Ruggieri 2000 | 468 | M | Hyperpigmentation: Increased melanin content of the basal layer. No dermal melanin or melanophages.  Hypopigmentation: Decreased melanin content and number of melanocytes. | Analyses (F+L) showed 46,XY.  NF2 polymorphic microsatellite markers were demonstrated. | 46,XY |
|  | 469 | M | Hyperpigmentation: Increased melanin content of the basal layer. No dermal melanin or melanophages.  Hypopigmentation: Decreased melanin content and number of melanocytes. | Analyses (F+L) showed normal karyotype. | 46,XY |
| Ruggieri et al. 2003 | 470 | F |  | Analyses (F)(A+B+N) showed normal karyotype.  Inv dup(15) syndrome excluded by FISH. | 46,XX |
| Ruggieri et al. 2009 | 471 | M |  | Analyses (F)(B+N) showed normal karyotype in 100 cell lineages examined five times. | 46,XY |
| Ruiz-Maldonado et al. 1992 | 472-512 | 21 F/  20 M | Hypopigmentation (compared to normopigmentation): Reduced number of epidermal melanocytes and of their melanin content, and increased number of large epidermal clear cells. Decreased number of epidermal melanocytes and melanosomes, and increased number of Langerhans cells. Miscellaneous alterations in 21 patients. | Analysis (L):  16/19 were normal.  3/19 showed chromosomal translocation X/I5, pericentric inversion of chromosome 2, and Turner syndrome mosaicism 45,X/46,X,r(X). | 16/19 Normal karyotype  46,X,t(X;15)  46,XX,inv(2) or 46,XY,inv(2)  mos 45,X/46,X,r(X) |
| Sarma 2012 | 513-580 | 29 F/  39 M |  |  |  |
| Saxena et al. 1989 | 581 | F | Hypopigmentation (compared to normopigmentation): No abnormality in the epidermis and dermis except for reduction in the number of melanocytes.  TEM: Two distinct types of melanocytes:   - Highly dendritic cells with numerous branching processes. The cytoplasm of the dendritic processes contained nonmelanised and partially melanised premelanosomes as compared with the control site. - Rounded melanocytes with no dendritic processes, a large nucleus, and a thin rim of cytoplasm containing occasional melanised melanosomes, which varied in size, shape and level of melanisation. |  |  |
| Schepis et al. 1996 | 582 | M | Hyperpigmentation: Prominent melanocytes, increase of melanin in the epidermis.  Normopigmentation: Prominent and widely distributed melanocytes, increase of melanin in the epidermis.  TEM:  Hyperpigmentation: Increased number of melanocytic cytoplasmic prolongations, no increase of melanocytic profiles. The prolongations contained mature melanosomes located mainly underneath the plasma membrane. The keratinocytes showed mild vacuolar degeneration. In occasional high power fields, in basal keratinocytes, a reduplication of the basement membrane was observed.  Normopigmentation: Melanocytes with dispersed chromatin, abundant rough endoplasmic reticulum, well developed melanosomes, and bundles of intermediate filaments of stage III and IV. Keratinocytes appeared well preserved except for occasional cytoplasmic vacuolization. |  |  |
| Schepis et al. 1999 | 583 | M | Hyperpigmentaion: Moderate melanic pigmentation of the basal layer, without pigmentary incontinence. |  | 46,XY |
| Schepis et al. 2001 | 584 | F |  | Analyses (F+L) showed mosaic with trisomy 13 in 25% and 70% respectively. | mos 47,XX,+13/46,XX |
| Scott et al. 2008 | 585 | M | Normal skin biopsy. |  | 46,XY |
| Shah et al. 2012 | 586 | M |  | Analysis (L) showed normal karyotype.  Analysis (F)(A) showed 47,XY,+i(12)(p10)[5]/46,XY[15] and (F)(B) showed 47,XY,+i(12) (p10)[8]/46,XY[12]. FISH analysis showed four copies of the locus on chromosome 12p. | mos 47,XY,+i(12)(p10)/46,XY |
| Sharma et al. 2008 | 587 | M |  |  |  |
| Shimizu et al. 2013 | 588 | F |  |  |  |
|  | 589 | M |  |  |  |
| Sigurdardottir et al. 1999 | 590 | F |  | Analysis (L) showed 46,XX,r(4) (p16.3q35.2).ish r(4)(4ptel+,4qtel+,AHT+) in 16/20 cells studied (80%). Confirmed by FISH. | mos 46,XX,r(4)(p16.3q35.2)/46,XX |
|  | 591 | M |  | Analysis (L) showed 45,XY,- 9[5]/46,XY,r(9)(p24q34.3)[45].ish.r(9) (9ptel+,9qtel+, AHT-). Confirmed by FISH. | mos 45,XY,-9/46,XY,r(9)(p24q34.3) |
| Singh et al. 2014 | 592 | M | Hypopigmentation: Unremarkable epidermis and dermis.  Bluish-grey area: Unremarkable epidermis and scattered dendritic melanized melanocytes in both the superficial and deep dermis within collagen bundles. | Analysis (L) showed normal karyotype. | 46,XY |
| Steijlen et al. 2000 | 593 | F |  | Analysis (L) showed normal karyotype. | 46,XX |
|  | 594 | F |  |  |  |
|  | 595 | F |  | Analysis (L) showed normal karyotype. | 46,XX |
|  | 596 | M |  |  |  |
|  | 597 | F |  | Analysis (L) showed normal karyotype. | 46,XX |
| Stoll et al. 2002 | 598 | M | Hyperpigmentation and normopigmentation: Normal structure i.e. melanocytes were morphologically and quantitatively normal. The difference between normal and hyperpigmented skin was quantitative regarding to melanic pigmentation. | Analysis (F)(N) showed that a translocation was present in 70% of the cells examined: 46,XY,t(12:14)(q233-q22.1). Karyotype was normal in 30% of the cells.  Spectral FISH study and chromosome painting with WCP 12 and WCP 14 confirmed the result. | mos 46,XY,t(12:14)(q233-q22.1)/46,XY |
| Strømme et al. 2005 | 599 | F |  | Analysis (L) showed normal karyotype.  Analysis (F) showed 47,XX,+20 in 4/24 cells in one culture, and 5/10 cells in another. | mos 47,XX,+20/46,XX  UPD20 |
| Sybert et al. 1990 | 600 | F |  |  |  |
|  | 601 | F |  | Analyses (F+L) performed twice showed normal karyptype. | 46,XX |
|  | 602 | F |  | Analysis (F) performed twice showed normal karyotype. | 46,XX |
|  | 603 | F |  | Analyses (F+L) showed normal karyotype. | 46,XX |
|  | 604 | M |  | Analyses (F+L) showed normal karyotype. | 46,XY |
|  | 605 | F |  | Analysis (L) showed 46,X,t(X;22)(q11.2;q13.3) balanced. | 46,X,t(X;22)(q11.2;q13.3) |
|  | 606 | F |  | Analysis (L) showed 46,XX,r(10). | 46,XX,r(10) |
|  | 607 | F |  | Analysis (L) showed mos 45,XX,t(15;22)/46,XX. | mos 45,XX,t(15;22)/46,XX |
|  | 608 | F |  | Analysis (L) showed mos 45,X/46,X,+ring. | mos 45,X/46,X,+ring |
|  | 609 | M |  | Analyses (F+L) showed mos 47,XY,+18/46,XY. | mos 47,XY,+18/46,XY |
|  | 610 | F |  | Analysis (L) showed normal karyotype.  Analysis (F) showed mos 46,XX,del(13)(q11)/46,XX. | mos 46,XX,del(13)(q11)/46,XX |
|  | 611 | M |  | Analysis (L) showed normal karyotype.  Analysis (F) showed mos 69,XXY/46,XY. | mos 69,XXY/46,XY |
|  | 612 | M |  | Analysis (L) showed normal karyotype.  Analysis (F) showed mos 47,XY+i(12p)/46,XY. | mos 47,XY+i(12p)/46,XY |
| Taibjee et al. 2009 | 613 |  |  | Analyses (F+L) showed normal karyotype. | Normal karyotype |
|  | 614 |  |  | Analyses (F+L) showed normal karyotype. | Normal karyotype |
|  | 615 |  |  | Analyses (F+L) showed normal karyotype. | Normal karyotype |
|  | 616 |  |  | Analyses (F+L) showed normal karyotype. | Normal karyotype |
|  | 617 |  |  | Analyses (F+L) showed normal karyotype. | Normal karyotype |
|  | 618 | F |  | Analysis of keratinocytes from light skin showed trisomy 20 mosaicism in 9/14 metaphases.  Analysis of keratinocytes from dark skin showed normal karyotype.  Analysis (F)(A+B) showed normal karyotype. Confirmed by FISH. | mos 47,XX,+20⁄46,XX |
|  | 619 |  |  | Analyses (F+L) showed normal karyotype. | Normal karyotype |
|  | 620 |  |  | Analyses (F+L) showed normal karyotype. | Normal karyotype |
|  | 621 |  |  | Analyses (F+L) showed normal karyotype. | Normal karyotype |
|  | 622 |  |  | Analyses (F+L) showed normal karyotype. | Normal karyotype |
| Thapa et al. 2007 | 623 | M |  |  |  |
| Thomas et al. 1989 | 624 | F |  | Analyses (F+L) showed four cell lines: a normal line, monosomy 14, a pseudoisodicentic 14q, and a ring 14. | mos 45,XX,-14/46,XX,i(14)(q10)/  46,XX,r(14)/46,XX |
|  | 625 | F |  | Analysis (L) showed 45,X or 45,X+acentric fragment.  Analysis (F) showed mos 45,X/46,X,del(X). | mos 45,X/46,X,del(X) |
|  | 626 | M |  | Analysis (L) showed mos 47,XYY/48,XYY,+8.  Analysis (F) showed 48,XYY,+8. | mos 47,XYY/48,XYY,+8 |
|  | 627 | F |  | Analyses (F+L) showed mos 46,XX,4p+/46,XX. | mos 46,XX,4p+/46,XX |
|  | 628 | F |  | Analysis (L) showed mos 46,XX,18p-/46,XX.  Analysis (F) showed 46,XX,18p-. | mos 46,XX,18p-/46,XX |
|  | 629 | F |  | Analyses (F+L) showed mos 45,X/46,r(Y). | mos 45,X/46,r(Y) |
|  | 630 | F |  | Analysis (L) showed 45,X.  Analysis (F) showed mos 45,X/46,XY. | mos 45,X/46,XY |
|  | 631 | M |  | Analyses (F+L) showed mos 45,X/46,XY. | mos 45,X/46,XY |
| Toelle et al. 2006 | 632 | F |  |  |  |
| Toll et al. 2007 | 633 | F | Transitional lines: Segmental loss of eccrine sweat glands. |  |  |
| Trägårdh et al. 2014 | 634 | M |  |  |  |
| Tsutsumi et al. 1991 | 635 | F |  |  |  |
| Tunca et al. 2000 | 636 | F |  | Analysis (L) of 100 metaphases showed normal karyotype.  Analysis (F)(A) showed mos 46,XX,add(14)(q32)[16]/46,XX[44]. Confirmed by FISH. | mos 46,XX,add(14)(q32)/46,XX |
| Turleau et al. 1986 | 637 | F |  | Analysis (L): In approximately 50% of the lymphocytes a microdeletion of 15ql was obvious but it was impossible to decide whether it involved q11 or q13. In the other 50% of the cells this region appeared normal or difficult to analyse.  In the abnormal cells the banding pattern was very similar to that seen in Prader-Willi syndrome. | mos 46,XX,del(15)(q11or13)/46,XX |
| Verghese et al. 1999 | 638 | M |  | Analysis (L) showed normal karyotype.  Analysis (F) showed trisomy 7 in 28/35 cells (80%). Seven cells had normal karyotype. | mos 47,XY,+7/46,XY |
| Vormittag et al. 1992 | 639 | F | Hypopigmentation: Basal cell pigmentation varied from normal to increased, and markedly decreased to completely missing pigmentation. | Analysis (L) showed normal karyotype.  Analysis (F) showed no structural chromosomal anomaly in #1.  (F)(B) showed 46,XX/92,XXXX: a high rate of tetraploidy (22%) in #2, which decreased continuously in the course of passaging. (F)(N) showed a very low rate of tetraploidy in all passages analysed. | mos 46,XX/92,XXXX |
|  | 640 | F | Hypopigmentation: Basal cell pigmentation varied from normal to increased, and markedly decreased to completely missing pigmentation. | Analysis (L) showed normal karyotype. | 46,XX |
| Weaver et al. 1991 | 641 | F |  | Analysis showed normal karyotype. | 46,XX |
| Woods et al. 1994 | 642 | M |  | Analysis (L) showed normal karyotype in 146 cells and 46,XX,r(22) in 5 cells.  Analysis (F)(N) showed ring chromosome 22 in 11/60 cells. | mos 46,XX,r(22)/46,XY |
|  | 643 | F |  | Analysis (L) in two separate examinations showed normal karyotype.  Analysis (F)(N) showed mosaicism with a large interstitial deletion of the long arm of chromosome 8, 46,XX,del(8)(q21q24) in 77/80 cells. | mos 46,XX,del(8)(q21q24)/46,XX |
|  | 644 | F | Hypopigmentation: No dermal hypoplasia. Dermal collagen was mature, no inflammatory infiltrate. Pigment along basal layer was decreased. No evidence of pigmentary incontinence.  TEM: No decrease in melanocyte numbers, but melanosomes and melanisation were diminished. | Analysis (L) of 30 cells showed normal karyotype.  Analyses (F)(B+N) showed normal karyotype in 48/50 cells, trisomy 21 in 1/50 and 46,XX,t(11;14)(p11;q12) in 1/50.  Analysis (F)(B) showed three cell lines: Normal karyotype in 9/50, a balanced translocation between chromosome 11 and 19 in 36/50, and a pericentric inversion of chromosome 7 and a balanced translocation between 4 and 5 in 5/50 cells. | mos 46,XX,t(11;19)(q21;p13.1)/  46,XX,inv(7)(p14q36),t(4;5)(q31;q35)/  46,XX |
|  | 645 | F |  | Analysis showed mosaicism with 43/51 cells showing 46,XX,del(18)(q21q23) and 8 cells showing the same karyotype with the addition of a small marker chromosome. | mos 46,XX,del(18)(q21q23)/  46,XX,del(18)(q21q23)+mar |
|  | 646 | F |  | Analysis (L) showed normal karyotype.  Analysis (F)(A) showed 20 normal female cells and 10 with trisomy 22. | mos 47,XX,+22/46,XX |
| Wulfsberg et al. 1991 | 647 | F |  | Analysis (F) showed a mosaic chromosomal pattern with 65% normal karyotype and 35% 69,XXX in 3 separate cultures. | mos 69,XXX/46,XX |
|  | 648 | F |  | Analysis (F) showed normal karyotype. | 46,XX |
| Yakinci et al. 2002 | 649 | M |  | Analysis (L) showed mosaic 46,XY,+13,der(13;13)(q10;q10)/46,XY. | mos 46,XY,+13,der(13;13)(q10;q10)/  46,XY |
| Yim et al. 1996 | 650 | F | Hyperpigmentation: Increased pigmentations of the basal layer without pigmentary incontinence or dermal melanophages.  Hypopigmentation: Normal pigmentation of the basal layer.  TEM: Evenly distributed, mature melanosomes on keratinocytes. | Analyses (L) and (F)(A+B) showed normal karyotype. | 46,XX |
| Yuksek et al. 2007 | 651 | M | Hyperpigmentation: Diffuse hyperpigmentation of the basal layer and mild elongation of rete ridges. |  |  |

1. Given no further information [↑](#footnote-ref-1)
