# Supplementary material for: Pigmentary mosaicism: a review of original literature and recommendations for future handling
Source: Orphanet J Rare Dis. 2018 Mar 5;13:39. doi: 10.1186/s13023-018-0778-6 (PMC5839061; doi:10.1186/s13023-018-0778-6)
Supplement: Supplementary file 4 — Supplemental bibliography. (DOCX 52 kb) [file 13023_2018_778_MOESM4_ESM.docx]

Additional file 4: Supplemental bibliography

1. Afsar FS, Aktas S, Ortac R. Becker’s naevus and segmental naevus depigmentosus: An example of twin spotting? Australas J Dermatol. 2007;48:224-6.
2. Aguayo-Leiva I, Alonso J, Echeverría B, Hernández-Martín A, Torrelo A. Phacomatosis melanovascularis: a new example of non-allelic twin spotting. Eur J Dermatol. 2011;21:487-9.
3. Akahoshi K, Spritz RA, Fukai K, Mitsui N, Matsushima K, Ohashi H. Mosaic supernumerary inv dup(15) chromosome with four copies of the P gene in a boy with pigmentary dysplasia. Am J Med Genet A. 2004;126A:290-2.
4. Akiyama M, Aranami A, Sasaki Y, Ebihara T, Sugiura M. Familial linear and whorled nevoid hypermelanosis. J Am Acad Dermatol. 1994;30:831-3.
5. Al Aboud A, Al Aboud K, Al Hawsawi K, Al Aboud D, Ramesh V. Pigmentary mosaicism of Hyperpigmented Type in Two Sisters. Skinmed 2005;4:120-1.
6. Alrobaee AA, Alsaif F. Linear and whorled nevoid hypermelanosis associated with developmental delay and generalized convulsions. Int J Dermatol. 2004;43:145-7.
7. Alvarez J, Peteiro C, Toribio J. Linear and whorled nevoid hypermelanosis. Pediatr Dermatol. 1993;10:156-8.
8. Baba M, Akçali C, Seçkin D, Happle R. Segmental lentiginosis with ipsilateral nevus depigmentosus: another example of twin spotting? Eur J Dermatol. 2002;12:319-21.
9. Baba M, Seçkin D, Akçali C, Happle R. Familial cutis tricolor: a possible example of paradominant inheritance. Eur J Dermatol. 2003;13:343-5.
10. Ballmer-Weber BK, Inaebnit D, Brand CU, Braathen LR. Sporadic hypomelanosis of Ito with focal hypertrichosis in a 16-month-old girl. Dermatology. 1996;193:63-4.
11. Bartholomew DW, Jabs EW, Levin LS, Ribovich R. Single maxillary central incisor and coloboma in hypomelanosis of Ito. Clin Genet. 1987;31:370-3.
12. Baty BJ, Olson SB, Magenis RE, Carey JC. Trisomy 20 mosaicism in two unrelated girls with skin hypopigmentation and normal intellectual development. Am J Med Genet. 2001;99:210-6.
13. Bocian E, Mazurczak T, Bulawa E, Stanczak H, Rowicka G. Triple structural mosaicism of chromosome 18 in a child with MR/MCA syndrome and abnormal skin pigmentation. J Med Genet. 1993;30:614-15.
14. Boente Mdel C, Bazan C, Montanari D. Cutis tricolor parvimaculata in two patients with ring chromosome 15 syndrome. Pediatr Dermatol. 2011;28:670-3.
15. Boon C, Markello T, Jackson-Cook C, Pandya A. Partial trisomy 10 mosaicism with cutaneous manifestations: report of a case and review of the literature. Clin Genet. 1996;50:417-21.
16. Brar BK, Mahajan BB, Puri N. Linear and whorled nevoid hypermelanosis. Indian J Dermatol Venereol Leprol. 2008;74:512-3.
17. Brock JA, Dyack S, Ludman M, Dumas N, Gaudet M, Morash B. Mosaic tetrasomy 5p resulting from an isochromosome 5p marker chromosome: a case report and review of literature. Am J Med Genet A. 2012;158A:406-11.
18. Bygum A, Fagerberg CR, Clemmensen OJ, Fiebig B, Hafner C. Systemic epidermal nevus with involvement of the oral mucosa due to FGFR3 mutation. BMC Med Genet. 2011;12:79.
19. Bygum A, Petkov Y, Graakjaer J, Jensen UB, Fagerberg C. Phylloid hypermelanosis in a child with psychomotor delay, cicatricial alopecia, hearing loss and polythelia. Acta Derm Venereol. 2012;92:191-2.
20. Capaldi L, Gray J, Abuelo D, Torrelo A, Nieto J, Lapidus C, et al. Pigmentary mosaicism and mosaic Turner syndrome. J Am Acad Dermatol. 2005;52:918-9.
21. Cappanera S, Passamonti C, Zamponi N. New association between ring chromosome 20 syndrome and hypomelanosis of Ito. Pediatr Neurol. 2011;45:341-3.
22. Castori M, Scarciolla O, Morlino S, Manente L, Biscaglia A, Fragasso A, et al. Whorled hairless nevus of the scalp, linear hyperpigmentation, and teleangiectatic nevi of the lower limbs: a novel variant of the “phacomatosis complex”. Am J Med Genet A. 2012;158A:445-9.
23. Cellini A, Morroni M, Simonetti O, Offidani A. Hypomelanosis of Ito: a case report with clinical and ultrastructural data. J Eur Acad Dermatol Venereol. 1998;10:73-6.
24. Chitayat D, Friedman JM, Johnston MM. Hypomelanosis of Ito – a nonspecific marker of somatic mosaicism: report of case with trisomy 18 mosaicism. Am J Med Genet. 1990;35:422-4.
25. Cho E, Cho SH, Lee JD. Patterned pigmentation in a child: a case of segmental pigmentation disorder. J Dermatol. 2011;38:1094-6.
26. Cho E, Cho SH, Lee JD. Progressive cribriform and zosteriform hyperpigmentation: a clinicopathologic study. Int J Dermatol. 2012;51:399-405.
27. Choi JC, Yang JH, Lee UH, Parks HS, Chun DK. Progressive and zosteriform hyperpigmentation - the late onset linear and whorled nevoid hypermelanosis. J Eur Acad Dermatol Venereol. 2005;19:638-9.
28. Cohen J, Shahrokh K, Cohen B. Analysis of 36 cases of Blaschkoid dyspigmentation: reading between the lines of Blaschko. Pediatr Dermatol. 2014;31:471-6.
29. Correa-Cerro LS, Rivera H, Vasquez AI. Functional Xp disomy and de novo t(X;13)(q10;q10) in a girl with hypomelanosis of Ito. J Med Genet. 1997;34:161-3.
30. Delaporte E, Janin A, Blondel V, Copin MC, Piette F, de Martinville B, et al. Linear and whorled nevoid hypermelanosis versus incontinentia pigmenti: is pigmentary incontinence really a distinctive feature? Dermatology. 1996;192:70-2.
31. Desai PC, Banerjee KS, Chaturvedi P. Hypomelanosis of Ito (incontinentia pigmenti achromians). Indian Pediatr. 1988;25:1000-2.
32. Devillers C, Quatresooz P, Hermanns-Lê T, Szepetiuk G, Lemaire R, Piérard-Franchimont C, et al. Hypomelanosis of Ito: pigmentary mosaicism with immature melanosome in keratinocytes. Int J Dermatol. 2011;50:1234-9.
33. Dhar SU, Robbins-Furman P, Levy ML, Patel A, Scaglia F. Tetrasomy 13q mosaicism associated with phylloid hypomelanosis and precocious puberty. Am J Med Genet A. 2009;149A:993-6.
34. Di Lernia V. Linear and whorled hypermelanosis. Pediatr Dermatol. 2007;24:205-10.
35. Di Lernia V. Patterned hypopigmentation associated with prenatally diagnosed trisomy 7 mosaicism: long-term follow-up. J Dtsch Dermatol Ges. 2015;13:914-6.
36. Donnai D, Read AP, McKeown C, Andrews T. Hypomelanosis of Ito: a manifestation of mosaicism or chimerism. J Med Genet. 1988;25:809-18.
37. Durán-McKinster C, Moises C, Rodríguez-Jurado R, Tamayo-Sánchez L, Orozco-Covarrubias L, Ruiz-Maldonado R. Streptococcal exanthema in a blaschkolinear pattern: clinical evidence for genetic mosaicism in hypomelanosis of Ito. Pediatr Dermatol. 2002;19:423-5.
38. Eid MM, El-Bassyouni HT, Eid OM, Hamad SA, Elgerzawy A, Zaki MS, et al. Ring chromosome 15: expanding the phenotype. Genet Couns. 2013;24:417-25.
39. El-Sawy T, He L, Chiang MF, Anyane-Yeboa K, Morel KD, Folberg R, et al. Retinoblastoma presenting in a child with hypomelanosis of Ito. Open Ophthalmol J. 2011;5:55-8.
40. Errichetti E, Pegolo E, Stinco G. Linear and whorled nevoid hypermelanosis: A case report with dermoscopic findings. Indian J Dermatol Venereol Leprol. 2016;82:91-3.
41. Ertam I, Turk BG, Urkmez A, Kazandi A, Ozdemir F. Linear and whorled nevoid hypermelanosis: dermatoscopic features. J Am Acad Dermatol. 2009;60:328-31.
42. Faletra F, Berti I, Tommasini A, Pecile V, Cleva L, Alberini E, et al. Phylloid pattern of hypomelanosis closely related to chromosomal abnormalities in the 13q detected by SNP array analysis. Dermatology. 2012;225:294-7.
43. Fan PC, Wang PJ, Huang SF, Shen YZ. Hypomelanosis of Ito associated with West syndrome: report of a case. J Formos Med Assoc. 1994;93:429-32.
44. Finkelstein E, Shinwell E, Avinoach I, Hallel-Halevy D, Halevy S. Hypomelanosis of Ito: report of two cases. Australas J Dermatol. 1992;33:97-101.
45. Fleury P, Dingemans K, de Groot WP, Oranje AP, Voûte PA, Woerdeman MJ, et al. Ito’s hypomelanosis (incontinentia pigmenti achromians). A review of four cases. Clin Neurol Neurosurg. 1986;88:39-44.
46. Fogu G, Maserati E, Cambosu F, Moro MA, Poddie F, Soro G, et al. Patau syndrome with long survival in a case of unusual mosaic trisomy 13. Eur J Med Genet. 2008;51:303-14.
47. Fritz B, Küter W, Orstavik KH, Naumova A, Spranger J, Rehder H. Pigmentary mosaicism in hypomelanosis of Ito. Further evidence for functional disomy of Xp. Hum Genet. 1998;103:441-9.
48. Fujimoto A, Lin MS, Korula SR, Wilson MG. Trisomy 14 mosaicism with t(14;15)(q11;p11) in offspring of a balanced translocation carrier mother. Am J Med Genet. 1985;22:333-42.
49. Fujino O, Hashimoto K, Fujita T, Enokido H, Komatsuzaki H, Asano G, et al. Clinico-neuropathological study of incontinentia pigmenti achromians – an autopsy case. Brain Dev. 1995;17:425-7.
50. García Muret MP, Puig L, Allard C, Alomar A. Hypomelanosis of Ito with Sturge-Weber syndrome-like leptomeningeal angiomatosis. Pediatr Dermatol. 2002;19:536-40.
51. George AO, Adeyanju MO. Incontinentia pigmenti achromians (Ito) in a West African. East Afr Med J. 1992;69:227-8.
52. Gerdes AM, Hansen LK, Brandrup F, Soegaard K, Christoffersen A, Rasmussen K. Pallister-Killian syndrome: Multibrand FISH of tetrasomy 12p. Pediatr Dermatol. 2006;23:378-81.
53. González-del Angel A, Estandia-Ortega B, Gavino-Vergara A, Sáez-de-Ocariz M, Velasco-Hernández Mde L, Salas-Labadía C. A patient with trisomy 13 mosaicism with an unusual skin pigmentary pattern and prolonged survival. Pediatr Dermatol. 2014;31:580-3.
54. González-Ensenat MA, Vicente A, Poo P, Catalá V, Mar Pérez-Iribarne M, Fuster C, et al. Phylloid hypomelanosis and mosaic partial trisomy 13: two cases that provide further evidence of a distinct clinicogenetic entity. Arch Dermatol. 2009;145:576-8.
55. Grazia R, Tullini A, Rossi PG, Neri I, Patrizi A, Croci G, et al. Hypomelanosis of Ito with trisomy 18 mosaicism. Am J Med Genet. 1993;45:120-1.
56. Griebel V, Krägeloh-Mann I, Michaelis R. Hypomelanosis of Ito – report of four cases and survey of the literature. Neuropediatrics. 1989;20:234-7.
57. Gupta S, Shah S, Mcgaw A, Mercado T, Zaslav AL, Tegay D. Trisomy 2 mosaicism in hypomelanosis of Ito. Am J Med Genet A. 2007;143A:2466-8.
58. Gutte RM. Progressive cribriform and zisteriform hyperpigmentation. Indian Dermatol Online J. 2014;5:38-40.
59. Hansen LK, Brandrup F, Rasmussen K. Pigmentary mosaicism with mosaic chromosome 5p tetrasomy. Br J Dermatol. 2003;149:414-6.
60. Hansen LK, Bygum A, Krogh LN. Infantile spasm and pigmentary mosaicism. Epilepsia. 2010;51:1317-8.
61. Happle R, Barbi G, Eckert D, Kennerknecht I. “Cutis tricolor”: congenital hyper- and hypopigmented macules associated with a sporadic multisystem birth defect: an unusual example of twin spotting? J Med Genet. 1997;34:676-8.
62. Happle R. Monoallelic expression on autosomes may explain an unusual heritable form of pigmentary mosaicism: a historical case revisited. Clin Exp Dermatol. 2009;34:834-7.
63. Happle R, Franco-Guío MF, Santacoloma-Osorio G. Phylloid hypermelanosis: a cutaneous marker of several different disorders? Pediatr Dermatol. 2014;31:504-6.
64. Hartmann A, Hofmann UB, Hoehn H, Broecker EB, Hamm H. Postnatal confirmation of prenatally diagnosed trisomy 20 mosaicism in a patient with linear and whorled nevoid hypermelanosis. Pediatr Dermatol. 2004;21:636-41.
65. Hassab-El-Naby HM, Alsaleh QA, Fathallah MA. Linear and whorled nevoid hypermelanosis: report of a case associated with cerebral palsy. Pediatr Dermatol. 1996;13:148-50.
66. Hernández-Martín A, Gilliam AE, Baselga E, Vicente A, Lam J, González-Ensenat M, et al. Hyperpigmented macules on the face of young children: a series of 25 cases. J Am Acad Dermatol. 2014;70:288-90.
67. Hogeling M, Frieden IJ. Segmental pigmentation disorder. Br J Dermatol. 2010;162:1337-41.
68. Hong SP, Ahn SY, Lee WS. Linear and whorled nevoid hypermelanosis: unique clinical presentations and their possible association with chromosomal abnormality inv(9). Arch Dermatol. 2008;144:415-6.
69. Horn D, Rommeck M, Sommer D, Körner H. Phylloid pigmentary pattern with mosaic trisomy 13. Pediatr Dermatol. 1997;14:278-80.
70. Horn D, Happle R, Neitzel H, Kunze J. Pigmentary mosaicism of the hyperpigmented type in two half-brothers. Am J Med Genet. 2002;112:65-9.
71. Ishikawa T, Kanayama M, Sugiyama K, Katoh T, Wada Y. Hypomelanosis of Ito associated with benign tumors and chromosomal abnormalities: a neurocutaneous syndrome. Brain Dev. 1985;7:45-9.
72. Jagia R, Mendiratt V, Koranne RV, Sardana K, Bhushan P, Solanki RS. Colocalized nevus depigmentosus and lentigines with underlying breast hypoplasia: a case of reverse mutation? Dermatol Online J. 2004;10:12.
73. Jain P, Chakrabarty B, Gulati S. Linear and whorled nevoid hypermelanosis with hemiatrophy. Indian Pediatr. 2012:49:936.
74. Jenkins D, Martin K, Young ID. Hypomelanosis of Ito associated with mosaicism for trisomy 7 and apparent ‘pseudomosaicism’ at amniocentesis. J Med Genet. 1993;30:783-4.
75. Kalter DC, Griffiths WA, Atherton DJ. Linear and whorled nevoid hypermelanosis. J Am Acad Dermatol. 1988;19:1037-44.
76. Kang IK, Hann SK. Vitiligo coexistent with nevus depigmentosus. J Dermatol. 1996;23:187-90.
77. Kanwar AJ, Dhar S, Ghosh S, Kaur S. Linear and whorled nevoid hypermelanosis. Int J Dermatol. 1993;32:385-6.
78. Kayser M, Henderson LB, Kreutzman J, Schreck R, Graham JM Jr. Blaschkolinear skin pigmentary variation due to trisomy 7 mosaicism. Am J Med Genet. 2000;95:281-4.
79. Keng WT, Harewood L, Grace E, Paxton C, Lam WW, Fitzpatrick DR. A balanced reciprocal translocation in a case of hypomelanosis of Ito with confirmation of mosaicism using buccal cell interphase FISH. Am J Genet A. 2006;140:1111-3.
80. Khandpur S, Minz A, Sharma VK. An unusual association of pigmentary mosaicism (hypomelanosis of Ito) with generalized hypertrichosis. Clin Exp Dermatol. 2006;31:467-8.
81. Kiritsi D, Lorente AI, Happle R, Bernabeu Wittel J, Has C. Blaschko line acne on pre-existent hypomelanosis reflecting a mosaic FGFR2 mutation. Br J Dermatol. 2015;172:11257.
82. Koiffmann CP, de Souza DH, Diament A, Ventura HB, Alves RS, Kihara S, Wajntal A. Incontinentia pigmenti achromians (hypomelanosis of ITO, MIM146150): further evidence of localization at Xp11. Am J Med Genet. 1993;46:529-33.
83. Kosaki R, Naito Y, Torii C, Takahashi T, Nakajima T, Kosaki K. Split hand foot malformation with whorl-like pigmentary pattern: phenotypic expression of somatic mosaicism for the p63 mutation. Am J Med Genet A. 2008;146A:2574-7.
84. Kroisel PM, Petek E, Wagner K. Skin pigmentary anomalies in a mosaic form of partial tetrasomy 3q. J Med Genet. 2000;37:723-5.
85. Kubota Y, Shimura Y, Shimada S, Tamaki K, Amamiya S. Linear and whorled nevoid hypermelanosis in a child with chromosomal mosaicism. Int J Dermatol. 1992;31:345-7.
86. Kuwahara RT, Henson T, Tunca Y, Wilroy SW. Hyperpigmentation along the lines of Blaschko with associated chromosome 14 mosaicism. Pediatr Dermatol. 2001;18:360-1.
87. Lal K, Di Lernia V. Linear and whorled naevoid hypermelanosis in a patient with trisomy 4 mosaicism. Clin Exp Dermatol. 2015;40:45-7.
88. Larralde M, Happle R. Cutis tricolor parvimaculata: a distinct neurocutaneous syndrome? Dermatology. 2005;211:149-51.
89. Leonard NJ, Tomkins DJ. Diploid/tetraploid/t(1;6) mosaicism in a 17-year-old female with hypomelanosis of Ito, multiple congenital anomalies, and body asymmetry. Am J Med Genet. 2002;112:86-90.
90. Lipsker D, Flory E, Wiesel ML, Hanau D, de la Salle H. Between light and dark, the chimera comes out. Arch Dermatol. 2008;144:327-30.
91. Llamas-Velasco M, Eguren C, Arranz E, Renedo M, de Argila D, García-Díez A. Linear and whorled nevoid hypermelanosis and Axenfeld-Rieger anomaly: a novel association. Acta Derm Venereol. 2010;90:317-8.
92. Lu Y, Zhu WY. Linear and whorled nevoid hypermelanosis complicated with inflammatory linear verrocous epidermal nevus and ichthyosis vulgaris. J Dermatol. 2007;34:765-8.
93. Lungarotti MS, Martello C, Calabro A, Baldari D, Mariotti G. Hypomelanosis of Ito associated with chromosomal translocation involving Xp11. Am J Med Genet. 1991;40:447-8.
94. Magenis E, Webb MJ, Spears B, Opitz JM. Blaschkolinear malformation syndrome in complex trisomy-7 mosaicism. Am J Med Genet. 1999;87:375-83.
95. Maruani A, Khallouf R, Machet MC, Lorette G. Diffuse linear and whorled nevoid hypermelanosis in a newborn. J Pediatr. 2012;160:171.
96. Mégarbané A, Vabres P, Slaba S, Smahi A, Loeys B, Okais N. Linear and whorled nevoid hypermelanosis with bilateral giant cerebral aneyrysms. Am J Med Genet. 2002;112:95-8.
97. Mendiratta V, Sharma RC, Arya L, Sardana K. Linear and whorled nevoid hypermelanosis. J Dermatol. 2001;28:58-9.
98. Metta AK, Ramachandra S, Sadath N, Manupati S. Linear and whorled nevoid hypermelanosis in three successive generations. Indian J Dermatol Venereol Leprol. 2011;77:403.
99. Meyer CH, Freyschmidt-Paul P, Happle R, Kroll P. Unilateral linear hyperpigmentation of the skin with ipsilateral sectorial hyperpigmentation of the retina. Am J Med Genet A. 2004;126A:89-92.
100. Morava E, Bartsch O, Czako M, Frensel A, Kárteszi J, Kosztolányi GY. A girl with cutaneous hyperpigmentation, café au lait spots and ring chromosome 15 without significant deletion. Genet Couns. 2003;14:337-42.
101. Morigaki R, Pooh KH, Shouno K, Taniguchi H, Endo S, Nakagawa Y. Choroid plexus papilloma in a girl with hypomelanosis of Ito. J Neurosurg Pediatr. 2012;10:182-5.
102. Muhammed K, Mathew J. Coexistence of two neurocutaneous syndromes: tuberous sclerosis and hypomelanosis of Ito. Indian J Dermatol Venereol Leprol. 2007;73:43-5.
103. Murano I, Ohashi H, Tsukahara M, Tonoki H, Okino F, Atsumi M, et al. Pigmentary dysplasias in long survivors with mosaic trisomy 18: report of two cases. Clin Genet. 1991;39:68-74.
104. Myers JN Jr, Davis L, Sheehan D, Kulharya AS. Mosaic tetrasomy 13q and phylloid hypomelanosis: a case report and review of the literature. Pediatr Dermatol. 2015;32:263-6.
105. Naveen KN, Reshme P. Linear and whorled nevoid hypermelanosis with dermatoscopic features. Dermatol Online J. 2014;20:13.
106. Nehal KS, PeBenito R, Orlow SJ. Analysis of 54 cases of hypopigmentation and hyperpigmentation along the lines of Blaschko. Arch Dermatol. 1996;132:1167-70.
107. Nicita F, Spalice A, Roggini M, Papetti L, Ursitti F, Tarani L, et al. Complex malformation (Ruggieri-Happle) phenotype with “cutis tricolor” in a 10-year-old girl. Brain Dev. 2012:34:869-72.
108. Niessen RC, Jonkman MF, Muis N, Hordijk R, van Essen AJ. Pigmentary mosaicism following the lines of Blaschko in a girl with a double aneuploidy mosaicism: (47,XX,+7/45,X). Am J Med Genet A. 2005;137A:313-22.
109. Nishimura G, Nagai T. A case of craniofacial dysmorphism, ongenital heart defects, coccygeal skin folds, generalized skeletal alterations, and hemihypertrophy with linear skin hypopigmentation: a new syndrome? J Hum Genet. 1998;43:65-8.
110. Ogunbiyi AO, Ogunbiyi JO. Nevus depigmentosus and inflammatory linear epidermal nevus – an unusual combination with a note on history. Int J Dermatol. 1998;37:600-2.
111. Ohashi H, Tsukahara M, Murano I, Naritomi K, Nishioka K, Miyake S, et al. Pigmentary displasias and chromosomal mosaicism: report of 9 cases. Am J Med Genet. 1992;43:716-21.
112. Oiso N, Amatsu A, Kawara S, Kawada A. Pigmentary mosaicism with hyperpigmented streaks on the palmoplantar lesion associated with balanced X; autosome translocations t(X; 9)(p11.21; q34.1). J Eur Acad Dermatol Venereol. 2009;23:359-61.
113. Oiso N, Tsuruta D, Imanishi H, Sayasa H, Narita T, Kobayashi H, et al. Phylloid hypermelanosis and melanocytic nevi with aggregated and disfigured melanosomes: causal relationship between phylloid pigment distribution and chromosome 13 abnormalities. Dermatology. 2010;220:169-72.
114. Oiso N, Kawada A. Pigmentary mosaicism of the hypopigmented type (hypomelanosis of Ito): hypopigmented lesions with serrated and irregular borders. Eur J Dermatol. 2014;24:690-1.
115. Ong B, Chan HL, Cheah JS. Insulin dependent diabetes and goitre in a case of hypomelanosis of Ito (Incontinentia Pigmenti Achromians). Ann Acad Med Singapore. 1985;14:317-9.
116. Ousager LB, Brandrup F, Brasch-Andersen C, Erlendsson A. Skin manifestations in a case of trisomy 16 mosaicism. Br J Dermatol. 2006;154:172-6.
117. Ousager LB, Bygum A, Hafner C. Identification of a novel S249C FGFR3 mutation in a keratinocytic epidermal naevus syndrome. Br J Dermatol. 2012;167:202-4.
118. Palungwachira P, Palungwachira P. Incontinentia pigmenti achromians of Ito: an ultrastructural study. J Med Assoc Thai. 2006;89:253-7.
119. Pascual-Castroviejo I, Roche C, Martinez-Bermejo A, Arcas J, Lopez-Martin V, Tendero A, et al. Hypomelanosis of ITO. A study of 76 infantile cases. Brain Dev. 1998;20:36-43.
120. Patil SJ, Ponnala R, Shah S, Dalal A. Mosaic trisomy 9 presenting with congenital heart disease, facial dysmorphism and pigmentary skin lesions: intricate issues of genetic counselling. Indian J Pediatr. 2012;79:806-9.
121. Pellegrino JE, Schnur RE, Kline R, Zackai EH, Spinner NB. Mosaic loss of 15q11q13 in a patient with hypomelanosis of Ito: is there a role for the P gene? Hum Genet. 1995;96:485-9.
122. Pinto de Gouveia M, Coutinho I, Teixeira V, d’Oliveira R, Venâncio M, Moreno A. Do you know this syndrome? Dyspigmentation along the Blaschko lines caused by trisomy 7 mosaicism. An Bras Dermatol. 2016;91:837-9.
123. Petit F, Holder-Espinasse M, Duban-Bedu B, Bouquillon S, Boute-Benejean O, Bazin A, et al. Trisomy 7 mosaicism prenatally misdiagnosed and maternal uniparental disomy in a child with pigmentary mosaicism and Rusell-Silver syndrome. Clin Genet. 2012;81:265-71.
124. Pillay T, Winship WS, Ramdial PK. Pigmentary abnormalities in trisomy of chromosome 13. Clin Dysmorphol. 1998;7:191-4.
125. Pinheiro A, Mathew MC, Thomas M, Jacob M, Srivastava VM, Cherian R, et al. The clinical profile of children in India with pigmentary anomalies along the lines of Blaschko and central nervous system manifestations. Pediatr Dermatol. 2007;24:11-7.
126. Pini G, Faulkner LB. Cerebellar involvement in hypomelanosis of Ito. Neuropediatrics. 1995;26:208-10.
127. Ponti G, Pellacani G, Tomasi A, Percesepe A, Guarneri C, Guerra A, et al. Hypomelanosis of Ito with a trisomy 2 mosaicism: a case report. J Med Case Rep. 2014;8:333.
128. Portnoï MF, Boutchneï S, Bouscarat F, Morlier G, Nizard S, Dersarkissian H, et al. Skin pigmentary anomalies and mosaicism for an acentric marker chromosome originating from 3q. J Med Genet. 1999;36:246-50.
129. Pulimood S, Rajagopalan B, Jacob M, George S, Korah I. Hypomelanosis of Ito with unusual associations. Clin Exp Dermatol. 1997;22:295-6.
130. Quecedo E, Febrer I, Aliaga A. Linear and whorled nevoid hypermelanosis. A spectrum of pigmentary disorders.
131. Quigg M, Rust RS, Miller JQ. Clinical findings of the phakomatoses: hypomelanosis of Ito. Neurology. 2006;66:E45.
132. Ravel TJ, Legius E, Brems H, Van Hoestenberghe R, Gillis PH, Fryns JP. Hemifacial microsomia in two patients further supporting chromosomal mosaicism as a causative factor. Clin Dysmorphol. 2001;10:263-7.
133. Resende C, Araújo C, Vieira AP, Brito C. Late onset Ito’s nevus. BMJ Case Rep. 2013;30.05.
134. Ribeiro Noce T, de Pina-Neto JM, Happle R. Phylloid pattern of pigmentary disturbance in a case of complex mosaicism. Am J Med Genet. 2001;98:145-7.
135. Ritter CL, Steele MW, Wenger SL, Cohen BA. Chromosome mosaicism in hypomelanosis of Ito. Am J Med Genet. 1990;35:14-7.
136. Rittinger O, Kronberger G, Pfeifenberger A, Kotzot D, Fauth C. The changing phenotype in diploid/triploid mosaicism may mimic genetic sydromes with aberrant genomic imprinting: follow up in a 14-year-old girl. Eur J Med Genet. 2008;51:573-9.
137. Romano C, Pirrone P, Siragusa M, Schepis C, Cavallari V. An additional case of linear and whorled nevoid hypermelanosis associated with birth defects and mental retardation. Pediatr Dermatol. 1999;16:71-3.
138. Rott HD, Lang GE, Huk W, Pfeiffer RA. Hypomelanosis of Ito (incontinentia pigmenti achromians). Ophthalmological evidence for somatic mosaicism. Ophthalmic Pediatr Genet. 1990;11:273-9.
139. Ruggieri M. Cutis tricolor: congenital hyper- and hypopigmented lesions in a background of normal skin with and without associated systemic features: further expansion of the phenotype. Eur J Pediatr. 2000;159:745-9.
140. Ruggieri M, Iannetti P, Pavone L. Delineation of a newly recognized neurocutaneous malformation syndrome with “cutis tricolor”. Am J Med Genet A. 2003;120A:110-6.
141. Ruggieri M, Roggini M, Spalice A, Addis M, Iannetti P. Pigmentary mosaicism, subcortical band heterotopia, and brain cystic lesions. Pediatr Neurol. 2009;40:383-6.
142. Ruiz-Maldonado R, Toussaint S, Tamayo L, Laterza A, del Castillo V. Hypomelanosis of Ito: diagnostic criteria and report of 41 cases. Pediatr Dermatol. 1992;9:1-10.
143. Sarma N. Pigmentary nevi on face have unique patterns and implications: The concept of Blaschko’s lines for pigmentary nevi. Indian J Dermatol. 2012;57:30-4.
144. Saxena U, Ramesh V, Iyengar B, Misra RS. Hypomelanosis of Ito: histochemical and ultrastructural observations. Australas J Dermatol. 1989;30:45-7.
145. Schepis C, Siragusa M, Alberti A, Cavallari V. Linear and whorled nevoid hypermelanosis in a boy with mental retardation and congenital defects. Int J Dermatol. 1996;35:654-5.
146. Schepis C, Alberti A, Siragusa M, Romano C. Progressive cribriform and zosteriform hyperpigmentation: the late-onset feature of linear and whorled nevoid hypermelanosis associated with congenital neurological, skeletal and cutaneous anomalies. Dermatology. 1999;199:72-3.
147. Schepis C, Failla P, Siragusa M, Romano C. An additional case of macular phylloid mosaicism. Dermatology. 2001;202:73.
148. Scott A, Micallef C, Hale SL, Watts P. Cortical visual impairment in hypomelanosis of Ito. J Pediatr Ophthalmol Strabismus. 2008;45:240-1.
149. Shah K, Gerorge R, Balla ES, Oommen SP, Padankatti CS, Srivastava VM, et al. An Indian boy with additional features in Pallister-Killian syndrome. Indian J Pediatr. 2012;79:1238-40.
150. Sharma P, Pai HS, Kamath MM. Nevus depigmentosus affecting the iris and skin: a case report. J Eur Acad Dermatol Venereol. 2008;22:634-5.
151. Shimizu K, Makino T, Ueda C, Takegami Y, Matsui K, Mizawa M, et al. Detection of hypohidrosis in Japanese patients with pigmentary mosaicism. Eur J Dermatol. 2013;23:913-4.
152. Sigurdardottir S, Goodman BK, Rutberg J, Thomas GH, Jabs EW, Geraghty MT. Clinical, cytogenetic, and fluorescence in situ hybridization findings in two cases of “complete ring” syndrome. Am J Med Genet. 1999;87:384-90.
153. Singh N, Chandrashekar L, Thappa DM, Kar R. Nevus depigmentosus and nevus of Ito: pigmentary twin spotting. Int J Dermatol. 2014;53:1005-7.
154. Steijlen PM, Viëtor HE, Steensel MV, Happle R. Sweat testing in hypomelanosis of Ito: divergent results reflecting genetic heterogeneity. Eur J Dermatol. 2000;10:217-9.
155. Stoll C, Alembik Y, Grosshans E, de Saint Martin A. An unusual human mosaic for skin pigmentation. Genet Couns. 2002;13:281-7.
156. Strømme P, van der Hagen CB, Haakonsen M, Risberg K, Hennekam R. Follow-up of a girl with cleft lip and palate and multiple malformations: trisomy 20 mosaicism. Scand J Plast Reconstr Surg Hand Surg. 2005;39:178-9.
157. Sybert VP, Pagon RA, Donlan M, Bradley CM. Pigmentary abnormalities and mosaicism for chromosomal aberration: association with clinical features similar to hypomelanosis of Ito. J Pediatr. 1990;116:581-6.
158. Taibjee SM, Hall D, Balderson D, Larkins S, Stubbs T, Moss C. Keratinocyte cytogenetics in 10 patients with pigmentary mosaicism: identification of one case of trisomy 20 mosaicism confines to keratinocytes. Clin Exp Dermatol. 2009;34:823-9.
159. Thapa R, Dhar S, Malakar R, Chakrabartty S. Hypomelanosis of Ito – whorled hyperpigmentation combination: a mirror image presentation. Pediatr Dermatol. 2007;24:572-3.
160. Thomas IT, Frias JL, Cantu ES, Lafer CZ, Flannery DB, Graham JG Jr. Association of pigmentary anomalies with chromosomal and genetic mosaicism and chimerism. Am J Hum Genet. 1989;45:193-205.
161. Toelle SP, Boltshauser E, Wirth MG, Itin P. Association of lentiginous mosaicism and congenital cataract in a girl. Eur J Dermatol. 2006;16:360-2.
162. Toll A, Vincent MC, Calvas P, Pujol RM. V-shaped hyperpigmented linear lesions, patchy hypotrichosis, and teeth abnormalities in a young girl. Pediatr Dermatol. 2007;24:551-4.
163. Trägårdh M, Thomsen CR, Thorninger R, Møller-Madsen B. Hypomelanosis of Ito presenting with pediatric orthopedic issues: a case report. J Med Case Rep. 2014;8:156.
164. Tsutsumi T, Oguchi H. Labial talon cusp in a child with incontinentia pigmenti achromians: case report. Pediatr Dent. 1991;13:236-7.
165. Tunca Y, Wilroy RS, Kadandale JS, Martens PR, Gunther WM, Tharapel AT. Hypomelanosis of Ito and a ‘mirror image’ whole chromosome duplication resulting in trisomy 14 mosaicism. Ann Genet. 2000;43:39-43.
166. Turleau C, Taillard F Doussau de Bazignan M, Delépine N, Desbois JC, de Grouchy J. Hypomelanosis of Ito (incontinentia pigmenti achromians) and mosaicism for a microdeletion of 15q1. Hum Genet. 1986;74:185-7.
167. Verghese S, Newlin A, Miller M, Burton BK. Mosaic trisomy 7 in a patient with pigmentary abnormalities. Am J Med Genet. 1999;87:371-4.
168. Vormittag W, Ensinger C, Raff M. Cytogenetic and dermatoglyphic findings in a familial case of hypomelanosis of Ito (incontinentia pigmenti achromians). Clin Genet. 1992;41:309-14.
169. Weaver RG Jr, Martin T, Zanolli MD. The ocular changes of incontinentia pigmenti achromians (hypomelanosis of Ito). J Pediatr Ophthalmol Strabismus. 1991;28:160-3.
170. Woods CG, Bankier A, Curry J, Sheffield LJ, Slaney SF, Smith K, et al. Asymmetry and skin pigmentary anomalies in chromosome mosaicism. J Med Genet. 1994;31:694-701.
171. Wulfsberg EA, Wassel WC, Polo CA. Monozygotic twin girls with diploid/triploid chromosome mosaicism and cutaneous pigmentary dysplasia. Clin Genet. 1991;39:370-5.
172. Yakinci C, Kutlu NO, Alp MN, Senol M, Durmaz Y, Budak T. Hypomelanosis of Ito with trisomy 13 mosaicism [46, XY, der(13;13)(q10;q10), +13/46, XY]. Turk J Pediatr. 2002;44:152-5.
173. Yim SY, Lee IY, Rah UW, Moon HW, Hahn SH, Lee ES, et al. Linear and whorled nevoid hypermelanosis with delayed psychomotor development. Yonsei Med J. 1996;37:290-4.
174. Yuksek J, Sezer E, Erbil AH, Arca E, Tastan HB, Kurumlu Z, et al. Linear and whorled nevoid hypermelanosis. Dermatol Online J. 2007;13:23.
